# Expression analysis and mapping of Viral—Host Protein interactions of Poxviridae suggests a lead candidate molecule targeting Mpox

**DOI:** 10.1186/s12879-024-09332-x

**Published:** 2024-05-10

**Authors:** Tamizhini Loganathan, John Fletcher, Priya Abraham, Rajesh kannangai, Chiranjib Chakraborty, Achraf El Allali, Alsamman M. Alsamman, Hatem Zayed, George Priya Doss C

**Affiliations:** 1 Laboratory of Integrative Genomics, Department of Integrative Biology, School of Biosciences and Technology, Vellore Institute of Technology (VIT), Vellore-632014, Tamil Nadu, India; 2 Department of Clinical Virology, Christian Medical College, Tamil Nadu, Vellore, 632004 India; 3 School of Life Science and Biotechnology, Adamas University, Kolkata, India; 4 Bioinformatics Laboratory, College of Computing, Mohammed VI Polytechnic University, Ben Guerir, Mohammed Morocco; 5 Department of Genome Mapping, Molecular Genetics, and Genome Mapping Laboratory, Agricultural Genetic Engineering Research Institute, Giza, Egypt; 6 Department of Biomedical Sciences College of Health Sciences, QU. Health, Qatar University, Doha, Qatar

**Keywords:** Monkeypox, Histones, Immune genes, S3I-201, Bioinformatics

## Abstract

**Background:**

Monkeypox (Mpox) is an important human pathogen without etiological treatment. A viral-host interactome study may advance our understanding of molecular pathogenesis and lead to the discovery of suitable therapeutic targets.

**Methods:**

GEO Expression datasets characterizing mRNA profile changes in different host responses to poxviruses were analyzed for shared pathway identification, and then, the Protein–protein interaction (PPI) maps were built. The viral gene expression datasets of Monkeypox virus (MPXV) and Vaccinia virus (VACV) were used to identify the significant viral genes and further investigated for their binding to the library of targeting molecules.

**Results:**

Infection with MPXV interferes with various cellular pathways, including interleukin and MAPK signaling. While most host differentially expressed genes (DEGs) are predominantly downregulated upon infection, marked enrichments in histone modifiers and immune-related genes were observed. PPI analysis revealed a set of novel virus-specific protein interactions for the genes in the above functional clusters. The viral DEGs exhibited variable expression patterns in three studied cell types: primary human monocytes, primary human fibroblast, and HeLa, resulting in 118 commonly deregulated proteins. Poxvirus proteins C6R derived protein K7 and K7R of MPXV and VACV were prioritized as targets for potential therapeutic interventions based on their histone-regulating and immunosuppressive properties. In the computational docking and Molecular Dynamics (MD) experiments, these proteins were shown to bind the candidate small molecule S3I-201, which was further prioritized for lead development.

**Results:**

MPXV circumvents cellular antiviral defenses by engaging histone modification and immune evasion strategies. C6R-derived protein K7 binding candidate molecule S3I-201 is a priority promising candidate for treating Mpox.

**Supplementary Information:**

The online version contains supplementary material available at 10.1186/s12879-024-09332-x.

## Background

Poxviruses, large double-stranded DNA viruses, inhabit a wide ecological niche. The replication of these viruses is confined to the cytoplasm of the infected cell [1]. The viral genome produces a variety of proteins crucial for viral replication and evading the host's cellular and immune responses [2]. Orthopoxvirus, a Poxviridae family member that includes Variola virus (VARV) causing smallpox, Monkeypox virus (MPXV), and Cowpox virus (CPXV), is known to cause diseases in humans and remains a public health issue. Vaccinia virus (VACV) and CPXV continue to cause emerging endemic diseases, particularly in developing countries. Some examples of endemic diseases include chickenpox, malaria, polio, and rotavirus. Despite eradicating VARV, it remains the top priority for biodefense preparedness research [3]. MPXV, a member of the Orthopoxvirus genus, can cause sporadic human outbreaks and has been involved in an emerging pandemic across 30 countries. The MPXV has become so prevalent that the World Health Organization (WHO) has classified it as a global health crisis. The Centers for Disease Control and Prevention (CDC) has reported that the number of cases worldwide for the 2022–2023 outbreak stands at 93,497, with the United States accounting for 31,689 cases and 56 deaths [4]. The human Monkeypox (Mpox) outbreak, attributed to the West African lineage of the MPXV, encompasses the 2022–2023 outbreak in India. India was the inaugural South Asian case and the tenth in Asia to document a Mpox case. At present, India has reported 23 Mpox cases [5].

The genome of the MPXV, encompassing 196,858 base pairs, encodes 190 open reading frames, constituting most of the genetic information required for viral replication in the cell cytoplasm [6]. The entry of the virus into cells, contingent on the cell and viral strain, ensues after initial attachment to the cell surface through interactions with various viral ligands and cell surface receptors, including chondroitin sulfate [7]. Upon penetration into the cell cytoplasm, the virus discharges preloaded viral proteins and enzymatic factors that debilitate the cell's defenses and stimulate the expression of early genes. The synthesis of intermediary transcription factors, DNA replication, and subsequent uncoating are all propelled by early protein synthesis [8]. The transcription and translation of intermediate genes culminate in the expression of late genes, which typically serve as early transcription factors, enzymes, and structural proteins. Poxviruses have developed numerous tactics to evade the host's immune defenses, and the disease mechanisms of Monkeypox (Mpox) are still largely unknown.

Furthermore, no effective treatment is available to prevent infection by the Monkeypox Virus (MPXV). LC16, MVA-BN (JYNNEOSTM), and ACAM2000 vaccines are currently used. However, their adoption is limited due to potential adverse effects [9].

Expression datasets were obtained from the Gene Expression Omnibus (GEO), a public database, and differentially expressed genes were examined using a bioinformatics pipeline. We retrieved three different transcriptomics datasets from hosts (*Homo sapiens* (GSE36854 and GSE219036) and *Macaca mulatta* (GSE21001)) to identify the differentially expressed genes in response to infection. The viral gene expression datasets of MPXV and VACV (GSE11234) were used to identify the significant viral genes.

Histone genes were consistently overrepresented in various gene expression studies.

**Fig. 1:**
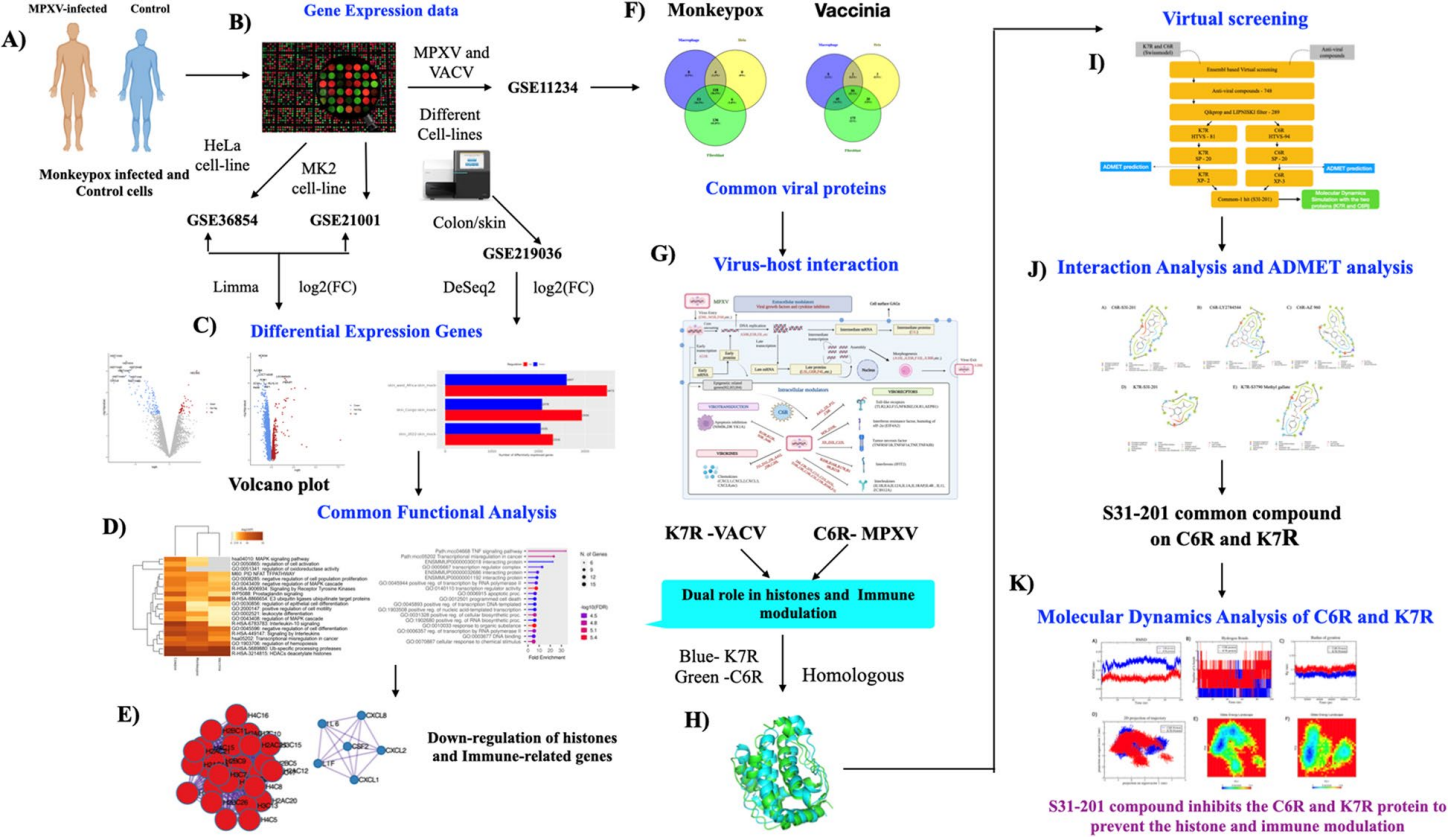
The schematic flowchart of the proposed study**.** Figure 1: **A**, **B** Collection of gene expression dataset on MPXV-infected and control (mock) samples in different cell lines. We collected the viral datasets on MPXV and VACV in different cells. These datasets were pre-processed for analysis **C** Differential Expression genes were identified in all datasets. **D** The functional analysis was performed on these DEGs. **E** MCODE analysis was performed, and two important clusters were down-regulated. **F** The common differential viral genes from MPXV and VACV were identified. **G** The overall virus-host interaction of identified differential viral genes was mentioned through this mechanism. **H** Through the functional analysis of viral genes, the C6R-derived protein K7 protein plays a dual role in histone and immune modulation. C6R-derived protein K7 and K7R are homologous. **I** The virtual screening was performed on the proteins and common compounds (S31-201) with the highest docking score. **J** The interaction analysis and ADMET analysis were performed. **K** Molecular Dynamics simulation of C6R-derived protein K7 and K7R with S31-201 compound was performed and analyzed

Given that poxviruses are known to inhibit cellular transcription for their advantage, the viral factors implicated in the dysregulation of histone expression and immune-evasion genes were explored. The investigation uncovered several significant regulatory downstream networks and probed the activities of gene clusters in the immune response. C6R-derived protein K7 was singled out as a potential target, and an antiviral drug was assessed using a virtual screening method. The virtual screening revealed that the chemical compound (S3I-201) shared by C6R-derived protein K7 from MPXV and K7R from VACV was due to homology. To confirm the stability of the protein molecule, Molecular Dynamics Simulations (MDS) were conducted on both protein–ligand complexes. The overall findings are summarized in Fig. 1 of the workflow.

## Methods

### Bioinformatics data acquisition

The four gene expression studies were retrieved from the GEO (Gene Expression Omnibus) database. Three datasets were derived from microarray investigations, and one was based on expression profiling by high-throughput sequencing (RNA-Seq). The datasets with virus-infected and control (mock) groups were included in this study. The first dataset is GSE36854, the host is *Homo sapiens,* and the cell line used is HeLa*,* which has been exposed for 6 h post-infection [10]. The second dataset is GSE21001, the cell line used is the Macaca mulatta kidney epithelial (MK2) cells that had been exposed to Mpox for 7 h post-infection [11]. The third dataset is GSE11234, the viral gene expression using three cell lines: primary human monocyte, primary human fibroblasts, and HeLa infected in MPXV and VACV [12]. The two viral transcriptomes display distinct temporal regulation and species-specific features of gene expression, and they offer basic knowledge of the overall gene expression responses to poxvirus infection. The fourth dataset is GSE219036 [13], and the host is *Homo sapiens*. Here, Watanabe et al. (2023) examined the effectiveness of viral growth in human keratinocytes and colon organoids produced from induced pluripotent stem cells and the host responses induced by MPXV infection. The detailed gene expression datasets are mentioned in Supplementary Table S1.

### Data pre-processing and identification of differential expression genes and viral proteins

The dataset's preliminary data were susceptible to background correction, quantile normalization, and log transition with robust multi-array technology [14]. As detailed by Alibés et al. (2007), the initial data processing involved converting individual gene symbols from probe IDs using Entrez's Gene ID converter [15]. The mean value of the observed gene contribution across many samples was calculated and considered the final gene expression level. The raw gene expression data were examined using the web statistical tool GEO2R, R/Bioconductor, and the Limma package v3.26.8 [16–18]. Using the built-in GEO2R methods, such as the T-test, the p-value and false discovery rate (FDR) were determined to determine the DEGs between patients with mock and infected groups [19]. For datasets GSE36854 and GSE11234, we set the primary requirements of | log (fold change) |≥ 2 and p ≤ 0.01 to get significant DEGs. In contrast, upregulated DEGs were considered if the logFC ≥ 2, and downregulated DEGs were considered if the logFC ≤ -2. On the other hand, for the Rhesus Monkey dataset, upregulated DEGs were taken into account if the logFC ≥ 1, while downregulated DEGs were taken into account if the logFC ≤ -1. The heat map and volcano plot were analyzed using the galaxy tool [20] and the Venn diagram using an online tool (https://bioinfogp.cnb.csic.es/tools/venny/) [21].

The raw count data were downloaded from the GEO database. Each sample with different Mpox clades (Colon organoids and keratinocytes) was taken separately and analyzed using the online tool IDEP [22]. The read counts data was then transformed using the EdgeR: log2 (CPM + c). The pre-processed data is then used for EDA, using heatmap and Principal Component Analysis (PCA) methods. The differential expression genes were analyzed using the DESeq2 method [23]. On the other hand, for the colon organoid sample type, P-value ≤ 0.05, upregulated DEGs were considered if the logFC ≥ 1. In contrast, downregulated DEGs were taken into account if the logFC ≤ -1, whereas, in the keratinocyte sample type, upregulated DEGs were taken into account if the logFC ≥ 2, while downregulated DEGs were taken into account if the logFC ≤ -2.

### Functional analysis of degs and viral proteins

Protein-level interaction analysis was carried out using the STRING Program [24]. Based on the FDR cutoff of 0.01, Metascape [25] was used to derive the gene ontology and pathway enrichment details for microarray studies (GSE36854 and GSE21001). The DEGs of RNA-Seq datasets were carried out using the IDEP tool. The in-build functional tools like GO-Biological Processes, GO-Molecular Functions, GO-cellular component, and KEGG pathway analysis [26] were analyzed for colon organoids and keratinocytes using the Biclustering method [27] to identify the different functional clusters among the subset of samples. The protein-level interaction network was obtained by loading the chosen DEGs from the GSE36854 and GSE21001 datasets into STRING using the multi-gene entry option. The Cluster feature in Cytoscape was used to find the cluster of interactions in the protein–protein interaction network [28]. The clustering method used in our study is MCODE (Molecular Complex Detection). The MCODE algorithm has been applied to identify densely connected network components. The functions of the viral proteins were retrieved from the UniProt database and literature-based studies [29–32]. The 3D structure was not available in UniProt or PDB database. The protein structure was predicted using Alphafold prediction and Visualized using PyMOL [33, 34]. The pair-wise sequence alignment of these two viral proteins was performed using an online tool (https://www.ebi.ac.uk/Tools/psa/).

### Virtual screening analysis

The hypothetical structures of K7R and C6R-derived protein K7 were constructed using SwissModel [35]. The protein structure was created using the Protein Preparation Wizard (Schrodinger), which included the addition of hydrogen atoms, the refinement of the loop region, optimization of the H-bond assignment, and finally, minimization of the constrained energy using an OPLS-2005 force field [36, 37]. The Glide-grid was produced using the Receptor Grid Generation module. To establish a new database, 748 compounds (Antiviral drugs) from an external database (https://www.selleckchem.com/) were processed through the LigPrep module. This module applied a force field (OPLS-2005z) [38], generated ionization states at pH 2.0, and created multiple conformers. The ligand molecules were also obtained in various states at pH 7.0 ± 2, using Epik version v5.3. High energy ionization/tautomer states were removed to increase the likelihood of reliability in the biological condition [39]. Prior to structure-based virtual screening, the antiviral compounds were screened using Lipinski's five rules with the Qikprop version 6.5 application [40]. Blind docking is performed for this protein. No water molecules remained in the protein, and no constraints, rotatable groups, or excluded volume were set. Three docking protocols were utilized in the Glide software and its virtual screening workflow process: high throughput virtual screening (HTVS), standard precision (SP) module, and extra precision (XP) module [41]. Each ligand was docked to the receptor using HTVS, resulting in a single pose. Approximately 50% of all compounds were advanced from HTVS to SP, even though the SP docking process offers a good scoring function that preserves the good scoring states [41]. This aids in identifying false-positive results. Furthermore, about 30% of all ligand molecules in SPs were advanced to XP, which offers the highest scoring states. XP provides the best scoring states. The detailed workflow of the virtual screening procedure is shown in Supplementary Figure S6.

#### ADMET

Comprehending pharmacokinetics, i.e., the behavior of a molecule in the organism, is vital for developing a new therapeutic drug. This is usually done based on individual indices known as ADMET characteristics (absorption, distribution, metabolism, exploitation, and toxicity). Instead of experimental methods, computer models are commonly used to ascertain these parameters. The compound's pharmacological and carcinogenic properties were assessed using the PkCSM web server [42].

### Molecular dynamics simulation

Molecular dynamics simulations (MDS) were carried out on the protein–ligand complexes C6R-derived protein K7-S3I-201 and K7R- S3I-201 utilizing Gromacs 5.0 software [43]. The GROMOS96 43a1 force field [44] was employed for these simulations. The initial structures for MDS were the three-dimensional structures of C6R-derived proteins K7 and K7R. The ligand's topology parameter files were created using the Swissparam online tool [45]. The protein structures were immersed in a cubic water box with a simple point charge (SPC) of 0.9 nm dimension. The system underwent neutralization via chloride ions while ensuring the particle count, pressure, and temperature remained unaltered. The Berendsen thermostat was used to keep the temperature constant, with a coupling time of 0.2 ps [46]. All atoms were kept at a minimum distance of one nanometer from the box edges. The system's energy was minimized using the steepest descent method. The molecular dynamics simulation was divided into three stages: heating, equilibration, and production. After an NPT ensemble was performed for 50,000 ps at 300 K, maintaining a constant number of particles, pressure, and temperature, an NVT ensemble was conducted at the same temperature, keeping the number of particles, volume, and temperature constant. This was followed by generating a molecular dynamics simulation trajectory for 100 ns at 300 K. The Linear Constraint Solver (LINCS) algorithm [47] was used to constrain the covalent bonds. The Particle Mesh Ewald (PME) method [48] was used to calculate electrostatic interactions. The cutoff radii for Van der Waals and Coulomb interactions were set to default values. The trajectory potentials from each Molecular Dynamics (MD) simulation were thoroughly analyzed using GROMACS tools [49].

Using the least squares method, the g_rms tool was used to calculate the root mean square deviation (RMSD) for a specific set of atoms in the protein molecule by fitting the protein molecule to the reference structure. The g_gyrate tool was used to measure the average distance of each atom in a molecule from its center of mass, indicating the compactness of the protein structure and providing insights into the stability of the complex. The g_hbond tool was used to identify the number of hydrogen bonds between two molecules and examine the potential for hydrogen bonds to form between potential donors (D) and acceptors (A).

Throughout the simulation period, the variations in total, potential, kinetic energies, pressure, and temperature were tracked as a function of simulation time to ascertain whether the systems adhere to constant NVT or NPT ensembles. The stability of the complex was explained by determining the number of hydrogen bonds formed and the minimal distance between protein–ligand complexes.

### Principal component analysis (PCA)

Essential dynamics involves the analysis of the principal motions of a biomolecule or a system of molecules, which are often crucial for the biological function of the molecule. PCA is frequently used to extract essential dynamics from MD trajectories, identifying major collective motions and understanding their significance [50, 51]. By reconstructing the configurational space using a simple linear transformation in Cartesian coordinate space, a 3N × 3N covariance matrix can be generated. The trajectory projection onto a specific eigenvector reveals the time-dependent motions of the components within that vibrational mode. The time-average of the projection illustrates how the constituent parts of atomic vibrations contribute to this coordinated motion mode. The Gibbs free energy landscape was computed using the gmx sham tool. The simulations were conducted for 100 ns, and the resulting plots were generated using XmGRACE Software.

## Results

### Screening of viral proteins from the Dataset of GSE11234

**Fig. 2:**
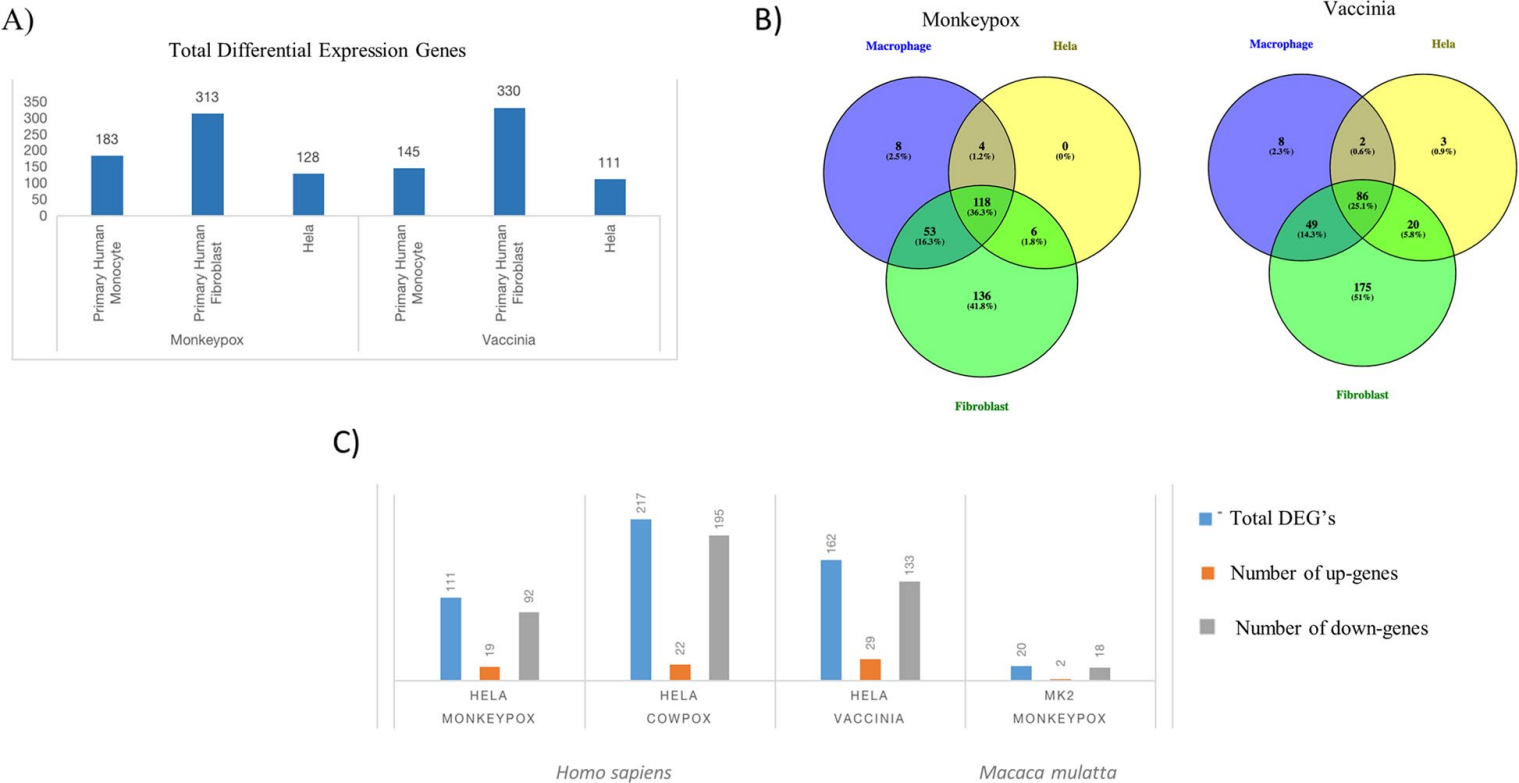
Statistical plot on Differential Expression Genes and overlap of viral proteins on the different cell types in MPXV and VACV. Figure 2: **A** The total number of differential viral proteins from the dataset GSE11234 in different cell types. **B** The differential viral proteins from different cell types were performed. MPXV and VACV yield 118 and 86 common viral proteins among these cell types, respectively. **C** The number of differential expression genes of up and down genes from the datasets GSE36854 (*Homo sapiens*) and GSE21001 (*Macaca mulatta*) were represented in the barplot

The expression patterns of viral proteins from MPXV and VACV were analyzed and compared across several cell types, including primary human monocyte, HeLa, and primary human fibroblast. Figure 2A illustrates the count of unique viral proteins in each cell type. Notably, primary human fibroblasts exhibit a higher number of differentially expressed genes compared to MPXV and VACV. Supplementary Table S3 provides a comprehensive list of viral proteins for different cell types infected with MPXV and VACV. Figure 2B emphasizes the overlap of viral proteins across various cell types during MPXV and VACV. Supplementary Table S4 details the overlap of differentially expressed viral proteins of MPXV and VACV across different cell types.

## Screening of DEGs from the Host (human and Rhesus Monkey).

**Fig. 3:**
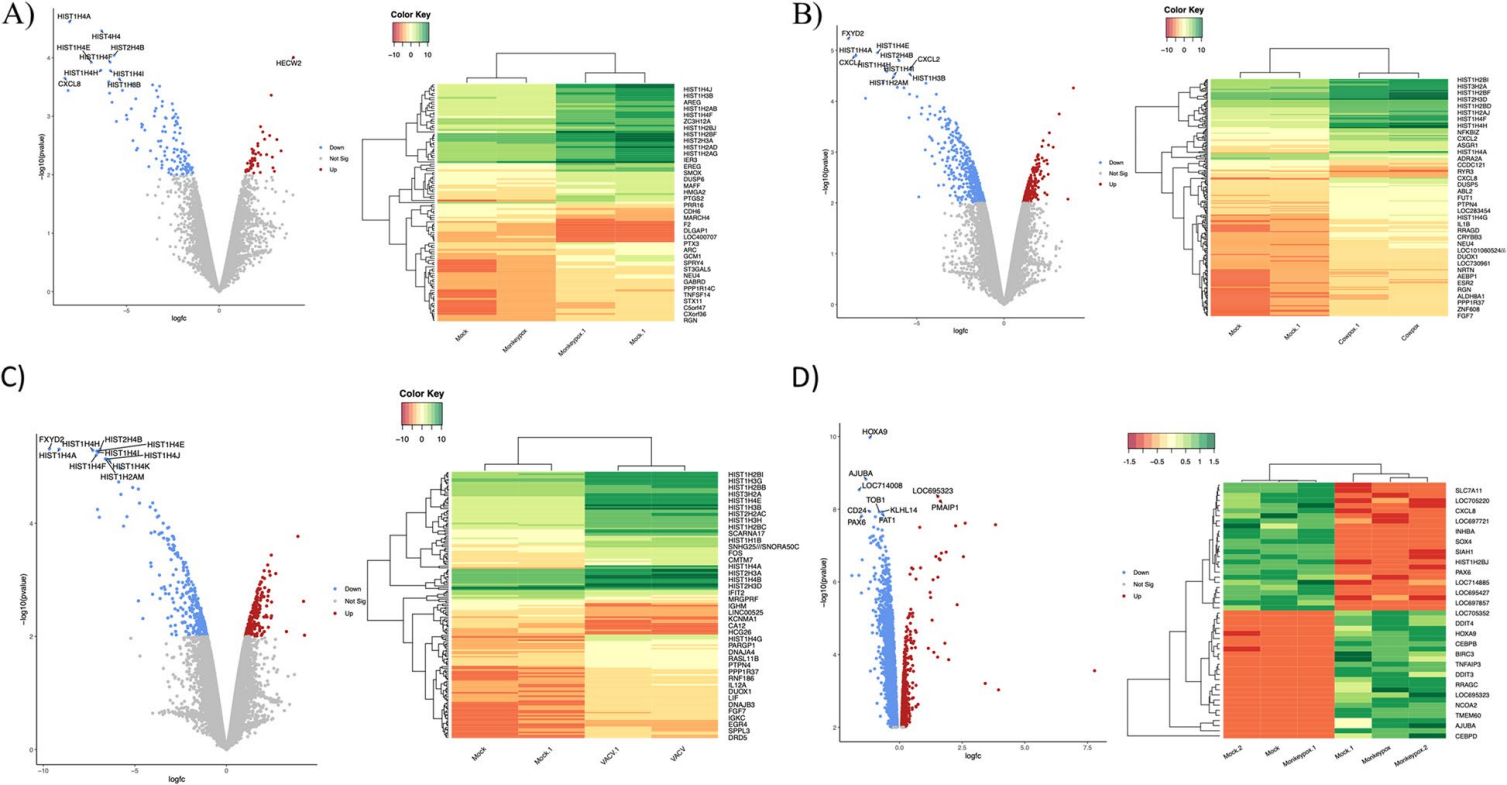
Volcano plot and heatmap of different pox infections in different hosts (GSE36854 and GSE21001). Figure-3: **A** The Volcano plot and heatmap of mock vs. MPXV-infected (*Homo sapiens*) are 19 upregulated and 92 down-regulated genes. **B** The Volcano plot and heatmap of mock vs. CPXV-infected (*Homo sapiens*) contains 22 upregulated and 195 down-regulated genes. **C** The Volcano plot and heatmap of mock vs. VACV-infected (*Homo sapiens*) shows 29 upregulated and 133 down-regulated genes. **D** In the Volcano plot and heatmap of mock vs. MPXV-infected (*Macaca mulatta*), there are 26 upregulated and 24 down-regulated genes. The top-most significant genes were mapped in the Volcano and heatmap in *Homo sapiens* and *Macaca mulatta*. The red color highlighted in the volcano plot is upregulated genes, and the blue color highlighted in the volcano plot is down-regulated.

**Fig. 4:**
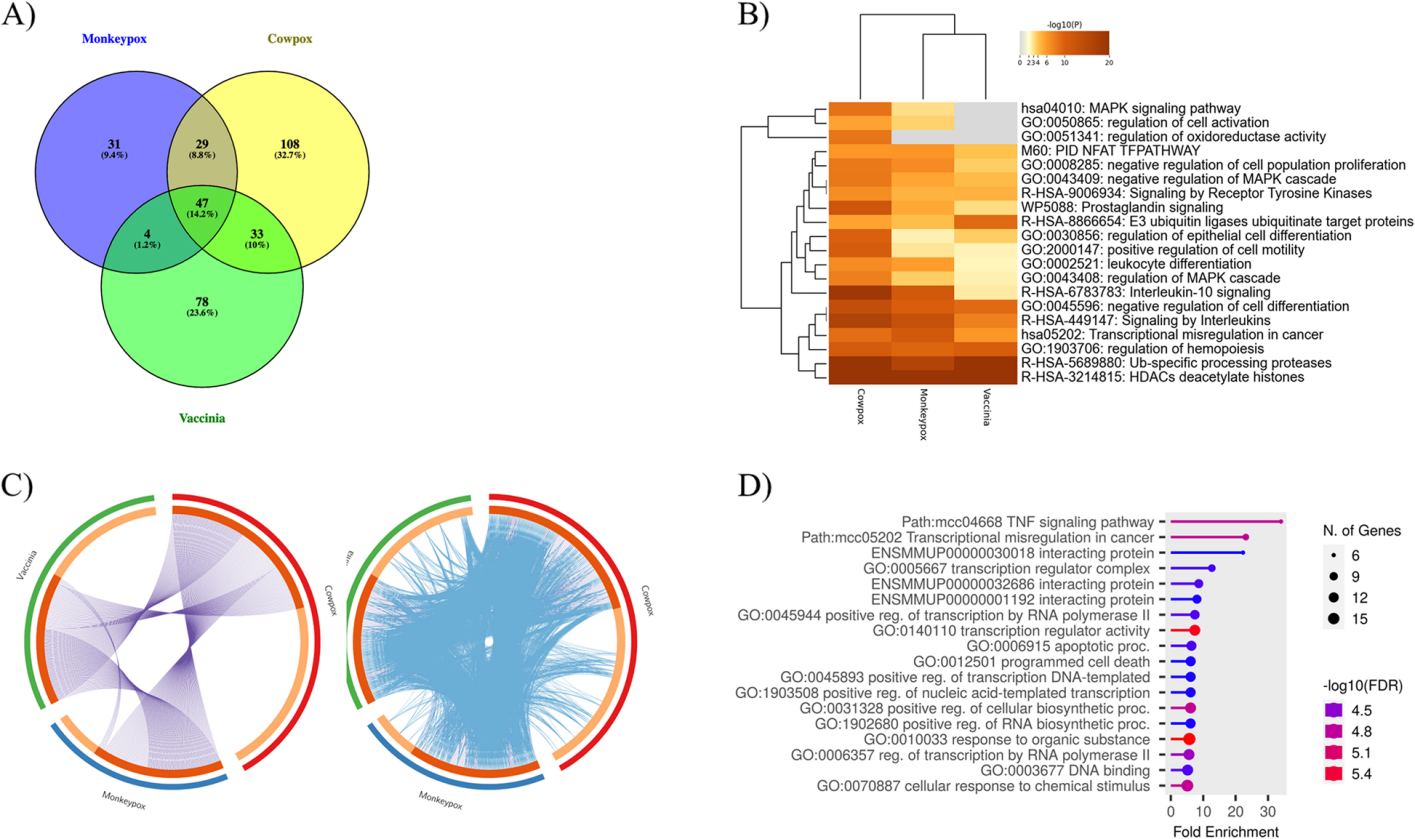
Overlap and functional enrichment analysis in different hosts (GSE36854 and GSE21001) Fig. 4-**A** The overlap of differentially expressed genes of different pox infections was depicted in the Venn diagram; 47 genes were overlapped. **B** The overlap functional enrichment was depicted in the heatmap, and important pathways and GO were enriched among all three pox infections. **C** The overlap of the input gene list among all three pox infections was depicted in the circos plot only at the gene level, where purple curves link identical genes. It includes the shared term level, where blue curves link genes that belong to the same enriched ontology term. The most enriched GO and KEGG pathways are transcriptional misregulation in Cancer, Interleukins, and MAPK signaling pathways. The inner circle represents gene lists, where hits are arranged along the arc. Genes that hit multiple lists are colored in dark orange, and genes unique to a list are shown in light orange **D** The functional enrichment of MPXV (*Macaca mulatta*) was depicted in the dot plot. The most enriched pathways are histone regulations and cytokine activity

To understand how poxvirus infection influences cellular transcription control, we initially examined the expression profiles of HeLa cells infected with MPXV, CPXV, VACV, and mock HeLa cells using the GSE36854 dataset. Figure 2C shows the differentially expressed genes (DEGs) linked to various pox infections. From the comparison between mock and MPXV-infected samples, we identified 111 DEGs, with 19 showing an increase in expression (upregulated) and 92 showing a decrease in expression (downregulated). When comparing mock and cowpox virus-infected samples, we found 217 DEGs, of which 22 were upregulated and 195 were downregulated. In the case of the mock and VACV-infected samples, there were 162 DEGs, with 29 genes showing increased expression and 133 genes showing decreased expression. The transcriptome data from the 7-h MPXV-infected Macaca mulatta kidney epithelial (MK2) cell from the GSE21001 dataset was compared with a mock and infected group to identify DEGs for the monkey cell line model. This dataset revealed 50 DEGs, with 26 upregulated and 24 downregulated. Supplementary Tables S2 and S3 provide a detailed list of differentially expressed genes of host and viral proteins. Figure 3 presents a Volcano plot and heat map showing the screened differential expression genes of these several pox infections from the human HeLa and MK2 cell lines. We utilized an additional dataset (GSE36854) to identify the genes exhibiting differential expression across three distinct pox infections. The genes that overlap among these infections are illustrated in a Venn diagram, revealing 47 genes common to these pox diseases (Fig. 4A). Figures 4B and 4D depict the gene ontologies and pathway analyses explored due to functional enrichment. Figure 4C, a circos plot, shows the number of genes associated with various pox infections. In these datasets, MPXV exhibits a higher propensity to infect keratinocytes, regulate cellular activation, inhibit the MAPK cascade, signal by interleukins, cause transcriptional misregulation in cancer, and deacetylate histones via HDACs. Similarly, the host organism, the Rhesus Monkey, shows comparable functional enrichment, such as the histone deacetylase family and TNF signaling pathway.

**Fig. 5:**
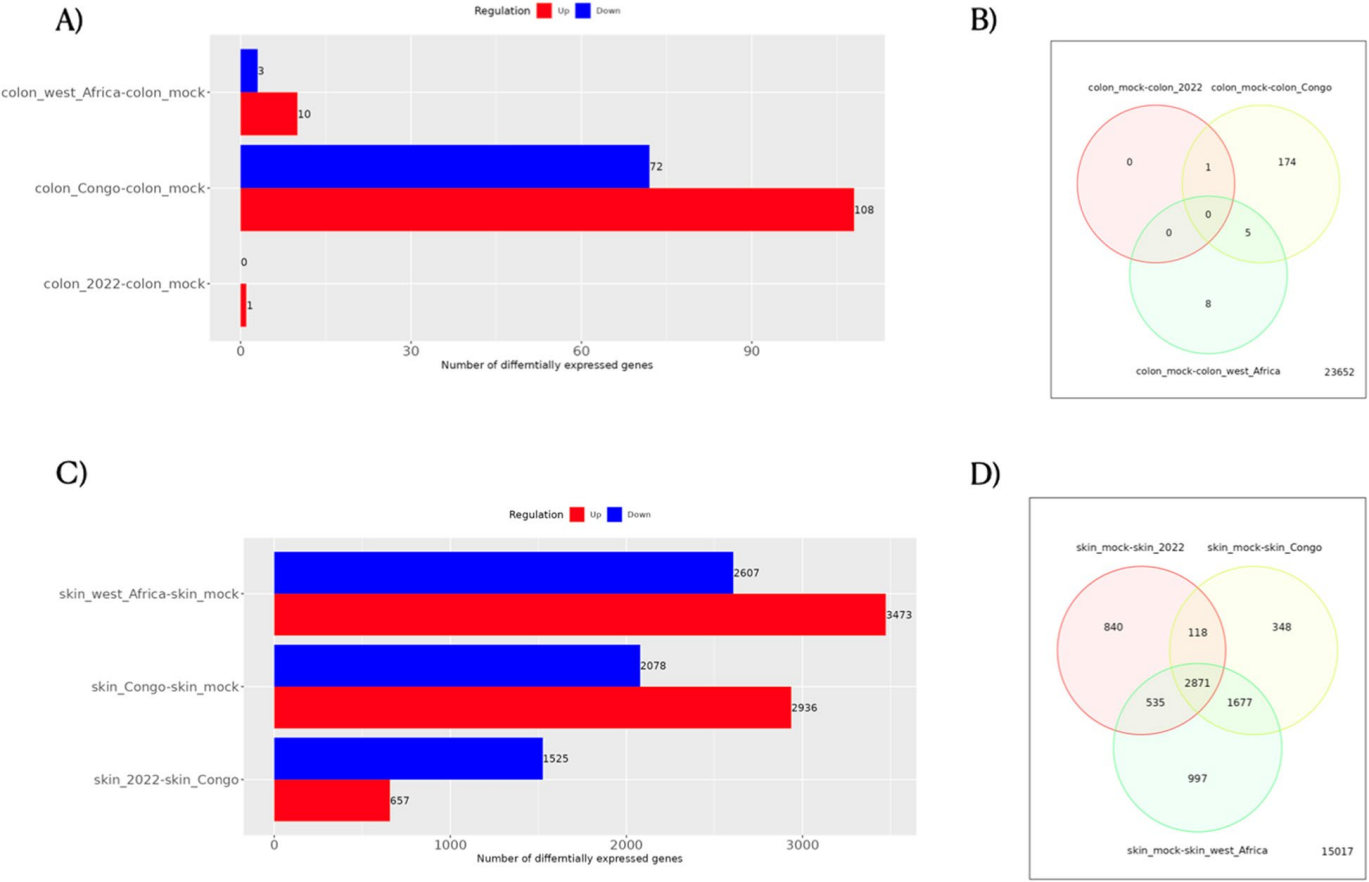
Statistical Analysis of GSE219036 of cell-type human colon organoids and keratinocytes. **A** The number of differential expression genes in different clades was represented in the barplot. The different factors of each clade are Colon_mock vs Colon_West_Africa (up-3;down-10), Colon_mock vs Colon_Congo (up-108;down-72), Colon_mock vs Colon_2022 (up-0;down-1). **B** The differential expression genes were compared in three Mpox strains and represented in the Venn diagram. There is no overlap in genes among these three different Mpox clades. The expression level is very low in human colon organoids. **C** The number of differential expression genes in different clades was represented in the barplot. The different factors of each clade are Skin_mock vs. Skin_West_Africa (up-2607;down-3473), Skin_mock vs. Skin_Congo (up-2078;down-2936), Skin_mock vs. Skin_2022 (up-2048;down-2316)). **D** The differential expression genes were compared in three Mpox clades and represented in the Venn diagram. There is a total of 2871 overlapped DEG in three different Mpox clades. All these figures were generated using the IDEP tool

Furthermore, we analyzed RNA-Seq datasets from various sample types infected with Mpox, along with a control(mock) group. The statistical plots for Colon organoids and human keratinocytes are provided in Supplementary Figures S1 and S2. Figure 5A presents the differential gene expression of human colon organoids across different Mpox clades. The barplot represents the number of differentially expressed genes in each clade. The factors for each clade are as follows: Colon_mock vs Colon_West_Africa (up-10; down-3), Colon_mock vs. Colon_Congo (up-108;down-72), Colon_mock vs. Colon_2022 (up-1;down-0). A Venn diagram (Fig. 5B) illustrates the comparison of these factors. The intracellular MPXV mRNA expression in infected colon organoids is minimal. Among these factors, Colon_mock vs. Colon_2022 and Colon_mock vs. colon_west_Africa did not achieve statistical significance, and the number of differentially expressed genes for these two factors was lower. Functional annotations were carried out using the biclustering method. The colon organoids have a single enriched cluster, while the human keratinocyte has three clusters.

Further functional annotations such as GO-BP were performed only for Colon_mock vs. Colon_Congo, as in Table 1. The enrichment of colon organoids in the Zr-599 strain (Clade-I) is primarily in "Cellular response to Zinc ion," "Response to Zinc ion," and other responses to the metal ion, with key functions highlighted. Figure 5C presents the differential gene expression of human keratinocytes across different Mpox clades, and a Venn diagram (Fig. 5D) illustrates the comparison of different factors (Skin_mock vs. Skin_2022; Skin_mock vs. Skin_Congo; Skin_mock vs. Skin_west_Africa). The comparison of DEGs between these factors yields 2871 genes.

**Table 1:** The functional annotation (GO-BP) of factor Colon_mock vs. Colon_Congo

GO-BP (Zr-599 MPXV)	Genes	adj.Pval
Detoxification of copper ion	11	3.00E-17
Stress response to copper ion	11	3.00E-17
Detoxification of inorganic compound	11	6.60E-16
^a^Cellular response to zinc ion	11	4.10E-14
^a^Cellular zinc ion homeostasis	13	5.30E-14
^a^Zinc ion homeostasis	13	9.20E-14
Cellular response to cadmium ion	13	1.10E-13
*Response to zinc ion	13	2.70E-12
Response to copper ion	12	5.80E-12
Cellular transition metal ion homeostasis	16	4.20E-11
Response to cadmium ion	13	6.30E-11
Transition metal ion homeostasis	16	4.30E-10
Detoxification	15	3.70E-09
Response to metal ion	20	0.00000043
Response to hormone	32	0.0000011

^a^Represents the important function in each strain in different clusters of different cell type

**Fig. 6:**
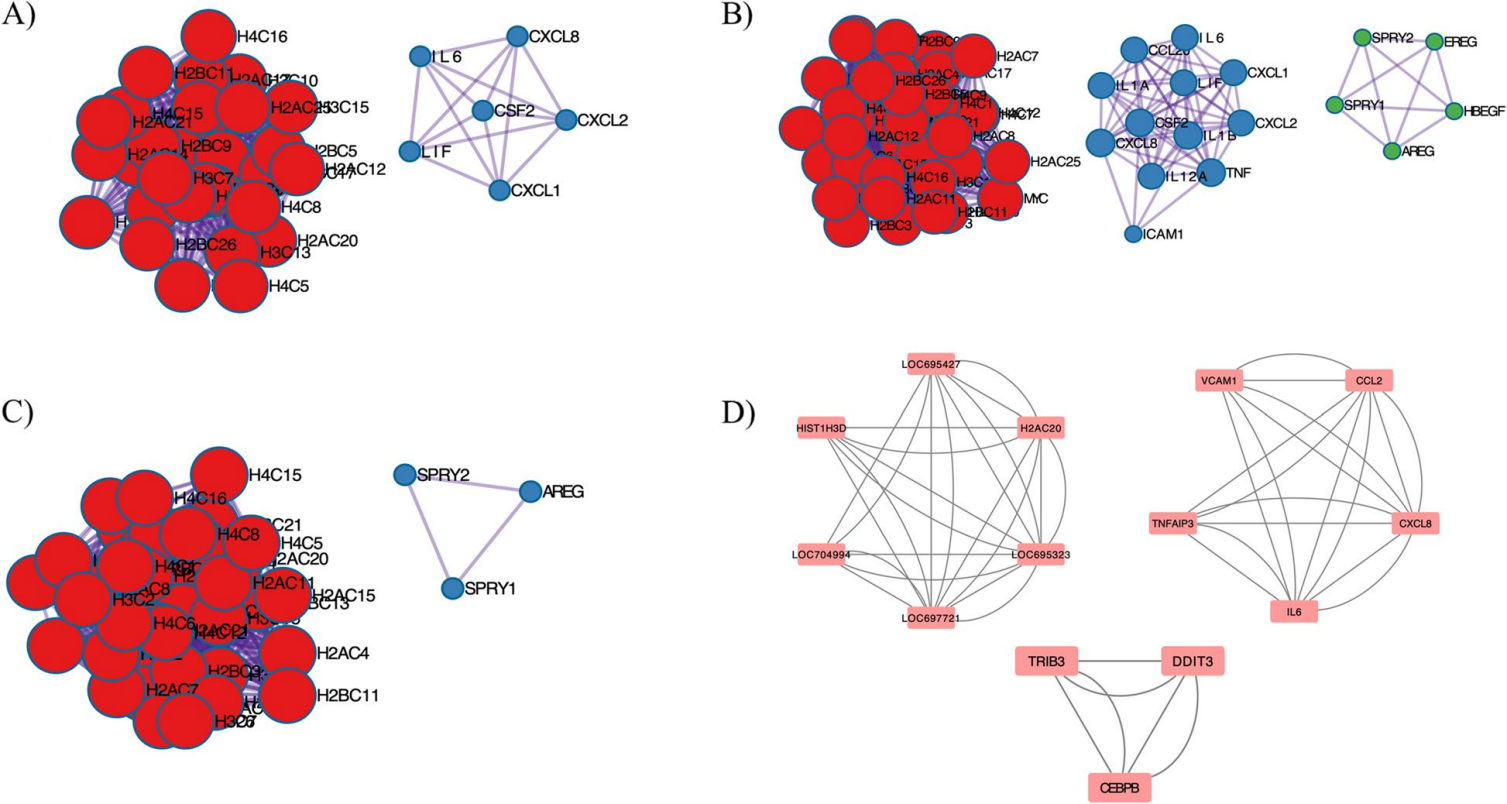
MCODE algorithm of different clusters in different pox infections. The MCODE algorithm separates the important genes in each PPI network with each cluster. **A** MPXV PPI clusters **B** CPXV PPI clusters **C** VACV Clusters. Red color represents the Histones, and the Blue color represents the Immune clusters. **D** This network was taken from the Rhesus Monkey PPI network and had two clusters. These figures were generated using the Metascape and Cytoscape tools

Functional pathways (KEGG) were annotated for all 12 samples, revealing three clusters. All three clusters contain important functional pathways related to Mpox infection, as detailed in Table 2. The "MAPK signaling pathway," "transcriptional misregulation in cancer," and other immune-related pathways are highly enriched in both datasets (GSE36854 and GSE219036). Other functional annotations, such as GO-BP, GO-CC, and GO-MF, were performed, and some of the key functions ("Keratinization" and "nucleosome assembly") were highlighted and mentioned in Tables 3, 4, and 5. Figure 6 provides details on the protein–protein interactions of several pox infections. CPXV and MPXV show a similar, stronger enrichment of host genes than VACV, with immunological and epigenetic mechanisms predominating. A new interaction between *BRCA1* and *EGR2* &*1* in MPXV*, MYC, SIRT6, FOS, EGR2* in VACV*, and MYC, CEBPA* in CPXV on histones and immune clusters was discovered via protein–protein interaction. The clusters from the PPI of various pox infections were studied and discussed in Supplementary Figure S3. Supplementary Figure S4 shows the combined network of many pox infections. Most frequently, these clusters were derived from histones and immune-related clusters.

**Table 2:** The functional annotation (KEGG) of all factors in human keratinocyte sample type

Cluster-1(KEGG)	Genes	adj.Pval
Systemic lupus erythematosus	28	6.6E-16
Alcoholism	31	3.7E-15
Neutrophil extracellular trap formation	31	3.9E-15
Viral carcinogenesis	25	1.9E-09
^a^Transcriptional misregulation in Cancer	24	2.7E-09
Legionellosis	10	0.000034
^a^IL-17 signaling pathway	10	0.002
^a^TNF signaling pathway	11	0.002
Circadian rhythm	6	0.002
^a^Cytokine-cytokine receptor interaction	19	0.0022
Shigellosis	17	0.0022
Viral protein interaction with cytokine and cytokine receptor	10	0.0024
Rheumatoid arthritis	9	0.006
**Cluster-2(KEGG)**	**Genes**	**adj.Pval**
Glutathione metabolism	5	0.0048
Metabolic pathways	27	0.0048
**Cluster-3(KEGG)**	**Genes**	**adj.Pval**
Alcoholism	14	1.7E-18
Systemic lupus erythematosus	13	1.7E-18
Neutrophil extracellular trap formation	13	9.2E-17
Viral carcinogenesis	7	0.00000065
Necroptosis	5	0.000075
Amphetamine addiction	3	0.0018
^a^IL-17 signaling pathway	3	0.0033
Rheumatoid arthritis	3	0.0033
Parathyroid hormone synthesis, secretion, and action	3	0.0043
^a^MAPK Signaling pathway	4	0.007
^a^Cytokine-cytokine receptor interaction	4	0.007
Breast cancer	3	0.0082

^a^Represents the important function in each strain in different clusters of different cell type

**Table 3:** The functional annotation (GO-BP) of all factors in human keratinocyte sample type

Cluster-1(GO-BP)	Genes	adj.Pval
^a^Response to organic substance	180	2.2E-17
^a^Nucleosome assembly	30	4.2E-15
Chromatin assembly or disassembly	33	1.4E-14
^a^Chromatin assembly	31	1.4E-14
^a^Nucleosome organization	32	7.5E-14
Cellular response to chemical stimulus	168	7.5E-14
Cellular response to organic substance	146	2.4E-13
Chromatin organization	53	4E-13
Chromatin remodeling	36	4.6E-13
DNA packaging	32	3.3E-12
RDNA heterochromatin assembly	15	4.2E-12
Regulation of hemopoiesis	44	5.1E-12
Nucleolar chromatin organization	15	5.4E-12
Negative regulation of macromolecule metabolic process	147	1.3E-11
Regulation of multicellular organismal process	138	1.3E-11
**Cluster-2 (GO-BP)**	**Genes**	**adj.Pval**
Cornification	28	4.6E-30
Epidermis development	40	2.6E-25
^a^Keratinocyte differentiation	34	7E-25
Skin development	37	4.5E-24
Epidermal cell differentiation	35	1.4E-23
^a^Keratinization	28	3.5E-21
Epithelial cell differentiation	40	3.9E-18
Tissue development	60	7E-17
Epithelium development	45	3E-14
Programmed cell death	51	2.1E-10
Cell death	53	2.3E-10
Peptide cross-linking	9	2.6E-09
Animal organ development	63	0.00000018
Cell differentiation	69	0.00000067
Cellular developmental process	69	0.0000015
**Cluster-3(GO-BP)**	**Genes**	**adj.Pval**
^a^Nucleosome assembly	9	3.5E-09
DNA replication-dependent nucleosome assembly	6	3.5E-09
Chromatin assembly	9	3.5E-09
RDNA heterochromatin assembly	6	5.8E-09
^a^Chromatin assembly or disassembly	9	5.8E-09
^a^Nucleosome organization	9	5.8E-09
Nucleolar chromatin organization	6	5.8E-09
Negative regulation of megakaryocyte differentiation	5	6.6E-09
Nucleolus organization	6	9.6E-09
DNA packaging	9	0.000000015
Megakaryocyte differentiation	7	0.000000033
Depurination	5	0.000000037
Regulation of androgen receptor signaling pathway	6	0.000000037
Amyloid fibril formation	7	0.000000037
Chromatin remodeling	9	0.000000048

^a^Represents the important function in each strain in different clusters of different cell type

**Table 4:** The functional annotation (GO-CC) of all factors in human keratinocyte sample type

Cluster-1(GO-CC)	Genes	adj.Pval
^a^Nucleosome	33	1.2E-23
^a^DNA packaging complex	33	6.6E-23
Protein-DNA complex	34	1.2E-15
Nucleoplasm	182	7E-11
^a^Chromatin	76	2.6E-10
Nuclear lumen	185	6.8E-09
Chromosome	86	0.0000019
Extracellular space	129	0.000094
Extracellular exosome	92	0.0001
Nuclear speck	30	0.00011
Extracellular organelle	92	0.00011
Extracellular vesicle	92	0.00011
Extracellular region	158	0.00016
Nuclear body	45	0.00032
I-kappa B/NF-kappaB complex	3	0.0011
**Cluster-2(GO-CC)**	**Genes**	**adj.Pval**
Extracellular space	88	1.6E-24
Extracellular region	98	4.7E-23
Extracellular organelle	68	4.7E-22
Extracellular exosome	68	4.7E-22
Extracellular vesicle	68	4.7E-22
Cornified envelope	15	4.1E-19
Vesicle	83	7.9E-16
^a^Intermediate filament	15	0.000000053
^a^Intermediate filament cytoskeleton	16	0.000000053
Desmosome	6	0.0000011
*Keratin filament	9	0.000018
Cell–cell junction	15	0.00014
Secretory granule	21	0.00023
Anchoring junction	20	0.00024
Ficolin-1-rich granule	9	0.00058
**Cluster-3(GO-CC)**	**Genes**	**adj.Pval**
^a^Nucleosome	14	3.7E-21
^a^DNA packaging complex	14	4.9E-21
Protein-DNA complex	15	2.3E-19
^a^Chromatin	17	2.9E-10
Chromosome	18	0.000000009
Extracellular region	26	0.000000012
Extracellular space	22	0.000000085
Chromosome, telomeric region	6	0.0000043
Extracellular organelle	16	0.0000043
Extracellular vesicle	16	0.0000043
Extracellular exosome	15	0.00002
Nuclear chromosome	6	0.000022
Chromosomal region	6	0.00019
Neurofibrillary tangle	2	0.00024
Vesicle	18	0.00068

^a^Represents the important function in each strain in different clusters of different cell type

**Table 5:** The functional annotation (GO-MF) of all human keratinocyte sample type factors

Cluster-1(GO-MF)	Genes	adj.Pval
^a^Protein heterodimerization activity	39	8.2E-12
DNA binding	120	0.000000012
^a^Protein dimerization activity	66	0.000000015
Nucleic acid binding	168	0.000000051
Protein domain-specific binding	42	0.000097
E-box binding	8	0.0014
Identical protein binding	86	0.0028
^a^Transcription factor binding	31	0.0046
^a^Transcription corepressor binding	4	0.0065
TRAIL binding	3	0.0065
Cadherin binding	21	0.0081
Oxidoreductase activity	38	0.0083
C3HC4-type RING finger domain binding	4	0.0097
**Cluster-2 (GO-MF)**	**Genes**	**adj.Pval**
Structural molecule activity	22	0.000016
^a^Structural constituent of skin epidermis	5	0.000016
Peptidase regulator activity	12	0.000032
Serine-type endopeptidase inhibitor activity	8	0.000056
Endopeptidase inhibitor activity	10	0.00012
Peptidase inhibitor activity	10	0.00013
Endopeptidase regulator activity	10	0.00016
Gap junction channel activity involved in cell communication by electrical coupling	3	0.00034
^a^Structural constituent of cytoskeleton	7	0.00055
RAGE receptor binding	3	0.00092
Molecular function regulator	33	0.0013
Signaling receptor binding	28	0.002
Interleukin-1 receptor antagonist activity	2	0.0024
Protease binding	7	0.0028
Fatty acid binding	4	0.0028
**Cluster-3(GO-MF)**	**Genes**	**adj.Pval**
^a^Protein heterodimerization activity	13	1.5E-12
^a^Protein dimerization activity	14	0.000000088
DNA binding	17	0.000015
Nucleic acid binding	19	0.00043
Signaling receptor activator activity	7	0.00043
Receptor ligand activity	7	0.00043
^a^Cytokine activity	4	0.0091
Unfolded protein binding	3	0.0095

^a^Represents the important function in each strain in different clusters of different cell type

### Integrative analysis of the influence of viral proteins on the Host system

**Fig. 7:**
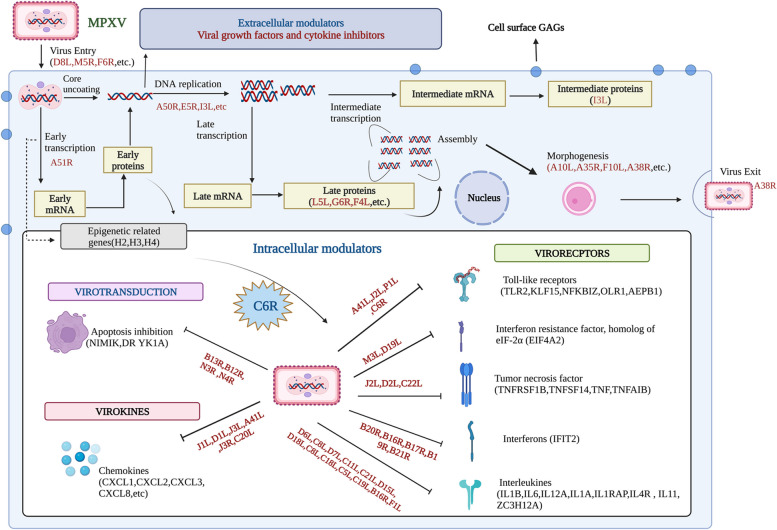
Integrative result of viral proteins and a Host system in MPXV infection. These results depicted the process of viral proteins that have impacted Host organisms (*Homo sapiens* and *Macaca mulatta*) with histones and immune modulation. This viral protein has certain functions that will impact both histones and the immune system from Mpox infection. These figures were generated using Biorender

The roles of the DEG-relevant viral proteins of MPXV and VACV were deduced through an analysis of the differential viral proteins in the UniProt database and studies based on literature. Supplementary Table S5 provides a detailed description of the functions of these viral proteins. These viral proteins were categorized into clusters and forms based on their functional similarities. Poxviruses evade the host immune system by producing viral proteins with diverse activities that influence key components of the inflammatory response. Through virotransducers, virokines, and viroreceptors, they specifically target mediators of innate and cell-mediated immune responses. One hundred eighteen viral proteins are common among different cell types; moreover, most of the viral-DEGs functionally align with the host-DEG profile, with a significant downregulation of host innate immune response proteins. The integrated results are presented in Fig. 7, and some crucial functions are listed in Table 6. The MPXV C6R-derived protein K7, similar to the VACV K7R, interacts with histones and selectively inhibits viral gene expression. The results are presented in Supplementary Figure S5.

**Table 6:** Categorization of immunomodulatory proteins

Functions	Category	Viral Proteins
Viral growth factor	Virokines	B3R, D3R
IL-18 binding protein	Virokines	D6L, C8L, D7L, C11L, C21L, D15L, D18L, C8L, C18L, C5L, C19L
Complement binding protein	Virokines	D12L, C17L
Interferon resistancefactor, homolog of eIF-2α	Viroreceptors	M3L, D19L
Interferon resistance factor, dsRNA-binding protein	Viroreceptors	F3L
3-β -Hydroxy-delta 5-steroid dehydrogenase	Virotransduction	A45L
Interferon-ɣ binding protein	Viroreceptors	B9R, B7R, B6R
Serine protease inhibitor homolog, SPI-2, inhibition of IL-1 β converting enzyme, apoptosis inhibition	Virotransduction	B13R, B12R, N3R, N4R
Interleukin-1 β-binding protein	Viroreceptors	B16R, F1L, C6R derived protein K7
Interferon- α/β -binding protein	Viroreceptors	B20R, B16R, B17R, B19R, B21R
Serine protease inhibitor homolog, SPI-1, apoptosis inhibition	Virotransduction	B19R, B20R, C12L, B13R
Tumor necrosis factor binding protein	Viroreceptors	J2L, D2L, C22L
Chemokine binding protein	Virokines	J1L, D1L, J3L, A41L, J3R, C20L
Semaphorin-like	Virokines	A42R, A39R
NF-kappa B cascade	Viroreceptors	A46R, B4R, P1L, C4L, C7L, B2R, A49R
Phosphatases	Virotransduction	C10L, I1L, C2L, D4L

### Virtual screening results

**Fig. 8:**
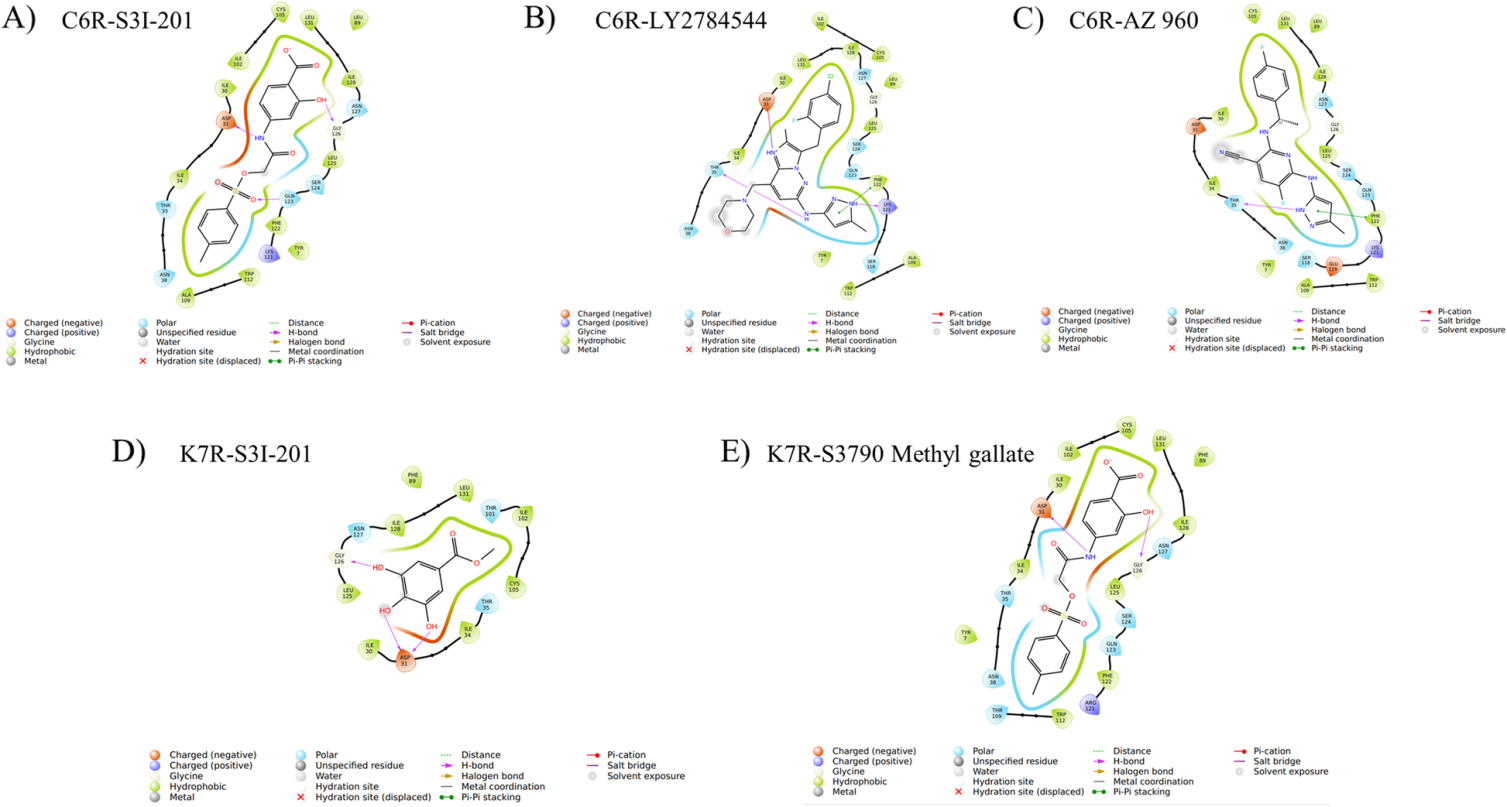
Two-dimensional structural depiction of the molecular interactions within the complexes: **A** S3I-201 with C6R-derived protein K7, **B** LY2784544 with C6R-derived protein K7, **C** AZ 960 with C6R-derived protein K7, **D** S3I-201 with K7R, and **E** S3790 Methyl gallate with K7R

The outcome of the virtual screening process identified a common compound that could potentially act as an inhibitor due to its non-covalent interactions with the viral proteins C6R derived protein K7 and K7R. Initially, a total number of antiviral compounds were screened using the Qikprop and Lipinski filtering module, passing 289 compounds. The high throughput virtual screening module further filtered these to 94 and 81 hits for the C6R-derived protein K7 and K7R proteins, respectively. A subsequent filtering process using the standard precision (SP) program further reduced the hits to 20 for both proteins. The extra precision (XP) program was then employed for more accurate screening, resulting in the final selection of 3 compounds for C6R-derived protein K7 (MPXV) and 2 for K7R (VACV). The compounds of C6R-derived protein K7 are S31-201, LY2784544, and AZ 960, and the compounds of K7R are S31-201 and S3790 Methyl gallate. The interactions of the compounds are depicted in Fig. 8, and the detailed results of the virtual screening are provided in Table 7. The common compound S31-201 exhibited the highest docking score among all compounds in both proteins and was further subjected to Molecular Dynamics simulation (MDS).

**Table 7:** Estimation of the docking scores outcome (expressed in kcal/mol) and the calculation of binding affinity (also in kcal/mol) for the highest-ranking compounds

C6R-derived protein K7_Protein							
**Compound_name**	**SMILES**	**r_glide_XP_GScore**		**No of h-bonds**			**Interacting_residues**
S31-201	Cc1ccc(S(= O)(= O)OCC(= O)Nc2ccc(C(= O)[O-])c(O)c2)cc1		-6.58447	3			ASP31;GLY126;GLN123
LY2784544	Cc1cc(Nc2cc(CN3CCOCC3)c3nc(C)c(Cc4ccc(Cl)cc4F)n3n2)[nH]n1		-6.27182	2			THR35;LYS121
AZ 960	Cc1cc(Nc2nc(N[C@@H](C)c3ccc(F)cc3)c(C#N)cc2F)n[nH]1		-6.4269	1			THR35
**K7R protein**							
S31-201	Cc1ccc(S(= O)(= O)OCC(= O)Nc2ccc(C(= O)[O-])c(O)c2)cc1		-6.29787		2	ASP31;GLY126	
S3790 Methyl gallate	COC(= O)c1cc(O)c(O)c(O)c1		-6.04289		3	ASP31(2);GLY126	

#### ADMET

To ensure the safety and efficacy of the identified molecules, it is crucial to evaluate the pharmacokinetics and toxicity characteristics of the ligand. The lead compound was examined for CYP inhibition, hepatotoxicity, carcinogenicity, absorption across the blood–brain barrier, p-glycoprotein inhibition, and CNS permeability. CNS permeability, which determines the ability to cross the blood–brain barrier, is considered to permeate the central nervous system when CNS >  − 2. None of the three compounds exhibited any carcinogenic or toxicity profiles in the carcinogenicity and AMES toxicity assessments. According to the Lipinski rule of five, which investigates the number of hydrogen bond donors, acceptors, and the surface area of the ligand molecules, the selected compounds demonstrated a favorable response. The detailed results of the ADMET analysis can be found in Table 8.

**Table 8:** Pharmacological characteristics of the leading ligand molecules for K7R and C6R-derived protein K7, obtained from the pKCSM webserver

Property	Parameter	S31-201	LY2784544	AZ 960	S3790 Methyl gallate
Molecular properties	Molecular Weight	364.355	469.952	354.364	184.147
Molecular properties	LogP	0.40812	4.02834	4.1797	0.59
Molecular properties	#Rotatable Bonds	6	6	5	1
Molecular properties	#Acceptors	7	7	5	5
Molecular properties	#Donors	2	2	3	3
Molecular properties	Surface Area	143.358	195.275	148.446	73.819
Absorption	Water solubility	-4.095	-2.967	-3.264	-1.47
Absorption	Caco2 permeability	0.837	1.051	0.699	0.925
Absorption	Intestinal absorption (human)	40.273	92.903	93.141	61.796
Absorption	Skin Permeability	-2.733	-2.735	-2.746	-2.777
Absorption	P-glycoprotein substrate	Yes	Yes	Yes	No
Absorption	P-glycoprotein I inhibitor	No	Yes	No	No
Absorption	P-glycoprotein II inhibitor	No	No	No	No
Distribution	VDss (human)	-1.218	1.075	0.652	-0.143
Distribution	Fraction unbound (human)	0.161	0.234	0.208	0.38
Distribution	BBB permeability	-0.714	-1.673	-1.306	-1.03
Distribution	CNS permeability	-3.558	-2.616	-2.352	-4.106
Metabolism	CYP2D6 substrate	No	No	No	No
Metabolism	CYP3A4 substrate	No	No	No	No
Metabolism	CYP1A2 inhibitor	No	No	Yes	No
Metabolism	CYP2C19 inhibitor	No	Yes	Yes	No
Metabolism	CYP2C9 inhibitor	No	Yes	Yes	No
Metabolism	CYP2D6 inhibitor	No	No	No	No
Metabolism	CYP3A4 inhibitor	No	Yes	No	No
Excretion	Total Clearance	0.239	0.842	-0.184	0.693
Excretion	Renal OCT2 substrate	No	Yes	No	No
Toxicity	AMES toxicity	No	Yes	No	No
Toxicity	Max. tolerated dose (human)	0.959	0.61	0.223	0.696
Toxicity	hERG I inhibitor	No	No	No	No
Toxicity	hERG II inhibitor	No	Yes	No	No
Toxicity	Oral Rat Acute Toxicity (LD50)	2.172	2.422	2.697	2.009
Toxicity	Oral Rat Chronic Toxicity (LOAEL)	2.371	2.282	1.59	2.917
Toxicity	Hepatotoxicity	No	Yes	No	No
Toxicity	Skin Sensitisation	No	No	No	No
Toxicity	T.Pyriformis toxicity	0.283	0.285	0.333	0.207
Toxicity	Minnow toxicity	-0.422	3.669	3.204	1.925

### Molecular Dynamics simulation results

MD is a computational method for predicting the time-dependent motion of an atomic system by solving Newton's equations of motion [52]. We performed MDS at 100 ns of the C6R-derived protein K7-S3I-201 and K7R-S3I-201 complexes to evaluate the protein's structural stability. The following structural parameters were analyzed from the MD trajectories: Root Mean Square Deviation (RMSD), Radius of gyration (Rg), Hydrogen bond, Essential Dynamics (ED), and Gibbs free energy landscape. RMSD is a metric used in MDS to assess the structural stability and conformational changes over time. The RMSD of the protein backbone was computed throughout the simulation period to ensure structural stability. In MDS, RMSD is frequently employed to quantify the spatial disparities between an initial structure and its subsequently estimated coordinates over time [53]. Throughout the simulation, this parameter can assess the structural convergence of protein structures and analyze their time-dependent motion. The C6R-derived protein K7-S3I-201 and K7R-S3I-201 exhibited RMSD values of 0.2 and 0.1 nm, respectively, suggesting that the protein structure maintains its overall conformation. In the initial stages of a simulation, RMSD may exhibit fluctuations when the system is in equilibrium. Once equilibrium is reached, the RMSD typically reaches a plateau, indicating that the system has settled into a stable conformation.

In MDS, hydrogen bonds are crucial for stabilizing protein structures and mediating molecular interactions [54]. Hydrogen bonds contribute to the stability of protein secondary structures. A crucial step in molecular recognition includes interaction specificity and directionality. The number of hydrogen bonds formed in the C6R-derived protein K7-S3I-201 complex is five and six in the K7R-S3I-201 complex. Both the complexes show good binding between protein and ligand. The persistence of these hydrogen bonds throughout the simulation suggests that these complexes are relatively stable interactions.

Rg is a measure of the compactness or spread of a biomolecular structure. Rg is calculated as the root mean square distance of a collection of atoms from their common center of mass [55]. It quantifies the overall size and shape of the biomolecule in the simulation. The C6R-derived protein K7-S3I-201 and K7R-S3I-201 complexes exhibited a value of 1.5 nm. A smaller Rg value indicates a more compact structure where the atoms are closer to the center of mass. This may correspond to a folded or tightly packed protein. These results indicate that the two protein complexes are stable and relatively compact structures.

The MDS employs ED or PCA to examine the fundamental movements of biomolecular systems. The MD trajectories of C6R-derived protein K7-S3I-201 and K7R-S3I-201 complexes were projected into the subspace spanned by PC1 and PC2. According to the ED analysis, the dominating motions are captured by the first two principal components (PC1 and PC2). In the case of the C6R-derived protein K7-S3I-201 complex, the PCs are between -1.8 and 3.3 on PC1 and -2 and 1.5 on PC2, while the motion in the K7R-S3I-201 complex is between 1.4 and 2.9 on PC1 and -1.5 to 1.9 on PC2. Both complexes displayed more variable conformation and occupied a larger area in the conformational space. Modifications to the cluster's shape were also noted in the conformational space in all complexes.

**Fig. 9:**
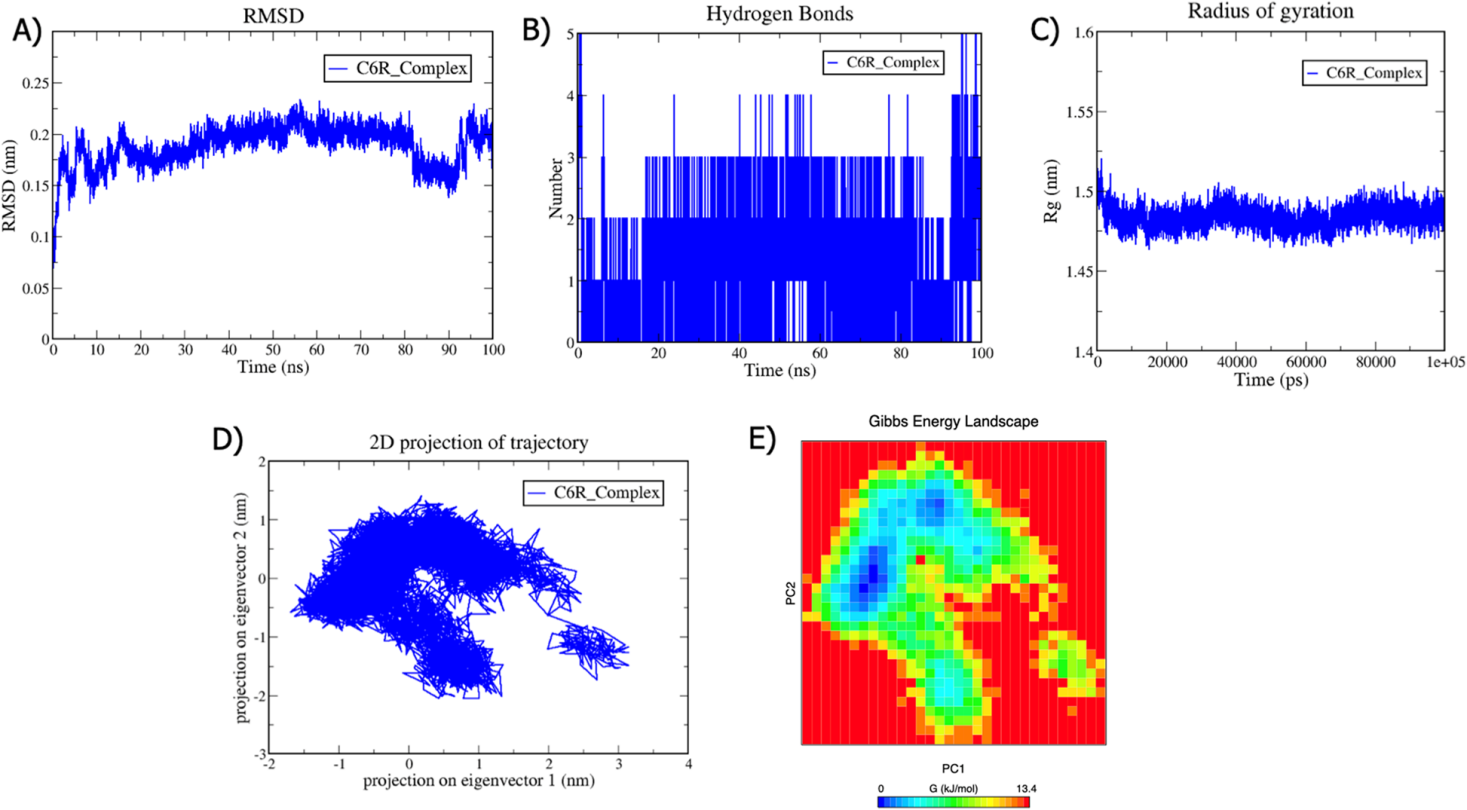
Analysis of Molecular Dynamics Simulation (100 ns) for the C6R-derived protein K7-S3I-201 complex (depicted in blue). **A** The Root Mean Square Deviation (RMSD) values, computed for the backbone atoms at a temperature of 300 K, are plotted over time. The X-axis denotes time in nanoseconds (ns), while the Y-axis signifies RMSD in nanometers (nm). **B** The graph illustrates the count of hydrogen bond interactions. The X-axis denotes time in nanoseconds (ns), and the Y-axis signifies the quantity of hydrogen bonds. **C** The plot of the Radius of Gyration (Rg) is presented. The X-axis denotes time in picoseconds (ps), and the Y-axis signifies Rg in nanometers (nm). **D** In the Principal Component Analysis, the 2D projections of trajectories on the first two eigenvectors are exhibited. The X-axis denotes the projection on eigenvector 1 in nanometers (nm), and the Y-axis signifies the projection on eigenvector 2 in nanometers (nm). E) The Gibbs energy landscape is depicted. The X-axis denotes PC1 in nanometers (nm), and the Y-axis signifies PC2 in nanometers (nm). The Gibbs free energy is expressed in units of kilojoules per mole (KJ/mol)

**Fig. 10:**
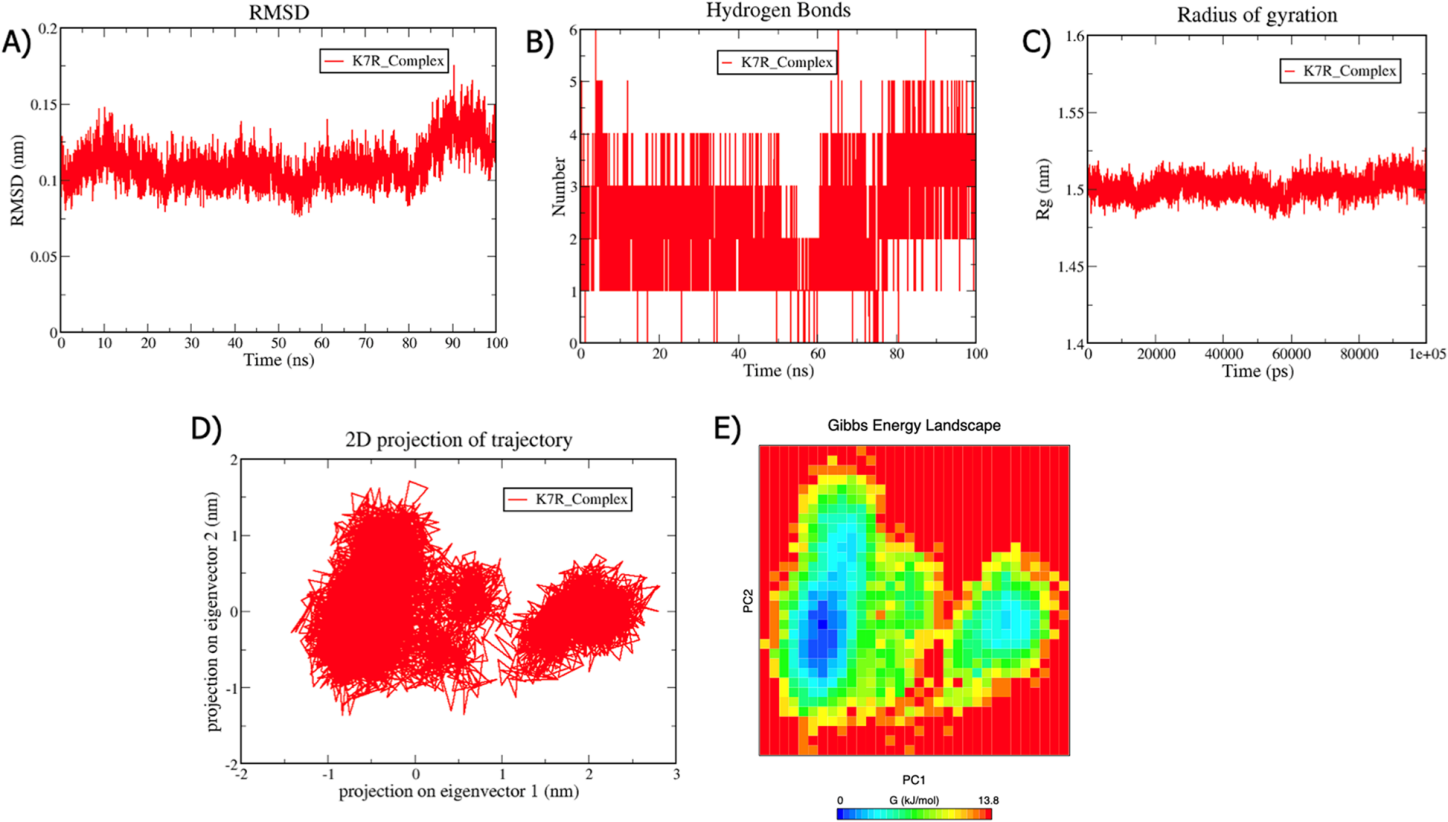
Analysis of Molecular Dynamics Simulation (100 ns) for the K7R-S3I-201 complex (depicted in red). **A** The Root Mean Square Deviation (RMSD) values, computed for the backbone atoms at a temperature of 300 K, are plotted over time. The X-axis denotes time in nanoseconds (ns), while the Y-axis signifies RMSD in nanometers (nm). **B** The graph illustrates the count of hydrogen bond interactions. The X-axis denotes time in nanoseconds (ns), and the Y-axis signifies the quantity of hydrogen bonds. **C** The plot of the Radius of Gyration (Rg) is presented. The X-axis denotes time in picoseconds (ps), and the Y-axis signifies Rg in nanometers (nm). **D** In the Principal Component Analysis, the 2D projections of trajectories on the first two eigenvectors are exhibited. The X-axis denotes the projection on eigenvector 1 in nanometers (nm), and the Y-axis signifies the projection on eigenvector 2 in nanometers (nm). **E** The Gibbs energy landscape is depicted. The X-axis denotes PC1 in nanometers (nm), and the Y-axis signifies PC2 in nanometers (nm). The Gibbs free energy is expressed in units of kilojoules per mole (KJ/mol)

The Gibbs free energy landscape was projected using the first two principal components, PC1 and PC2. These results provide insight into the energetics and stability of different states or transitions within the biomolecular system. The color-coded representation of the Gibbs free energy landscape for all the systems was shown. The color bar displays, from the lowest to the highest, the Gibbs free energies in KJ/mol for each structural state. The direction of the fluctuation for all Cα atoms was inspected for both complexes. The Gibbs free energy for C6R-derived protein K7-S3I-201 and K7R-S3I-201 are 13.4 kJ/mol and 13.8 kJ/mol, respectively. Blue shows a stable cluster and a larger region of various conformational states with lower energy minima. It occupies a wider region in both complexes and signifies a stable structure. Our comprehensive MDS study revealed that two protein–ligand complexes exhibit stability. The detailed MDS results of the two complexes are shown in Figs. 9 and 10.

## Discussion

Understanding the interactions between viruses and their hosts at the cellular and systemic levels is essential for determining the mechanisms of viral pathogenesis [56]. By employing differential gene expression profiling to thoroughly comprehend these interactions, we can identify key viral and host determinants that significantly influence the progression and outcome of the disease. We retrieved three different transcriptomics datasets from hosts to identify the DEGs in response to infection. To explore the impact of natural and accidental hosts on the pathogenesis and outcome, we conducted DEGs profiling in *Homo sapiens* and *Macaca mulatta*. In human HeLa cells, a significant number of host genes were expressed upon infection with MPXV. However, in MK2 cells, fewer DEGs than humans suggested a natural adaptation of Mpox. Unlike other viruses, poxviruses employ stealth strategies to undermine and evade the host's antiviral mechanisms [30], which reaffirmed study findings that the number of down-regulated host genes significantly outnumbers the upregulated genes. We attempted enrichment analysis to outline the functional attributes of the host's DEGs, which unveiled marked differences in DEGs among the analyzed poxviruses. All three studied poxviruses had similar enrichment of host genes with a predominance of immune and epigenetic factors across hosts. The common pathways, such as transcriptional misregulation in cancer, MAPK signaling pathways, and cytokine-cytokine receptors, were enriched in all three transcriptomics studies.

The protein–protein interactions (PPIs) across all three viruses reveal strong clusters of genes associated with histones and immune functions. These PPIs uncover several novel interactions that could potentially influence the pathogenesis of MPXV, VACV, and CPXV. Specifically, MPXV upregulates the expression of BRCA1, a protein that interacts with various components of the histone deacetylase complex and regulates transcription [57]. Conversely, *EGR1* & *2*, multifunctional mammalian transcription factors, are down-regulated by MPXV. These factors modulate the expression of growth factors, cytokines, and apoptosis [58, 59]. Their involvement in the replication and pathogenesis of RNA and DNA viruses is well-documented. The robust cumulative interaction of BRCA1 and EGR1 with histones may influence the establishing of a cellular antiviral state or pro-cellular molecular events, thereby affecting the overall pathogenesis of MPXV infection [60].In contrast, VACV exhibits a broader down-regulation of proteins (*MYC*, *SIRT6*, *FOS*, *EGR2*) interacting with histones. Except for *SIRT6*, a transcription corepressor [61], *MYC*, *FOS*, and *EGR2* are transcription factors. Most of these proteins are involved in either transcription or regulation. The convergence of this common functionality suggests that poxviruses strategically exploit histones and control cellular transcription for their benefit. This reaffirms our findings, which showed a strong enrichment of histones (H2, H3, H4) with pronounced down-regulation affecting both viral and cellular transcriptions.

The viral gene expression datasets of MPXV and VACV were used to identify the significant viral genes. As expected, the data indicated that the virulent MPXV had higher DEGs than the innocuous well, adapted VACV. Interestingly, the oncogenic HeLa cell did not exhibit any specific viral gene expression compared to immune and non-immune cell types. Poxviruses are known to evade the host immune system by synthesizing viral proteins with versatile functions that impact the critical components of the inflammatory response [62]. They specifically target innate and cell-mediated immune response mediators through viromimicry, virokines, and viroreceptors [30, 63]. Our data corroborate this, as many of the 118 overlapping viral proteins of MPXV are known to exert virostealth, virotransduction, and viromimicry.

Additionally, the viral DEGs were mainly interacting with chemokine, complement, TNF, IL-18 &1, and interferons. These functionally matched the host-DEG profiles with the profound down-regulation of proteins involved in the host's innate immune response, immune signaling, proteasome functions, apoptosis, and cell differentiation. Notably, the MPXV C6R-derived protein K7, similar to the Vaccinia K7R, is known to bind with histones and selectively suppress viral gene expression [64, 65].

The re-emergence of Mpox can occur due to its broad ecological niche, animal reservoir, and lack of vaccines. Thus, therapeutic interventions for Mpox could be a viable alternative to the impracticality of rapid mass immunization [66]. Currently, brincidofovir and tecovirimat are the only available treatments [67]. However, their efficacy is not widely evaluated in the global population. Hence, it is important to expand the therapeutics approaches to other Mpox targets. Few studies have repurposed existing drugs for Mpox in different targets such as p37, A20R, A48R, A50R, D13L, F13L, I7L, and VETFS [68–71]. The targeted viral proteins are crucial in viral replication. However, the present study focuses on identifying the potential drug target against C6R-derived protein K7 because of its dual role in modulating the epigenetics and the immune response. Gene expression analysis in this study revealed down-regulation of histones and immune genes in humans and other model species. The C6R-derived protein K7, a homolog of the K7R protein, emerged as a potential target. The model structure underwent molecular docking against the selected library, revealed S3I-201 had the highest binding energy and interactions with C6R-derived protein K7 and K7R proteins.S3I-201 (NSC74859) is a small molecule inhibitor specifically targeting the SH2 domain of STAT3, disrupting its dimerization and subsequent activation [72, 73]. STAT3 is a transcription factor involved in numerous cellular processes, including cell proliferation, survival, and immune responses [74]. Furthermore, the MPXV C6R-derived protein K7 can inhibit the activation of the NFκB pathway and IRF3 [75, 76]. The other Mpox protein, D11L, inhibits the STAT signaling pathway and the activation of IRF3 and IRF7 [75–78]. We believe S3I-201 binding to C6R-derived protein K7 will not prevent the replication of the virus, but it will ameliorate the virus-mediated perturbation of host defense response. However, it needs careful experimental validation.

MDS was utilized to evaluate the stability of protein–ligand complexes for 100 ns. In MDS, structural parameters refer to geometric and spatial characteristics of biomolecular systems that describe the conformational states. These parameters are often monitored and analyzed throughout the simulation to understand the structural dynamics and behavior of the system [79]. The structural parameters used in this study include RMSD, Rg, Hydrogen bond, PCA, and Gibbs free energy landscape. The C6R-derived protein K7 and K7R complexes exhibited low RMSD values of 0.2 nm and 0.1 nm. It is apparent in the literature that an RMSD value ≤ 0.2 nm is fairly good. A low RMSD value suggests that the overall protein structure is similar to the reference structure [80]. A small RMSD value indicates that the protein has maintained its structural integrity and the system is stable throughout the simulation [81].In MDS, Rg indicates the compactness or spread of a protein structure [55]. Rg provides a measure of the overall size of the protein structure. In both the complexes, the Rg value is 1.5 nm, suggesting that the protein structure occupies a region of space. A relatively constant Rg value suggests that the structure remains stable and relatively compact structure [82]. Hydrogen bonding is essential for maintaining the structural stability of proteins in MDS. In both, the complexes exhibited a good number of interactions. The larger the number of h-bonds formed, the higher the binding affinity [83, 84]. The detection of many hydrogen bonds in MDS suggests the presence of stable and specific interactions within the biomolecular system, offering valuable insights into its structural and dynamic properties [85]. The protein motions were examined through PCA analysis. Both complexes occupied a larger space, indicating that more atoms are involved in coordinated movements throughout the simulation. Overall, the identification of modes that occupy larger spaces in ED analysis provides insights into structural dynamics and flexibility [86]. A Gibbs free energy landscape analysis was also conducted; both the complexes exhibited lower energy minima, indicating a more stable state. Overall, all structural parameters of these complexes maintain their stable conformation [87]. The stability of protein complexes is crucial for biological function and structural integrity. We have proposed these compounds to the global scientific community as they can be further investigated using in vitro and in vivo approaches [88–90]. In conclusion, to effectively prevent and treat Mpox, it is crucial to conduct biochemical and structural studies to validate the efficacy of the repurposed drugs used in this study.

## Conclusion

The analysis of host gene expression from various Mpox infection datasets revealed a notable shared enrichment (MAPK signaling pathway, Transcriptional dysregulation in cancer, and cytokine-cytokine receptors) and a decrease in histones and immune genes. PPI exposed new interactions between transcription factors, histones, and immune gene clusters, suggesting a potential bypass of the host immune response by expressing virotransducers, virokines, and viroreceptors, which could be potential drug targets. MPXV expression of C6R-derived protein K7, which is homologous to VACV K7R, could inhibit the innate immune response and affect histone methylation and epigenetic regulation. Moreover, these findings could help expand drug targets by inhibiting key pathogenic cellular pathways and processes involved in the progression of fatal disease outcomes against MPXV. This research employed a computational drug design approach to identify potent Mpox viral protein C6R-derived protein K7 inhibitors. The lead molecule was screened using several techniques through a virtual screening process. A molecular dynamics analysis was performed to confirm the stability of the binding pose and interactions discovered in the docking investigation. However, the pharmacological and toxicity assessment of the drug molecule and the absence of any toxicity probability confirm an improved absorption and metabolism profile. This study requires further laboratory testing as it solely relied on various computational tools and simulation studies. Nonetheless, it could benefit future researchers working with specific target molecules from a large library to develop effective drugs to treat Mpox.

### Supplementary Information

**Supplementary Material 1.**

## Data Availability

The datasets analyzed during the current study are available in the GEO repository (https://www.ncbi.nlm.nih.gov/geo/). The datasets used and generated in this work are provided in the original article as well as the supplemental materials.

## Abbreviations

*BRCA1*: Breast cancer type 1 susceptibility protein
*CEBPA*-CCAAT: Enhancer binding protein alpha
CPXV: Cowpox Virus
DEG: Differential Expression Genes
EDA: Exploratory Data Analysis
*EGR1*: Early growth response 1
*EGR2*: Early growth response 2
ED: Essential Dynamics
*FOS*: Fos proto-oncogene, AP-1 transcription factor subunit
GEO: Gene Expression Omnibus
GO-BP: Gene Ontology Biological Processes
GO-CC: Gene Ontology Cellular Component
GO-MF: Gene Ontology Molecular Function
KEGG: Kyoto Encylopedia of Genes and Genomes
LogFC: Log fold change
MD: Molecular dynamics
MDS: Molecular Dynamics Simulation
Mpox: Monkeypox
MPXV: Monkeypox virus
*MYC*: Master regulator of cell cycle entry and proliferative metabolism (a proto-oncogene)
PCA: Principal Component Analysis
PPI: Protein–protein interaction
Rg: Radius of gyration
RMSD: Root Mean Square Deviation
*SIRT6*: Sirtuin 6
STAT: Signal transducer and activator of transcription
VACV: Vaccinia vrus
VARV: Variola virus
WHO: World Health Organization
