# Supplementary material for: Expression analysis and mapping of Viral—Host Protein interactions of Poxviridae suggests a lead candidate molecule targeting Mpox
Source: BMC Infect Dis. 2024 May 10;24:483. doi: 10.1186/s12879-024-09332-x (PMC11088078; doi:10.1186/s12879-024-09332-x)

**Supplementary information**

**Expression Analysis and Mapping of Viral - Host Protein interactions of Poxviridae suggests a Lead Candidate Molecule Targeting  Mpox**

**Supplementary information:**

Below the table titles and figures is the electronic supplementary material.

**Supplementary Tables:**

**Supplementary Table S1**: List of GEO datasets on host transcriptome profiles of different Pox infection viruses obtained for the study

**Supplementary Table S2**: Screening of Differential Expression Genes from Host (GSE36854-*Homo sapiens*, GSE21001-*Macaca mulatta*)

**Supplementary Table S3**: Screening of Viral Proteins from the Dataset (GSE11234)

**Supplementary Table S4**: Overlap of Viral proteins from different cell-type (Monkeypox and Vaccinia)

**Supplementary Table S5**: Detailed Description of Viral Protein Function – Monkeypox

**Supplementary Table S1:** List of GEO datasets on host transcriptome profiles of different Pox infection viruses obtained for the study

| **Serial_number** | **GEO_Dataset** | **Experiment** | **Cell_type** | **Virus Type** | **#Samples** | **#Samples to be used for Analysis** | **Sample_Id** | **Name of the samples** |
| --- | --- | --- | --- | --- | --- | --- | --- | --- |
| 1 | GSE36854 | Expression Profiling by Array | Hela_cell_line | Pox infection | 8 | 8 | GSM903055 | Mock |
|  |  |  |  |  |  |  | GSM903056 | Cowpox |
|  |  |  |  |  |  |  | GSM903057 | Vaccinia |
|  |  |  |  |  |  |  | GSM903058 | Monkeypox |
|  |  |  |  |  |  |  | GSM903059 | Mock |
|  |  |  |  |  |  |  | GSM903060 | Cowpox |
|  |  |  |  |  |  |  | GSM903061 | Vaccinia |
|  |  |  |  |  |  |  | GSM903062 | Monkeypox |
| 2 | GSE21001 | Expression Profiling by Array | MK2 Cell line | Monkeypox infection | 9 | 9 | GSM524843 | Mock |
|  |  |  |  |  |  |  | GSM524844 | Monkeypox |
|  |  |  |  |  |  |  | GSM524845 | Monkeypox |
|  |  |  |  |  |  |  | GSM524846 | Mock |
|  |  |  |  |  |  |  | GSM524847 | Monkeypox |
|  |  |  |  |  |  |  | GSM524848 | Monkeypox |
|  |  |  |  |  |  |  | GSM524849 | Mock |
|  |  |  |  |  |  |  | GSM524850 | Monkeypox |
|  |  |  |  |  |  |  | GSM524851 | Monkeypox |
| 3 | GSE219036 | Expression profiling by high throughput sequencing | colon organoids | Monkeypox infection | 24 | 12 | GSM6765206 | colon organoids, mock, no1 |
|  |  |  |  |  |  |  | GSM6765207 | colon organoids, mock, no2 |
|  |  |  |  |  |  |  | GSM6765208 | colon organoids, mock, no3 |
|  |  |  |  |  |  |  | GSM6765209 | colon organoids, MPXV clade IIa, no1 |
|  |  |  |  |  |  |  | GSM6765210 | colon organoids, MPXV clade IIa, no2 |
|  |  |  |  |  |  |  | GSM6765211 | colon organoids, MPXV clade IIa, no3 |
|  |  |  |  |  |  |  | GSM6765212 | colon organoids, MPXV clade IIb, no1 |
|  |  |  |  |  |  |  | GSM6765213 | colon organoids, MPXV clade IIb, no2 |
|  |  |  |  |  |  |  | GSM6765214 | colon organoids, MPXV clade IIb, no3 |
|  |  |  |  |  |  |  | GSM6765215 | colon organoids, MPXV clade I, no1 |
|  |  |  |  |  |  |  | GSM6765225 | colon organoids, MPXV clade I, no2 |
|  |  |  |  |  |  |  | GSM6765240 | colon organoids, MPXV clade I, no3 |
|  |  |  | keratinocytes | Monkeypox infection | 24 | 12 | GSM6765254 | keratinocytes, mock, no1 |
|  |  |  |  |  |  |  | GSM6765268 | keratinocytes, mock, no2 |
|  |  |  |  |  |  |  | GSM6765281 | keratinocytes, mock, no3 |
|  |  |  |  |  |  |  | GSM6765294 | keratinocytes, MPXV clade IIa, no1 |
|  |  |  |  |  |  |  | GSM6765307 | keratinocytes, MPXV clade IIa, no2 |
|  |  |  |  |  |  |  | GSM6765321 | keratinocytes, MPXV clade IIa, no3 |
|  |  |  |  |  |  |  | GSM6765335 | keratinocytes, MPXV clade IIb, no1 |
|  |  |  |  |  |  |  | GSM6765349 | keratinocytes, MPXV clade IIb, no2 |
|  |  |  |  |  |  |  | GSM6765363 | keratinocytes, MPXV clade IIb, no3 |
|  |  |  |  |  |  |  | GSM6765379 | keratinocytes, MPXV clade I, no1 |
|  |  |  |  |  |  |  | GSM6765393 | keratinocytes, MPXV clade I, no2 |
|  |  |  |  |  |  |  | GSM6765408 | keratinocytes, MPXV clade I, no3 |
| 4 | GSE11234 | Expression profiling by array | Primary Human Monocyte(GPL6762) | Monkeypox infection | 124 | 36 | GSM283319 | Mock |
|  |  |  |  |  |  |  | GSM283320 | Mock |
|  |  |  |  |  |  |  | GSM283321 | Mock |
|  |  |  |  |  |  |  | GSM283322 | Mock |
|  |  |  |  |  |  |  | GSM283323 | Mock |
|  |  |  |  |  |  |  | GSM283324 | Mock |
|  |  |  |  |  |  |  | GSM283325 | Mock |
|  |  |  |  |  |  |  | GSM283326 | Mock |
|  |  |  |  |  |  |  | GSM283327 | Mock |
|  |  |  |  |  |  |  | GSM283328 | Mock |
|  |  |  |  |  |  |  | GSM283330 | Mock |
|  |  |  |  |  |  |  | GSM283332 | Mock |
|  |  |  |  |  |  |  | GSM283334 | Mock |
|  |  |  |  |  |  |  | GSM283338 | Mock |
|  |  |  |  |  |  |  | GSM283381 | Mock |
|  |  |  |  |  |  |  | GSM283383 | Mock |
|  |  |  |  |  |  |  | GSM283384 | Mock |
|  |  |  |  |  |  |  | GSM283399 | Mock |
|  |  |  |  |  |  |  | GSM283402 | Mock |
|  |  |  |  |  |  |  | GSM283404 | Mock |
|  |  |  |  |  |  |  | GSM283171 | Monkeypox |
|  |  |  |  |  |  |  | GSM283172 | Monkeypox |
|  |  |  |  |  |  |  | GSM283175 | Monkeypox |
|  |  |  |  |  |  |  | GSM283186 | Monkeypox |
|  |  |  |  |  |  |  | GSM283188 | Monkeypox |
|  |  |  |  |  |  |  | GSM283190 | Monkeypox |
|  |  |  |  |  |  |  | GSM283194 | Monkeypox |
|  |  |  |  |  |  |  | GSM283357 | Monkeypox |
|  |  |  |  |  |  |  | GSM283360 | Monkeypox |
|  |  |  |  |  |  |  | GSM283367 | Monkeypox |
|  |  |  |  |  |  |  | GSM283371 | Monkeypox |
|  |  |  |  |  |  |  | GSM283375 | Monkeypox |
|  |  |  |  |  |  |  | GSM283377 | Monkeypox |
|  |  |  |  |  |  |  | GSM283379 | Monkeypox |
|  |  |  |  |  |  |  | GSM283380 | Monkeypox |
|  |  |  |  |  |  |  | GSM283382 | Monkeypox |
|  |  |  | Primary Human Monocyte | Vaccinia Infection | 124 | 45 | GSM283319 | Mock |
|  |  |  |  |  |  |  | GSM283320 | Mock |
|  |  |  |  |  |  |  | GSM283321 | Mock |
|  |  |  |  |  |  |  | GSM283322 | Mock |
|  |  |  |  |  |  |  | GSM283323 | Mock |
|  |  |  |  |  |  |  | GSM283324 | Mock |
|  |  |  |  |  |  |  | GSM283325 | Mock |
|  |  |  |  |  |  |  | GSM283326 | Mock |
|  |  |  |  |  |  |  | GSM283327 | Mock |
|  |  |  |  |  |  |  | GSM283328 | Mock |
|  |  |  |  |  |  |  | GSM283330 | Mock |
|  |  |  |  |  |  |  | GSM283332 | Mock |
|  |  |  |  |  |  |  | GSM283334 | Mock |
|  |  |  |  |  |  |  | GSM283338 | Mock |
|  |  |  |  |  |  |  | GSM283381 | Mock |
|  |  |  |  |  |  |  | GSM283383 | Mock |
|  |  |  |  |  |  |  | GSM283384 | Mock |
|  |  |  |  |  |  |  | GSM283399 | Mock |
|  |  |  |  |  |  |  | GSM283402 | Mock |
|  |  |  |  |  |  |  | GSM283404 | Mock |
|  |  |  |  |  |  |  | GSM283165 | Vaccinia |
|  |  |  |  |  |  |  | GSM283167 | Vaccinia |
|  |  |  |  |  |  |  | GSM283168 | Vaccinia |
|  |  |  |  |  |  |  | GSM283169 | Vaccinia |
|  |  |  |  |  |  |  | GSM283170 | Vaccinia |
|  |  |  |  |  |  |  | GSM283329 | Vaccinia |
|  |  |  |  |  |  |  | GSM283331 | Vaccinia |
|  |  |  |  |  |  |  | GSM283333 | Vaccinia |
|  |  |  |  |  |  |  | GSM283335 | Vaccinia |
|  |  |  |  |  |  |  | GSM283336 | Vaccinia |
|  |  |  |  |  |  |  | GSM283337 | Vaccinia |
|  |  |  |  |  |  |  | GSM283339 | Vaccinia |
|  |  |  |  |  |  |  | GSM283340 | Vaccinia |
|  |  |  |  |  |  |  | GSM283341 | Vaccinia |
|  |  |  |  |  |  |  | GSM283342 | Vaccinia |
|  |  |  |  |  |  |  | GSM283343 | Vaccinia |
|  |  |  |  |  |  |  | GSM283344 | Vaccinia |
|  |  |  |  |  |  |  | GSM283345 | Vaccinia |
|  |  |  |  |  |  |  | GSM283347 | Vaccinia |
|  |  |  |  |  |  |  | GSM283349 | Vaccinia |
|  |  |  |  |  |  |  | GSM283350 | Vaccinia |
|  |  |  |  |  |  |  | GSM283351 | Vaccinia |
|  |  |  |  |  |  |  | GSM283359 | Vaccinia |
|  |  |  |  |  |  |  | GSM283414 | Vaccinia |
|  |  |  |  |  |  |  | GSM283416 | Vaccinia |
|  |  |  | Primary Human Fibroblast(GPL6762) | Monkeypox infection | 124 | 16 | GSM283173 | Mock |
|  |  |  |  |  |  |  | GSM283177 | Mock |
|  |  |  |  |  |  |  | GSM283187 | Mock |
|  |  |  |  |  |  |  | GSM283189 | Mock |
|  |  |  |  |  |  |  | GSM283192 | Mock |
|  |  |  |  |  |  |  | GSM283196 | Mock |
|  |  |  |  |  |  |  | GSM283203 | Mock |
|  |  |  |  |  |  |  | GSM283222 | Mock |
|  |  |  |  |  |  |  | GSM283396 | Monkeypox |
|  |  |  |  |  |  |  | GSM283397 | Monkeypox |
|  |  |  |  |  |  |  | GSM283398 | Monkeypox |
|  |  |  |  |  |  |  | GSM283400 | Monkeypox |
|  |  |  |  |  |  |  | GSM283401 | Monkeypox |
|  |  |  |  |  |  |  | GSM283403 | Monkeypox |
|  |  |  |  |  |  |  | GSM283405 | Monkeypox |
|  |  |  |  |  |  |  | GSM283378 | Monkeypox |
|  |  |  | Primary Human Fibroblast(GPL6762) | Vaccinia | 124 | 16 | GSM283173 | Mock |
|  |  |  |  |  |  |  | GSM283177 | Mock |
|  |  |  |  |  |  |  | GSM283187 | Mock |
|  |  |  |  |  |  |  | GSM283189 | Mock |
|  |  |  |  |  |  |  | GSM283192 | Mock |
|  |  |  |  |  |  |  | GSM283196 | Mock |
|  |  |  |  |  |  |  | GSM283203 | Mock |
|  |  |  |  |  |  |  | GSM283222 | Mock |
|  |  |  |  |  |  |  | GSM283363 | Vaccinia |
|  |  |  |  |  |  |  | GSM283364 | Vaccinia |
|  |  |  |  |  |  |  | GSM283365 | Vaccinia |
|  |  |  |  |  |  |  | GSM283368 | Vaccinia |
|  |  |  |  |  |  |  | GSM283370 | Vaccinia |
|  |  |  |  |  |  |  | GSM283372 | Vaccinia |
|  |  |  |  |  |  |  | GSM283374 | Vaccinia |
|  |  |  |  |  |  |  | GSM283376 | Vaccinia |
|  |  |  | Hela(GPL6763) | Monkeypox infection | 68 | 10 | GSM283313 | Mock |
|  |  |  |  |  |  |  | GSM283314 | Mock |
|  |  |  |  |  |  |  | GSM283315 | Mock |
|  |  |  |  |  |  |  | GSM283316 | Mock |
|  |  |  |  |  |  |  | GSM283317 | Mock |
|  |  |  |  |  |  |  | GSM283215 | Monkeypox |
|  |  |  |  |  |  |  | GSM283217 | Monkeypox |
|  |  |  |  |  |  |  | GSM283218 | Monkeypox |
|  |  |  |  |  |  |  | GSM283219 | Monkeypox |
|  |  |  |  |  |  |  | GSM283220 | Monkeypox |
|  |  |  | Hela(GPL6763) | Vaccinia | 68 | 10 | GSM283313 | Mock |
|  |  |  |  |  |  |  | GSM283314 | Mock |
|  |  |  |  |  |  |  | GSM283315 | Mock |
|  |  |  |  |  |  |  | GSM283316 | Mock |
|  |  |  |  |  |  |  | GSM283317 | Mock |
|  |  |  |  |  |  |  | GSM283271 | Vaccinia |
|  |  |  |  |  |  |  | GSM283273 | Vaccinia |
|  |  |  |  |  |  |  | GSM283275 | Vaccinia |
|  |  |  |  |  |  |  | GSM283276 | Vaccinia |
|  |  |  |  |  |  |  | GSM283282 | Vaccinia |
|  |  |  | Primary Human Monocyte(GPL6764) | Monkeypox infection | 73 | 12 | GSM283179 | Mock |
|  |  |  |  |  |  |  | GSM283391 | Mock |
|  |  |  |  |  |  |  | GSM283393 | Mock |
|  |  |  |  |  |  |  | GSM283415 | Mock |
|  |  |  |  |  |  |  | GSM283417 | Mock |
|  |  |  |  |  |  |  | GSM283418 | Mock |
|  |  |  |  |  |  |  | GSM283419 | Mock |
|  |  |  |  |  |  |  | GSM283180 | Monkeypox |
|  |  |  |  |  |  |  | GSM283181 | Monkeypox |
|  |  |  |  |  |  |  | GSM283182 | Monkeypox |
|  |  |  |  |  |  |  | GSM283183 | Monkeypox |
|  |  |  |  |  |  |  | GSM283184 | Monkeypox |
|  |  |  | Primary Human Monocyte(GPL6764) | Vaccinia | 73 | 11 | GSM283179 | Mock |
|  |  |  |  |  |  |  | GSM283391 | Mock |
|  |  |  |  |  |  |  | GSM283393 | Mock |
|  |  |  |  |  |  |  | GSM283415 | Mock |
|  |  |  |  |  |  |  | GSM283417 | Mock |
|  |  |  |  |  |  |  | GSM283418 | Mock |
|  |  |  |  |  |  |  | GSM283419 | Mock |
|  |  |  |  |  |  |  | GSM283160 | Vaccinia |
|  |  |  |  |  |  |  | GSM283174 | Vaccinia |
|  |  |  |  |  |  |  | GSM283176 | Vaccinia |
|  |  |  |  |  |  |  | GSM283178 | Vaccinia |
| 4 | GSE219036 | Expression Profiling by High throughput sequencing | colon organoids | Monkeypox | 24 | 12 | GSM6765206 | Mock |
|  |  |  |  |  |  |  | GSM6765207 | Mock |
|  |  |  |  |  |  |  | GSM6765208 | Mock |
|  |  |  |  |  |  |  | GSM6765209 | MPXV clade IIa(Liberia) |
|  |  |  |  |  |  |  | GSM6765210 | MPXV clade IIa(Liberia) |
|  |  |  |  |  |  |  | GSM6765211 | MPXV clade IIa(Liberia) |
|  |  |  |  |  |  |  | GSM6765212 | MPXV clade IIb(MPXV-2022) |
|  |  |  |  |  |  |  | GSM6765213 | MPXV clade IIb(MPXV-2022) |
|  |  |  |  |  |  |  | GSM6765214 | MPXV clade IIb(MPXV-2022) |
|  |  |  |  |  |  |  | GSM6765215 | MPXV clade I(Zr-599) |
|  |  |  |  |  |  |  | GSM6765225 | MPXV clade I(Zr-599) |
|  |  |  |  |  |  |  | GSM6765240 | MPXV clade I(Zr-599) |
|  |  | Expression Profiling by High throughput sequencing | Keratinocytes | Monkeypox | 24 | 12 | GSM6765254 | Mock |
|  |  |  |  |  |  |  | GSM6765268 | Mock |
|  |  |  |  |  |  |  | GSM6765281 | Mock |
|  |  |  |  |  |  |  | GSM6765294 | MPXV clade IIa(Liberia) |
|  |  |  |  |  |  |  | GSM6765307 | MPXV clade IIa(Liberia) |
|  |  |  |  |  |  |  | GSM6765321 | MPXV clade IIa(Liberia) |
|  |  |  |  |  |  |  | GSM6765335 | MPXV clade IIb(MPXV-2022) |
|  |  |  |  |  |  |  | GSM6765349 | MPXV clade IIb(MPXV-2022) |
|  |  |  |  |  |  |  | GSM6765363 | MPXV clade IIb(MPXV-2022) |
|  |  |  |  |  |  |  | GSM6765379 | MPXV clade I(Zr-599) |
|  |  |  |  |  |  |  | GSM6765393 | MPXV clade I(Zr-599) |
|  |  |  |  |  |  |  | GSM6765408 | MPXV clade I(Zr-599) |
|  |  |  |  |  |  |  |  |  |

**Supplementary Table S2:** Screening of Differential Expression Genes from Host (*Homo sapiens* and *Macaca mulatta*)

| #Screening of Differential Expression Genes from Host-***Homo sapiens*** | | | |
| --- | --- | --- | --- |
| **#Mock VS Monkeypox-infected -Total of 111** | | | |
| **Gene.symbol** | **Gene.title** | **P.Value** | **logFC** |
| *HECW2* | HECT, C2 and WW domain containing E3 ubiquitin protein ligase 2 | 0.0000984 | 3.9978114 |
| *MARCH4* | membrane associated ring-CH-type finger 4 | 0.0004364 | 2.8027037 |
| *FAM46B* | family with sequence similarity 46 member B | 0.0015067 | 2.2271687 |
| *DLC1* | DLC1 Rho GTPase activating protein | 0.0018542 | 2.3482677 |
| *BRCA1* | BRCA1, DNA repair associated | 0.0021378 | 2.7196241 |
| *CDH6* | cadherin 6 | 0.002468 | 2.4426233 |
| *F2* | coagulation factor II, thrombin | 0.0025019 | 3.104843 |
| *PRR16* | proline rich 16 | 0.0029344 | 2.0798768 |
| *LOC439938* | uncharacterized LOC439938 | 0.0032188 | 2.0800355 |
| *ZAP70* | zeta chain of T cell receptor associated protein kinase 70 | 0.0039068 | 3.3333274 |
| *NIM1K* | NIM1 serine/threonine protein kinase | 0.0041856 | 2.1150222 |
| *DLGAP1* | DLG associated protein 1 | 0.0045253 | 2.5512779 |
| *LOC400707* | uncharacterized LOC400707 | 0.0045557 | 2.2330032 |
| *DOCK3* | dedicator of cytokinesis 3 | 0.0049992 | 2.8624326 |
| *RS1* | retinoschisin 1 | 0.0064475 | 2.0764553 |
| *SCN7A* | sodium voltage-gated channel alpha subunit 7 | 0.0070867 | 2.0799897 |
| *TPH2* | tryptophan hydroxylase 2 | 0.0076185 | 2.8956923 |
| *BMF* | Bcl2 modifying factor | 0.0087379 | 2.9729003 |
| *IFIT2* | interferon induced protein with tetratricopeptide repeats 2 | 0.0091366 | 2.2105943 |
| *HIST1H4A* | histone cluster 1, H4a | 0.0000242 | -8.073908 |
| *HIST4H4* | histone cluster 4, H4 | 0.0000346 | -6.345056 |
| *HIST2H4B* | histone cluster 2, H4b | 0.0000909 | -5.670249 |
| *HIST1H4F* | histone cluster 1, H4f | 0.0001188 | -5.885721 |
| *HIST1H4E* | histone cluster 1, H4e | 0.0001194 | -6.89889 |
| *HIST1H4H* | histone cluster 1, H4h | 0.0001636 | -6.387779 |
| *HIST1H4I* | histone cluster 1, H4i | 0.0001665 | -5.869447 |
| *CXCL8* | C-X-C motif chemokine ligand 8 | 0.0002234 | -8.327572 |
| *HIST1H3B* | histone cluster 1, H3b | 0.0002304 | -5.38777 |
| *HIST1H2AM* | histone cluster 1, H2am | 0.0002564 | -5.955769 |
| *CXCL2* | C-X-C motif chemokine ligand 2 | 0.0002875 | -4.666182 |
| *LIF* | leukemia inhibitory factor | 0.0002906 | -3.596812 |
| *MAFF* | MAF bZIP transcription factor F | 0.0003062 | -3.216409 |
| *CXCL3* | C-X-C motif chemokine ligand 3 | 0.000361 | -5.221978 |
| *CXCL1* | C-X-C motif chemokine ligand 1 | 0.0003624 | -8.1518 |
| *EREG* | epiregulin | 0.0003632 | -3.385137 |
| *HIST1H4J* | histone cluster 1, H4j | 0.0004033 | -5.914706 |
| *DUSP6* | dual specificity phosphatase 6 | 0.0004657 | -3.311468 |
| *IL6* | interleukin 6 | 0.0005027 | -4.498094 |
| *AREG* | amphiregulin | 0.0005567 | -3.064727 |
| *HIST1H4K* | histone cluster 1, H4k | 0.000576 | -5.783163 |
| *LMO2* | LIM domain only 2 | 0.0006114 | -2.537544 |
| *MFSD2A* | major facilitator superfamily domain containing 2A | 0.0006728 | -3.083554 |
| *TAAR1* | trace amine associated receptor 1 | 0.0007345 | -3.409317 |
| *HIST1H3H* | histone cluster 1, H3h | 0.0007455 | -4.744626 |
| *IL4R* | interleukin 4 receptor | 0.0007868 | -2.492118 |
| *HIST2H3D* | histone cluster 2, H3d | 0.0008159 | -3.467614 |
| *PHLDA1* | pleckstrin homology like domain family A member 1 | 0.0009178 | -2.344782 |
| *SPRY4* | sprouty RTK signaling antagonist 4 | 0.0009594 | -5.007363 |
| *RAPGEF3* | Rap guanine nucleotide exchange factor 3 | 0.000996 | -3.506336 |
| *RGN* | regucalcin | 0.0010214 | -2.434924 |
| *SPRY2* | sprouty RTK signaling antagonist 2 | 0.001056 | -2.903058 |
| *SMOX* | spermine oxidase | 0.0010565 | -2.234561 |
| *DUOX1* | dual oxidase 1 | 0.0010743 | -2.516635 |
| *IER3* | immediate early response 3 | 0.0010845 | -2.86336 |
| *HIST1H4G* | histone cluster 1, H4g | 0.0011274 | -4.971127 |
| *DUSP5* | dual specificity phosphatase 5 | 0.0012149 | -2.326492 |
| *EGR1* | early growth response 1 | 0.0012271 | -5.545363 |
| *TNFRSF1B* | TNF receptor superfamily member 1B | 0.0012357 | -2.331382 |
| *TLR2* | toll like receptor 2 | 0.0012898 | -2.285576 |
| *HMGA2* | high mobility group AT-hook 2 | 0.0013562 | -2.259355 |
| *PTGS2* | prostaglandin-endoperoxide synthase 2 | 0.0013607 | -4.183853 |
| *HIST1H2AL* | histone cluster 1, H2al | 0.0014106 | -4.136425 |
| *STX11* | syntaxin 11 | 0.001427 | -2.390954 |
| *TNFSF14* | tumor necrosis factor superfamily member 14 | 0.001508 | -2.968895 |
| *HIST1H2AB* | histone cluster 1, H2ab | 0.0015358 | -4.179587 |
| *IL1RAP* | interleukin 1 receptor accessory protein | 0.0015841 | -2.10076 |
| *HIST1H3F* | histone cluster 1, H3f | 0.0017296 | -4.016235 |
| *NEU4* | neuraminidase 4 | 0.0018152 | -3.055168 |
| *IL11* | interleukin 11 | 0.0018458 | -2.901475 |
| *F5* | coagulation factor V | 0.0018813 | -2.401618 |
| *ERRFI1* | ERBB receptor feedback inhibitor 1 | 0.0019534 | -2.133797 |
| *EGR2* | early growth response 2 | 0.0023759 | -2.464999 |
| *TRIM15* | tripartite motif containing 15 | 0.0025672 | -2.318044 |
| *ADGRE4P* | adhesion G protein-coupled receptor E4, pseudogene | 0.0026113 | -4.655124 |
| *HIST2H3A* | histone cluster 2, H3a | 0.0026371 | -3.146707 |
| *HIST1H2AJ* | histone cluster 1, H2aj | 0.0026741 | -3.283677 |
| *HIST3H2A* | histone cluster 3, H2a | 0.0032811 | -3.163485 |
| *HIST1H3D* | histone cluster 1, H3d | 0.003438 | -2.820574 |
| *HIST2H2AC* | histone cluster 2, H2ac | 0.003533 | -4.411015 |
| *LIMD2* | LIM domain containing 2 | 0.0036316 | -2.420246 |
| *C2orf48* | chromosome 2 open reading frame 48 | 0.00372 | -2.029292 |
| *ADORA2A* | adenosine A2a receptor | 0.0037326 | -2.269464 |
| *GCM1* | glial cells missing homolog 1 | 0.0037819 | -4.465575 |
| *HIST1H2AD* | histone cluster 1, H2ad | 0.0038937 | -3.706317 |
| *RELB* | RELB proto-oncogene, NF-kB subunit | 0.0039971 | -2.94691 |
| *PITPNC1* | phosphatidylinositol transfer protein, cytoplasmic 1 | 0.0040612 | -2.047146 |
| *GABRD* | gamma-aminobutyric acid type A receptor delta subunit | 0.0041467 | -2.445264 |
| *HIST1H2BJ* | histone cluster 1, H2bj | 0.0047016 | -2.939948 |
| *PPP1R14C* | protein phosphatase 1 regulatory inhibitor subunit 14C | 0.0047324 | -2.195505 |
| *SNRPN///IPW* | small nuclear ribonucleoprotein polypeptide N///imprinted in Prader-Willi syndrome (non-protein coding) | 0.0048784 | -2.240321 |
| *CXorf36* | chromosome X open reading frame 36 | 0.0053206 | -2.095718 |
| *HIST1H2AE* | histone cluster 1, H2ae | 0.0053337 | -3.400872 |
| *ST3GAL5* | ST3 beta-galactoside alpha-2,3-sialyltransferase 5 | 0.0055171 | -2.819753 |
| *DNAJB3* | DnaJ heat shock protein family (Hsp40) member B3 | 0.0055896 | -2.543605 |
| *ZC3H12A* | zinc finger CCCH-type containing 12A | 0.0059172 | -2.408162 |
| *HIST2H2AB* | histone cluster 2, H2ab | 0.0059244 | -3.099766 |
| *SH2B3* | SH2B adaptor protein 3 | 0.0060912 | -2.104562 |
| *LINC00937* | long intergenic non-protein coding RNA 937 | 0.0061521 | -2.094512 |
| *ARC* | activity regulated cytoskeleton associated protein | 0.0068042 | -3.082653 |
| *HIST1H2AG* | histone cluster 1, H2ag | 0.0072461 | -2.244624 |
| *CSF2* | colony stimulating factor 2 | 0.0072713 | -3.799008 |
| *PTX3* | pentraxin 3 | 0.0076606 | -3.104503 |
| *ZBTB46* | zinc finger and BTB domain containing 46 | 0.0078274 | -2.610334 |
| *ASGR1* | asialoglycoprotein receptor 1 | 0.0079105 | -2.300311 |
| *HIST1H2BE* | histone cluster 1, H2be | 0.008075 | -2.710725 |
| *HIST1H2BF* | histone cluster 1, H2bf | 0.0080773 | -2.693191 |
| *C5orf47* | chromosome 5 open reading frame 47 | 0.0081525 | -2.065606 |
| *HIST1H2BH* | histone cluster 1, H2bh | 0.0083507 | -2.503048 |
| *HIST1H2BO* | histone cluster 1, H2bo | 0.0091695 | -2.727721 |
| *HIST3H2BB* | histone cluster 3, H2bb | 0.0097931 | -2.592531 |
| *HIST1H2AH* | histone cluster 1, H2ah | 0.0099421 | -2.29884 |
| ***Mock vs Cowpox-infected - Total of 217*** | | | |
| ***Gene.symbol*** | **Gene.title** | **P.Value** | **logFC** |
| *HECW2* | HECT, C2 and WW domain containing E3 ubiquitin protein ligase 2 | 5.476E-05 | 3.9830175 |
| *BMF* | Bcl2 modifying factor | 0.0001782 | 3.1645275 |
| *IFIT2* | interferon induced protein with tetratricopeptide repeats 2 | 0.0004177 | 2.6033718 |
| *KCNMB1* | potassium calcium-activated channel subfamily M regulatory beta subunit 1 | 0.000596 | 2.2477755 |
| *MAP1A* | microtubule associated protein 1A | 0.0007269 | 2.5541693 |
| *UNC5D* | unc-5 netrin receptor D | 0.0007729 | 2.1771319 |
| *March4* | membrane associated ring-CH-type finger 4 | 0.0008005 | 2.8237912 |
| *CDH6* | cadherin 6 | 0.0013048 | 2.188722 |
| *CPA4* | carboxypeptidase A4 | 0.0014432 | 2.4444223 |
| *ADRA2A* | adrenoceptor alpha 2A | 0.0014981 | 2.2968286 |
| *KRT33A* | keratin 33A | 0.0016739 | 2.0153404 |
| *SFRP1* | secreted frizzled related protein 1 | 0.0016999 | 2.4858113 |
| *PROC* | protein C, inactivator of coagulation factors Va and VIIIa | 0.0019185 | 2.0195342 |
| *PRKCB* | protein kinase C beta | 0.0023744 | 2.1254365 |
| *ZBED2* | zinc finger BED-type containing 2 | 0.0026672 | 2.0844235 |
| *KRT6C* | keratin 6C | 0.0027356 | 2.5352734 |
| *SERTM1* | serine rich and transmembrane domain containing 1 | 0.0027661 | 2.0720248 |
| *CCDC121* | coiled-coil domain containing 121 | 0.0030305 | 2.2898048 |
| *ZNF518B* | zinc finger protein 518B | 0.0033321 | 2.1993249 |
| *RYR3* | ryanodine receptor 3 | 0.0063426 | 2.3406127 |
| *CARMN* | cardiac mesoderm enhancer-associated non-coding RNA | 0.0069031 | 2.4704362 |
| *EDN2* | endothelin 2 | 0.0084171 | 3.6679484 |
| *FXYD2* | FXYD domain containing ion transport regulator 2 | 5.87E-06 | -8.929282 |
| *HIST1H4E* | histone cluster 1, H4e | 1.109E-05 | -7.254054 |
| *HIST1H4A* | histone cluster 1, H4a | 1.266E-05 | -8.49546 |
| *CXCL1* | C-X-C motif chemokine ligand 1 | 1.338E-05 | -8.605451 |
| *HIST2H4B* | histone cluster 2, H4b | 1.593E-05 | -6.012015 |
| *CXCL2* | C-X-C motif chemokine ligand 2 | 2.195E-05 | -5.326409 |
| *HIST1H4H* | histone cluster 1, H4h | 2.594E-05 | -6.723807 |
| *HIST1H3B* | histone cluster 1, H3b | 2.908E-05 | -5.400952 |
| *HIST1H4I* | histone cluster 1, H4i | 2.937E-05 | -6.266132 |
| *HIST1H2AM* | histone cluster 1, H2am | 3.326E-05 | -6.331939 |
| *HIST4H4* | histone cluster 4, H4 | 4.335E-05 | -6.934892 |
| *KLF15* | Kruppel like factor 15 | 0.0000442 | -4.475575 |
| *HIST1H4F* | histone cluster 1, H4f | 5.327E-05 | -6.119202 |
| *HIST1H4J* | histone cluster 1, H4j | 5.446E-05 | -5.736358 |
| *HIST1H4K* | histone cluster 1, H4k | 7.258E-05 | -5.414958 |
| *PTGS2* | prostaglandin-endoperoxide synthase 2 | 0.0000728 | -3.878784 |
| *HIST1H3H* | histone cluster 1, H3h | 8.092E-05 | -4.988006 |
| *CXCL8* | C-X-C motif chemokine ligand 8 | 8.769E-05 | -7.943828 |
| *IL6* | interleukin 6 | 0.0001258 | -4.265196 |
| *AXIN2* | axin 2 | 0.0001329 | -3.446273 |
| *HIST1H2AL* | histone cluster 1, H2al | 0.0001351 | -4.703864 |
| *TBX2* | T-box 2 | 0.0001395 | -3.48143 |
| *LIF* | leukemia inhibitory factor | 0.000158 | -3.406426 |
| *HIST2H3D* | histone cluster 2, H3d | 0.0001602 | -3.665069 |
| *HIST1H4G* | histone cluster 1, H4g | 0.0001777 | -4.746389 |
| *RCOR2* | REST corepressor 2 | 0.0001781 | -4.238473 |
| *HIST1H2AD* | histone cluster 1, H2ad | 0.0001878 | -3.957126 |
| *CXCL3* | C-X-C motif chemokine ligand 3 | 0.00021 | -5.442902 |
| *MAFF* | MAF bZIP transcription factor F | 0.0002139 | -3.640264 |
| *AREG* | amphiregulin | 0.0002225 | -3.182413 |
| *TNIP3* | TNFAIP3 interacting protein 3 | 0.0002259 | -3.724246 |
| *HIST1H2AB* | histone cluster 1, H2ab | 0.0002272 | -4.365203 |
| *HIST2H3A* | histone cluster 2, H3a | 0.0002305 | -3.355649 |
| *ANKRD30BP2* | ankyrin repeat domain 30B pseudogene 2 | 0.0002403 | -3.082721 |
| *ASPHD2* | aspartate beta-hydroxylase domain containing 2 | 0.0002653 | -2.759073 |
| *NFKBIZ* | NFKB inhibitor zeta | 0.000266 | -3.130129 |
| *C2CD4C* | C2 calcium dependent domain containing 4C | 0.0002728 | -3.200503 |
| *IL1B* | interleukin 1 beta | 0.0002783 | -3.276755 |
| *DACT3* | dishevelled binding antagonist of beta catenin 3 | 0.0002824 | -2.719548 |
| *RELB* | RELB proto-oncogene, NF-kB subunit | 0.0002933 | -3.195838 |
| *HIST1H2AE* | histone cluster 1, H2ae | 0.0003109 | -3.332308 |
| *EXOC2* | exocyst complex component 2 | 0.0003259 | -3.548673 |
| *EREG* | epiregulin | 0.0003276 | -2.648531 |
| *HIST3H2A* | histone cluster 3, H2a | 0.0003493 | -3.178122 |
| *HIST1H2AJ* | histone cluster 1, H2aj | 0.0003518 | -3.285388 |
| *VWCE* | von Willebrand factor C and EGF domains | 0.0003602 | -2.707294 |
| *DUSP5* | dual specificity phosphatase 5 | 0.0003856 | -2.764249 |
| *SPRY2* | sprouty RTK signaling antagonist 2 | 0.0004057 | -2.939623 |
| *CEBPA* | CCAAT/enhancer binding protein alpha | 0.000424 | -2.720171 |
| *BIK* | BCL2 interacting killer | 0.0004272 | -2.601629 |
| *DUSP6* | dual specificity phosphatase 6 | 0.0004539 | -3.05315 |
| *FNDC5* | fibronectin type III domain containing 5 | 0.0004724 | -2.484052 |
| *MYOZ3* | myozenin 3 | 0.0004772 | -2.486449 |
| *HIST1H3D* | histone cluster 1, H3d | 0.0004835 | -2.917252 |
| *HIST1H3F* | histone cluster 1, H3f | 0.0004981 | -4.267649 |
| *MYC* | v-myc avian myelocytomatosis viral oncogene homolog | 0.0005148 | -2.750833 |
| *FUT1* | fucosyltransferase 1 (H blood group) | 0.0005205 | -3.149016 |
| *ASGR1* | asialoglycoprotein receptor 1 | 0.000524 | -2.527228 |
| *PHLDA1* | pleckstrin homology like domain family A member 1 | 0.0005336 | -2.584821 |
| *IGHV3-30* | immunoglobulin heavy variable 3-30 | 0.0005364 | -4.164686 |
| *TNFRSF1B* | TNF receptor superfamily member 1B | 0.0005435 | -2.582027 |
| *N4BP2L1* | NEDD4 binding protein 2 like 1 | 0.000545 | -2.623125 |
| *EAF1* | ELL associated factor 1 | 0.0005764 | -2.632725 |
| *HIST1H2BF* | histone cluster 1, H2bf | 0.0005851 | -2.956828 |
| *HIST2H2AC* | histone cluster 2, H2ac | 0.0005882 | -4.113081 |
| *HIST1H2BE* | histone cluster 1, H2be | 0.0006125 | -2.969088 |
| *HIST1H2BJ* | histone cluster 1, H2bj | 0.0006189 | -3.034721 |
| *CMTM7* | CKLF like MARVEL transmembrane domain containing 7 | 0.0006227 | -2.509426 |
| *GJB2* | gap junction protein beta 2 | 0.0006287 | -2.552089 |
| *IL4R* | interleukin 4 receptor | 0.0006415 | -2.443398 |
| *MFSD2A* | major facilitator superfamily domain containing 2A | 0.000645 | -3.39303 |
| *IL1A* | interleukin 1 alpha | 0.0006558 | -3.38067 |
| *ARC* | activity regulated cytoskeleton associated protein | 0.0006593 | -3.022639 |
| *IL1RAP* | interleukin 1 receptor accessory protein | 0.0006725 | -2.336373 |
| *PTX3* | pentraxin 3 | 0.0006828 | -3.766664 |
| *RASD1* | ras related dexamethasone induced 1 | 0.0007079 | -2.275516 |
| *HBEGF* | heparin binding EGF like growth factor | 0.0007298 | -2.336155 |
| *HIST2H2AB* | histone cluster 2, H2ab | 0.0007336 | -2.816909 |
| *DRD5* | dopamine receptor D5 | 0.0007456 | -2.340945 |
| *TLR2* | toll like receptor 2 | 0.000789 | -2.935251 |
| *ERRFI1* | ERBB receptor feedback inhibitor 1 | 0.0008007 | -2.158451 |
| *ICAM1* | intercellular adhesion molecule 1 | 0.0008175 | -2.119842 |
| *KIF27* | kinesin family member 27 | 0.0008243 | -2.085877 |
| *NEU4* | neuraminidase 4 | 0.0008301 | -3.456934 |
| *LOC100286922* | DnaJ heat shock protein family (Hsp40) member B3 pseudogene | 0.0008459 | -3.564614 |
| *FST* | follistatin | 0.0008598 | -2.474327 |
| *HIST1H2BD* | histone cluster 1, H2bd | 0.0008806 | -2.718283 |
| *HIST1H2BO* | histone cluster 1, H2bo | 0.0009008 | -2.993393 |
| *SPRY4* | sprouty RTK signaling antagonist 4 | 0.0009044 | -4.514354 |
| *FAM131B* | family with sequence similarity 131 member B | 0.0009192 | -2.283901 |
| *HIST3H2BB* | histone cluster 3, H2bb | 0.0009362 | -2.855814 |
| *SP5* | Sp5 transcription factor | 0.0009711 | -3.164359 |
| *HIST1H2BH* | histone cluster 1, H2bh | 0.0009926 | -2.710992 |
| *LOC730961* | uncharacterized LOC730961 | 0.0009992 | -2.140891 |
| *MAP3K6* | mitogen-activated protein kinase kinase kinase 6 | 0.0010128 | -2.173727 |
| *RRAGD* | Ras related GTP binding D | 0.0010138 | -3.676163 |
| *DNAJA4* | DnaJ heat shock protein family (Hsp40) member A4 | 0.0010298 | -2.220533 |
| *SLC2A14* | solute carrier family 2 member 14 | 0.0010322 | -2.463681 |
| *HIST1H2BL* | histone cluster 1, H2bl | 0.0010375 | -2.690441 |
| *LOC101060524///DRD5P2* | D(1B) dopamine receptor-like///dopamine receptor D5 pseudogene 2 | 0.001133 | -2.247379 |
| *HS3ST1* | heparan sulfate-glucosamine 3-sulfotransferase 1 | 0.0011574 | -2.225837 |
| *EPHB3* | EPH receptor B3 | 0.0011732 | -2.10467 |
| *DNAJB3* | DnaJ heat shock protein family (Hsp40) member B3 | 0.0012015 | -4.122247 |
| *OLR1* | oxidized low density lipoprotein receptor 1 | 0.0012046 | -2.809855 |
| *STX11* | syntaxin 11 | 0.0012133 | -2.42647 |
| *TUBB2B* | tubulin beta 2B class IIb | 0.0012512 | -3.213359 |
| *SRRM3* | serine/arginine repetitive matrix 3 | 0.0012821 | -2.914191 |
| *ABCG4* | ATP binding cassette subfamily G member 4 | 0.0013039 | -3.186571 |
| *SOCS1* | suppressor of cytokine signaling 1 | 0.0013085 | -2.349555 |
| *RREB1* | ras responsive element binding protein 1 | 0.0013146 | -2.015597 |
| *RASSF2* | Ras association domain family member 2 | 0.001326 | -3.236112 |
| *PRKAG2-AS1* | PRKAG2 antisense RNA 1 | 0.0013593 | -2.105938 |
| *PALM3* | paralemmin 3 | 0.0013891 | -2.142547 |
| *FAM69B* | family with sequence similarity 69 member B | 0.0013999 | -2.928063 |
| *HEYL* | hes related family bHLH transcription factor with YRPW motif-like | 0.0014031 | -2.136605 |
| *CCDC181* | coiled-coil domain containing 181 | 0.0014255 | -3.52692 |
| *ST3GAL5* | ST3 beta-galactoside alpha-2,3-sialyltransferase 5 | 0.0014311 | -2.730645 |
| *CRYBA2* | crystallin beta A2 | 0.0014635 | -2.153864 |
| *FGF7* | fibroblast growth factor 7 | 0.0015099 | -3.254526 |
| *HIST1H2BI* | histone cluster 1, H2bi | 0.0015213 | -2.508248 |
| *HIST1H2AG* | histone cluster 1, H2ag | 0.0015497 | -2.195646 |
| *SLC26A10* | solute carrier family 26 member 10 | 0.0015897 | -2.146299 |
| *DKK1* | dickkopf WNT signaling pathway inhibitor 1 | 0.0016751 | -2.359641 |
| *HIST2H2AA4* | histone cluster 2, H2aa4 | 0.0016907 | -2.227578 |
| *PTPN4* | protein tyrosine phosphatase, non-receptor type 4 | 0.0016957 | -2.085152 |
| *EGR1* | early growth response 1 | 0.0016975 | -3.968707 |
| *HIST1H2BB* | histone cluster 1, H2bb | 0.0017059 | -2.333766 |
| *HIST1H2AH* | histone cluster 1, H2ah | 0.0017398 | -2.267823 |
| *NTF4* | neurotrophin 4 | 0.0017614 | -2.549414 |
| *PANK3* | pantothenate kinase 3 | 0.0017644 | -2.102116 |
| *LOC105370424* | uncharacterized LOC105370424 | 0.0018182 | -2.331329 |
| *TBX21* | T-box 21 | 0.0018499 | -3.097354 |
| *IGHG1* | immunoglobulin heavy constant gamma 1 (G1m marker) | 0.0018734 | -3.313785 |
| *HIST1H2AK* | histone cluster 1, H2ak | 0.0019723 | -2.410059 |
| *DUOX1* | dual oxidase 1 | 0.0020367 | -2.686441 |
| *ABL2* | ABL proto-oncogene 2, non-receptor tyrosine kinase | 0.0020482 | -2.106585 |
| *IGHV3-48///IGHV3-69-1* | immunoglobulin heavy variable 3-48///immunoglobulin heavy variable 3-69-1 (pseudogene) | 0.0020743 | -3.16258 |
| *HIST2H2BE* | histone cluster 2, H2be | 0.0020888 | -2.211381 |
| *KATNAL2* | katanin catalytic subunit A1 like 2 | 0.0021189 | -2.023526 |
| *LOC283454* | uncharacterized LOC283454 | 0.002132 | -2.411666 |
| *CCDC146* | coiled-coil domain containing 146 | 0.00217 | -2.293629 |
| *ZBTB46* | zinc finger and BTB domain containing 46 | 0.002219 | -3.008363 |
| *GARNL3* | GTPase activating Rap/RanGAP domain like 3 | 0.0024157 | -2.180298 |
| *IL12A* | interleukin 12A | 0.0024516 | -3.391426 |
| *NR4A1* | nuclear receptor subfamily 4 group A member 1 | 0.0024633 | -3.021269 |
| *GTF2IRD2* | GTF2I repeat domain containing 2 | 0.0024748 | -2.100956 |
| *RAPGEF3* | Rap guanine nucleotide exchange factor 3 | 0.0025261 | -2.895572 |
| *GDNF* | glial cell derived neurotrophic factor | 0.0026853 | -3.004852 |
| *PPP1R14C* | protein phosphatase 1 regulatory inhibitor subunit 14C | 0.0027275 | -2.532465 |
| *DYX1C1-CCPG1///CCPG1* | DYX1C1-CCPG1 readthrough (NMD candidate)///cell cycle progression 1 | 0.0027547 | -2.21903 |
| *PTGER4* | prostaglandin E receptor 4 | 0.002773 | -2.196064 |
| *ZNF608* | zinc finger protein 608 | 0.0028241 | -3.172254 |
| *DYRK1A* | dual specificity tyrosine phosphorylation regulated kinase 1A | 0.0029114 | -2.470799 |
| *HIST2H2BD* | histone cluster 2, H2bd (pseudogene) | 0.0029388 | -2.250518 |
| *TNFSF14* | tumor necrosis factor superfamily member 14 | 0.0029613 | -2.863988 |
| *ALDH8A1* | aldehyde dehydrogenase 8 family member A1 | 0.0029623 | -2.181566 |
| *SPOCK2* | sparc/osteonectin, cwcv and kazal-like domains proteoglycan (testican) 2 | 0.0031272 | -2.013714 |
| *PPP1R37* | protein phosphatase 1 regulatory subunit 37 | 0.0032429 | -2.756739 |
| *IRS1* | insulin receptor substrate 1 | 0.0032885 | -2.082056 |
| *MTTP* | microsomal triglyceride transfer protein | 0.0033758 | -3.131518 |
| *CXCL14* | C-X-C motif chemokine ligand 14 | 0.0033991 | -2.164828 |
| *NRG2* | neuregulin 2 | 0.0034271 | -2.583691 |
| *SPRED2* | sprouty related EVH1 domain containing 2 | 0.0034366 | -2.877027 |
| *EPB41L5* | erythrocyte membrane protein band 4.1 like 5 | 0.0035385 | -2.423001 |
| *HIST1H2BG* | histone cluster 1, H2bg | 0.004146 | -2.13176 |
| *TPTE* | transmembrane phosphatase with tensin homology | 0.0042246 | -2.311991 |
| *SPRY1* | sprouty RTK signaling antagonist 1 | 0.0044355 | -3.460231 |
| *HOXA3* | homeobox A3 | 0.0045313 | -3.750133 |
| *SP8* | Sp8 transcription factor | 0.0046853 | -2.88912 |
| *RGN* | regucalcin | 0.0047348 | -2.494001 |
| *RAI2* | retinoic acid induced 2 | 0.0047543 | -2.560291 |
| *F5* | coagulation factor V | 0.004846 | -2.071181 |
| *GSAP* | gamma-secretase activating protein | 0.0048975 | -2.361102 |
| *CSF2* | colony stimulating factor 2 | 0.0051279 | -2.996683 |
| *LOC105376714///SLC30A4* | uncharacterized LOC105376714///solute carrier family 30 member 4 | 0.0051773 | -2.588542 |
| *GGT7* | gamma-glutamyltransferase 7 | 0.0060143 | -2.01369 |
| *APOE* | apolipoprotein E | 0.0060702 | -2.212716 |
| *ESR2* | estrogen receptor 2 | 0.0061244 | -2.804363 |
| *GBX1* | gastrulation brain homeobox 1 | 0.006179 | -2.25775 |
| *ZC3H12C* | zinc finger CCCH-type containing 12C | 0.0062695 | -2.015989 |
| *MAP3K15* | mitogen-activated protein kinase kinase kinase 15 | 0.0070033 | -2.063341 |
| *LINC01341* | long intergenic non-protein coding RNA 1341 | 0.0070668 | -2.108298 |
| *COL8A2* | collagen type VIII alpha 2 chain | 0.0071361 | -3.087237 |
| *NOS1AP* | nitric oxide synthase 1 adaptor protein | 0.0072228 | -2.764941 |
| *CCL7* | C-C motif chemokine ligand 7 | 0.007417 | -2.058489 |
| *HIST1H2AA* | histone cluster 1, H2aa | 0.0075616 | -2.362939 |
| *CCL20* | C-C motif chemokine ligand 20 | 0.0076193 | -4.882308 |
| *C2orf66* | chromosome 2 open reading frame 66 | 0.007811 | -2.583956 |
| *ZNF853* | zinc finger protein 853 | 0.0078665 | -2.638853 |
| *CRYBB3* | crystallin beta B3 | 0.0078793 | -2.646986 |
| *AEBP1* | AE binding protein 1 | 0.0079139 | -2.452182 |
| *NRTN* | neurturin | 0.0084201 | -2.189626 |
| *HIST1H2AC* | histone cluster 1, H2ac | 0.0086495 | -2.425324 |
| *TNF* | tumor necrosis factor | 0.0087634 | -2.631771 |
| *TSNARE1* | t-SNARE domain containing 1 | 0.0099533 | -2.513453 |
| ***Mock VS Vaccinia-infected -Total of 162*** | | | |
| ***Gene.symbol*** | **Gene.title** | **P.Value** | **logFC** |
| *HECW2* | HECT, C2 and WW domain containing E3 ubiquitin protein ligase 2 | 0.0001706 | 3.8996927 |
| *IFIT2* | interferon induced protein with tetratricopeptide repeats 2 | 0.0003654 | 2.4453314 |
| *MAP1A* | microtubule associated protein 1A | 0.0005462 | 2.2502438 |
| *DCSTAMP* | dendrocyte expressed seven transmembrane protein | 0.00069 | 2.0557978 |
| *DBP* | D-box binding PAR bZIP transcription factor | 0.0007091 | 2.3411806 |
| *KCNMA1* | potassium calcium-activated channel subfamily M alpha 1 | 0.000775 | 2.6883105 |
| *BMF* | Bcl2 modifying factor | 0.0008293 | 2.3812823 |
| *AK5* | adenylate kinase 5 | 0.0009446 | 2.0735677 |
| *RGS4* | regulator of G-protein signaling 4 | 0.0013752 | 2.0851538 |
| *CCDC85A* | coiled-coil domain containing 85A | 0.0015258 | 2.3139416 |
| *FAM46B* | family with sequence similarity 46 member B | 0.0016123 | 2.283166 |
| *ADD2* | adducin 2 | 0.0019393 | 2.2657107 |
| *ELF3* | E74 like ETS transcription factor 3 | 0.0019608 | 2.0229184 |
| *ACTG2* | actin, gamma 2, smooth muscle, enteric | 0.0019921 | 2.0427189 |
| *MRGPRF* | MAS related GPR family member F | 0.0019927 | 2.03373 |
| *LINC00525* | long intergenic non-protein coding RNA 525 | 0.0023212 | 2.2356756 |
| *IGHM* | immunoglobulin heavy constant mu | 0.0024047 | 4.2072312 |
| *CA12* | carbonic anhydrase 12 | 0.0024738 | 2.4603544 |
| *TRIM54* | tripartite motif containing 54 | 0.0029048 | 2.1911005 |
| *HCG26* | HLA complex group 26 (non-protein coding) | 0.0042559 | 2.1643547 |
| *HMOX1* | heme oxygenase 1 | 0.0043398 | 2.4590538 |
| *CABP7* | calcium binding protein 7 | 0.004531 | 2.0882456 |
| *NFE2L1* | nuclear factor, erythroid 2 like 1 | 0.0045358 | 2.2081537 |
| *FGB* | fibrinogen beta chain | 0.0045397 | 2.0399371 |
| *SLC12A3* | solute carrier family 12 member 3 | 0.0047162 | 2.4306461 |
| *DLGAP1* | DLG associated protein 1 | 0.0069051 | 2.2863047 |
| *ZAP70* | zeta chain of T cell receptor associated protein kinase 70 | 0.0082232 | 3.2724198 |
| *FLJ37035* | uncharacterized LOC399821 | 0.0082354 | 2.0616388 |
| *EDN2* | endothelin 2 | 0.0095325 | 4.2599616 |
| *FXYD2* | FXYD domain containing ion transport regulator 2 | 4.83E-06 | -9.669387 |
| *HIST1H4A* | histone cluster 1, H4a | 0.0000049 | -9.139027 |
| *HIST2H4B* | histone cluster 2, H4b | 0.000005 | -6.904199 |
| *HIST1H4H* | histone cluster 1, H4h | 5.14E-06 | -7.293217 |
| *HIST1H4E* | histone cluster 1, H4e | 0.0000053 | -7.078397 |
| *HIST1H4I* | histone cluster 1, H4i | 5.54E-06 | -7.018387 |
| *HIST1H4F* | histone cluster 1, H4f | 6.22E-06 | -7.105433 |
| *HIST1H2AM* | histone cluster 1, H2am | 7.26E-06 | -6.607269 |
| *HIST1H4J* | histone cluster 1, H4j | 7.31E-06 | -6.54296 |
| *HIST1H4K* | histone cluster 1, H4k | 7.77E-06 | -6.393458 |
| *HIST1H3B* | histone cluster 1, H3b | 1.064E-05 | -5.777427 |
| *HIST1H2AL* | histone cluster 1, H2al | 1.858E-05 | -5.884604 |
|  |  | 0.0000199 | -5.672077 |
| *HIST1H3H* | histone cluster 1, H3h | 2.331E-05 | -5.117504 |
| *SCARNA17* | small Cajal body-specific RNA 17 | 2.485E-05 | -4.808048 |
| *HIST1H4G* | histone cluster 1, H4g | 3.086E-05 | -5.762986 |
| *TMEM107* | transmembrane protein 107 | 4.365E-05 | -4.027302 |
| *HIST1H2AB* | histone cluster 1, H2ab | 4.403E-05 | -4.744968 |
| *HIST1H2AD* | histone cluster 1, H2ad | 5.154E-05 | -4.166069 |
| *EGR1* | early growth response 1 | 5.695E-05 | -7.041513 |
| *HIST1H2AJ* | histone cluster 1, H2aj | 5.891E-05 | -3.874649 |
| *HIST1H2AE* | histone cluster 1, H2ae | 7.447E-05 | -3.699938 |
| *KLF15* | Kruppel like factor 15 | 7.638E-05 | -5.433818 |
| *HIST1H2BJ* | histone cluster 1, H2bj | 7.752E-05 | -3.776725 |
| *HIST4H4* | histone cluster 4, H4 | 7.826E-05 | -6.94075 |
| *EIF4A2* | eukaryotic translation initiation factor 4A2 | 9.983E-05 | -3.45305 |
| *RCOR2* | REST corepressor 2 | 0.0001125 | -5.616595 |
| *DNAJA4* | DnaJ heat shock protein family (Hsp40) member A4 | 0.0001185 | -3.155849 |
| *HIST2H2AB* | histone cluster 2, H2ab | 0.0001214 | -3.214307 |
| *HIST2H2AC* | histone cluster 2, H2ac | 0.0001416 | -3.918584 |
| *SLC6A4* | solute carrier family 6 member 4 | 0.0001467 | -3.520443 |
| *SNHG25///SNORA50C* | small nucleolar RNA host gene 25///small nucleolar RNA, H/ACA box 50C | 0.0001484 | -4.112046 |
| *HIST2H3D* | histone cluster 2, H3d | 0.0001492 | -3.600021 |
| *HIST1H3D* | histone cluster 1, H3d | 0.0001563 | -3.684603 |
| *HIST1H2BD* | histone cluster 1, H2bd | 0.0001687 | -2.957532 |
| *LOC730961* | uncharacterized LOC730961 | 0.0001733 | -2.895087 |
| *HIST3H2A* | histone cluster 3, H2a | 0.000183 | -3.429953 |
| *HIST1H2BO* | histone cluster 1, H2bo | 0.0001886 | -3.236677 |
| *HIST1H2AH* | histone cluster 1, H2ah | 0.0001906 | -2.86578 |
| *HIST2H3A* | histone cluster 2, H3a | 0.0001921 | -3.160409 |
| *HIST1H2BE* | histone cluster 1, H2be | 0.0001995 | -3.203547 |
| *HIST1H2BF* | histone cluster 1, H2bf | 0.0002366 | -3.167682 |
| *HIST1H2BH* | histone cluster 1, H2bh | 0.0002413 | -2.916446 |
| *HIST1H2BB* | histone cluster 1, H2bb | 0.0002526 | -2.841501 |
| *HIST1H2BL* | histone cluster 1, H2bl | 0.0002738 | -2.883909 |
| *MYC* | v-myc avian myelocytomatosis viral oncogene homolog | 0.0002762 | -3.09926 |
| *HIST3H2BB* | histone cluster 3, H2bb | 0.0002851 | -3.020559 |
| *HIST1H1B* | histone cluster 1, H1b | 0.0002978 | -2.881473 |
| *HIST1H2AK* | histone cluster 1, H2ak | 0.0002986 | -2.939189 |
| *HIST2H2BE* | histone cluster 2, H2be | 0.0003326 | -2.672971 |
| *HIST2H2AA4* | histone cluster 2, H2aa4 | 0.0003344 | -2.686141 |
| *SPRY2* | sprouty RTK signaling antagonist 2 | 0.0003465 | -2.794152 |
| *HIST1H2AG* | histone cluster 1, H2ag | 0.0003583 | -2.665647 |
| *HIST1H2BI* | histone cluster 1, H2bi | 0.0003637 | -2.699502 |
| *FOS* | Fos proto-oncogene, AP-1 transcription factor subunit | 0.0003715 | -2.666138 |
| *HIST2H2BD* | histone cluster 2, H2bd (pseudogene) | 0.0003925 | -2.645206 |
| *ANKRD30BP2* | ankyrin repeat domain 30B pseudogene 2 | 0.0004168 | -2.47919 |
| *SPRED2* | sprouty related EVH1 domain containing 2 | 0.00044 | -3.022323 |
| *HIST1H1D* | histone cluster 1, H1d | 0.0004883 | -2.365058 |
| *RASL11B* | RAS like family 11 member B | 0.0004955 | -2.237697 |
| *HIST1H3F* | histone cluster 1, H3f | 0.0005013 | -4.131532 |
| *LINC00654* | long intergenic non-protein coding RNA 654 | 0.0005071 | -2.349178 |
| *PTPN4* | protein tyrosine phosphatase, non-receptor type 4 | 0.0005421 | -2.324155 |
| *PIANP* | PILR alpha associated neural protein | 0.0005798 | -4.112262 |
| *YOD1* | YOD1 deubiquitinase | 0.0006028 | -2.169654 |
| *HIST1H2BN* | histone cluster 1, H2bn | 0.0006071 | -2.346743 |
| *CEP83-AS1* | CEP83 antisense RNA 1 (head to head) | 0.0006548 | -2.751812 |
| *HIST1H2BG* | histone cluster 1, H2bg | 0.0006824 | -2.348824 |
| *HIST1H2BC* | histone cluster 1, H2bc | 0.000693 | -2.119722 |
| *AREG* | amphiregulin | 0.0007706 | -2.088678 |
| *MIA* | melanoma inhibitory activity | 0.0008002 | -2.09762 |
| *MAFF* | MAF bZIP transcription factor F | 0.0008177 | -2.908953 |
| *EIF1AY* | eukaryotic translation initiation factor 1A, Y-linked | 0.0008258 | -3.657671 |
| *HIST1H2BM* | histone cluster 1, H2bm | 0.0008292 | -2.19199 |
| *HIST1H3G* | histone cluster 1, H3g | 0.0009474 | -2.073936 |
| *PIF1* | PIF1 5'-to-3' DNA helicase | 0.0010104 | -2.072199 |
| *PARGP1* | poly(ADP-ribose) glycohydrolase pseudogene 1 | 0.0010586 | -2.158406 |
| *CMTM7* | CKLF like MARVEL transmembrane domain containing 7 | 0.0010828 | -2.134863 |
| *ERN1* | endoplasmic reticulum to nucleus signaling 1 | 0.0010971 | -2.624894 |
| *CCL3* | C-C motif chemokine ligand 3 | 0.0011134 | -2.399822 |
| *DRD5* | dopamine receptor D5 | 0.0012065 | -2.060972 |
| *HIST1H4B* | histone cluster 1, H4b | 0.0012379 | -2.2291 |
| *DYRK1A* | dual specificity tyrosine phosphorylation regulated kinase 1A | 0.0012593 | -3.068713 |
| *IL12A* | interleukin 12A | 0.001334 | -3.401465 |
| *LIF* | leukemia inhibitory factor | 0.0013559 | -2.322913 |
| *RNF186* | ring finger protein 186 | 0.0014088 | -3.550537 |
| *ARRDC3* | arrestin domain containing 3 | 0.0014439 | -2.712382 |
| *MUC7* | mucin 7, secreted | 0.0014816 | -3.355228 |
| *FRZB* | frizzled-related protein | 0.0015864 | -2.006044 |
| *ABL2* | ABL proto-oncogene 2, non-receptor tyrosine kinase | 0.0016691 | -2.104716 |
| *OR2H2* | olfactory receptor family 2 subfamily H member 2 | 0.0016696 | -2.702801 |
| *PLEKHH1* | pleckstrin homology, MyTH4 and FERM domain containing H1 | 0.0016874 | -2.176879 |
| *ARC* | activity regulated cytoskeleton associated protein | 0.0017303 | -2.185611 |
| *PPP1R37* | protein phosphatase 1 regulatory subunit 37 | 0.0017539 | -3.327237 |
| *GIPR* | gastric inhibitory polypeptide receptor | 0.0017717 | -3.143466 |
| *NKPD1* | NTPase, KAP family P-loop domain containing 1 | 0.001796 | -2.406352 |
| *SIRT6* | sirtuin 6 | 0.0018011 | -4.033505 |
| *MFSD2A* | major facilitator superfamily domain containing 2A | 0.0019002 | -2.377213 |
| *EPB41L4B* | erythrocyte membrane protein band 4.1 like 4B | 0.0020151 | -2.022274 |
| *MGEA5* | meningioma expressed antigen 5 (hyaluronidase) | 0.0020208 | -2.00679 |
| *DUSP6* | dual specificity phosphatase 6 | 0.0020758 | -2.482942 |
| *SPRY4* | sprouty RTK signaling antagonist 4 | 0.0021448 | -3.62344 |
| *DNAJB3* | DnaJ heat shock protein family (Hsp40) member B3 | 0.0021539 | -2.947806 |
| *BMP15* | bone morphogenetic protein 15 | 0.0023563 | -4.806798 |
| *FGF7* | fibroblast growth factor 7 | 0.0025074 | -2.890176 |
| *HS3ST1* | heparan sulfate-glucosamine 3-sulfotransferase 1 | 0.0025482 | -2.266536 |
| *SPRY1* | sprouty RTK signaling antagonist 1 | 0.0031074 | -2.497068 |
| *MGC57346* | ADP-ribosylation factor pseudogene | 0.0031158 | -2.018877 |
| *HIST1H2AA* | histone cluster 1, H2aa | 0.0032677 | -2.810261 |
| *LOC100286922* | DnaJ heat shock protein family (Hsp40) member B3 pseudogene | 0.0033254 | -2.379742 |
| *HLA-DOA* | major histocompatibility complex, class II, DO alpha | 0.0034757 | -2.651585 |
| *EGR2* | early growth response 2 | 0.0037499 | -2.208291 |
| *KCNH2* | potassium voltage-gated channel subfamily H member 2 | 0.0039537 | -2.205977 |
| *EGR4* | early growth response 4 | 0.0043474 | -2.380637 |
| *PAX4* | paired box 4 | 0.0044303 | -3.004821 |
| *MTTP* | microsomal triglyceride transfer protein | 0.0047264 | -3.202093 |
| *CTXN3* | cortexin 3 | 0.0048435 | -4.560873 |
| *DUOX1* | dual oxidase 1 | 0.0055581 | -2.305815 |
| *ZNF543* | zinc finger protein 543 | 0.0056603 | -2.258713 |
| *GPR21* | G protein-coupled receptor 21 | 0.0056612 | -2.420475 |
| *ZC3H7B* | zinc finger CCCH-type containing 7B | 0.0057777 | -2.395308 |
| *IGKC* | immunoglobulin kappa constant | 0.0063548 | -2.796252 |
| *TOX3* | TOX high mobility group box family member 3 | 0.006567 | -3.046032 |
| *MEP1A* | meprin A subunit alpha | 0.0066058 | -2.127729 |
| *C20orf173* | chromosome 20 open reading frame 173 | 0.0071019 | -2.114166 |
| *LOC283861* | uncharacterized LOC283861 | 0.0081794 | -2.2209 |
| *SF1* | splicing factor 1 | 0.0081901 | -2.775106 |
| *SPPL3* | signal peptide peptidase like 3 | 0.008207 | -2.194969 |
| *KCNH6* | potassium voltage-gated channel subfamily H member 6 | 0.0088722 | -2.053727 |
| *ADGRG3* | adhesion G protein-coupled receptor G3 | 0.0091263 | -2.986021 |
| *MBNL1* | muscleblind like splicing regulator 1 | 0.0092605 | -2.017427 |
| *IGHV3-30* | immunoglobulin heavy variable 3-30 | 0.0094009 | -2.741247 |
| *PPP2R2D* | protein phosphatase 2 regulatory subunit Bdelta | 0.0096014 | -2.077771 |
| *FILIP1L* | filamin A interacting protein 1 like | 0.0099506 | -2.047367 |
| *Mock vs Monkeypox-infected (Macaca mulatta) - Total of 50* | | | |
| ***Gene.symbol*** | **Gene.title** | **P.Value** | **logFC** |
| *LOC695323* | histone H4 | 4.44E-09 | 1.53 |
| *PMAIP1* | phorbol-12-myristate-13-acetate-induced protein 1 | 5.98E-09 | 1.63 |
| *LOC695427* | histone H3.1 | 2.42E-08 | 2.62 |
| *TMEM107* | transmembrane protein 107 | 2.67E-08 | 3.83 |
| *HIST1H2AD* | histone cluster 1, H2ad | 2.87E-08 | 2.24 |
| *LOC695891* | histone H3.1-like | 1.51E-07 | 1.86 |
| *HIST1H2BJ* | histone cluster 1, H2bj | 1.62E-07 | 1.67 |
| *LOC705352* | histone H4-like | 2.00E-07 | 1.45 |
| *LOC695214* | histone H4-like | 2.02E-07 | 2.55 |
| *LOC698731* | histone H3.2-like | 2.30E-07 | 1.58 |
| *HIST1H3D* | histone cluster 1, H3d | 2.47E-07 | 1.62 |
| *LOC697721* | histone H4 | 7.43E-07 | 1.53 |
| *LOC704994* | histone H2A type 1-H-like | 8.73E-07 | 1.34 |
| *HIST1H1D* | histone cluster 1, H1d | 1.95E-06 | 1.22 |
| *LOC705220* | histone H3.1t-like | 2.69E-06 | 1.24 |
| *BIRC3* | baculoviral IAP repeat containing 3 | 4.26E-06 | 2.3 |
| *LOC697857* | histone H2B type 1 | 1.13E-05 | 1.3 |
| *CCL2* | chemokine (C-C motif) ligand 2 | 4.88E-05 | 1.81 |
| *HIST2H2AC* | histone cluster 2, H2ac | 6.71E-05 | 1.15 |
| *INHBA* | inhibin beta A | 8.67E-05 | 1.37 |
| *VCAM1* | vascular cell adhesion molecule 1 | 1.17E-04 | 1.52 |
| *LOC714885* | hypothetical protein LOC714885 | 1.38E-04 | 1.96 |
| *CXCL8* | chemokine (C-X-C motif) ligand 8 | 2.78E-04 | 7.78 |
| *IL6* | interleukin 6 | 6.21E-04 | 3.42 |
| *TNFAIP3* | TNF alpha induced protein 3 | 9.34E-04 | 3.95 |
| *SRP68* | signal recognition particle 68kDa | 1.01E-03 | 3.8 |
| *HOXA9* | homeobox A9 | 1.06E-10 | -1.18 |
| *AJUBA* | ajuba LIM protein | 1.49E-09 | -1.34 |
| *LOC714008* | 39S ribosomal protein L2, mitochondrial pseudogene | 2.91E-09 | -1.61 |
| *CD24* | CD24 molecule | 1.15E-08 | -1.19 |
| *PAX6* | paired box 6 | 1.54E-08 | -1.51 |
| *NCOA2* | nuclear receptor coactivator 2 | 3.11E-08 | -1.03 |
| *SOX4* | SRY-box 4 | 5.44E-08 | -1.04 |
| *BAMBI* | BMP and activin membrane-bound inhibitor | 5.64E-08 | -1.11 |
| *HOXA10* | homeobox A10 | 6.09E-08 | -1.18 |
| *ZMYND8* | zinc finger, MYND-type containing 8 | 7.51E-08 | -1.04 |
| *SLC7A11* | solute carrier family 7 (anionic amino acid transporter light chain, xc- system), member 11 | 8.49E-08 | -1.14 |
| *CREBRF* | CREB3 regulatory factor | 2.47E-07 | -1.49 |
| *SIAH1* | siah E3 ubiquitin protein ligase 1 | 4.05E-07 | -1.01 |
| *CEBPD* | CCAAT/enhancer binding protein (C/EBP), delta | 6.59E-07 | -1.62 |
| *DDIT4* | DNA damage inducible transcript 4 | 6.70E-07 | -1.9 |
| *RRAGC* | Ras-related GTP binding C | 6.94E-07 | -1.01 |
| *TMEM60* | transmembrane protein 60 | 9.98E-07 | -1.16 |
| *JDP2* | Jun dimerization protein 2 | 1.54E-06 | -1.23 |
| *HBP1* | HMG-box transcription factor 1 | 2.02E-06 | -1.52 |
| *TRIB3* | tribbles pseudokinase 3 | 6.09E-06 | -1.05 |
| *DDIT3* | DNA damage inducible transcript 3 | 1.55E-05 | -1.08 |
| *CEBPB* | CCAAT/enhancer binding protein (C/EBP), beta | 2.52E-05 | -1.08 |
| *CITED2* | Cbp/p300-interacting transactivator, with Glu/Asp rich carboxy-terminal domain, 2 | 2.83E-04 | -1.95 |
| *PTPN13* | protein tyrosine phosphatase, non-receptor type 13 (APO-1/CD95 (Fas)-associated phosphatase) | 2.84E-04 | -1.16 |

**Supplementary Table S3:** Screening of Viral Proteins from the Dataset (GSE11234)

| Differential Viral proteins of Mock vs MPX(Primary Human Macrophage)-183 viral proteins | | |
| --- | --- | --- |
| SPOT_ID | P.Value | logFC |
| C1L | 1.38E-12 | 6.5794 |
| O2L | 2.27E-12 | 7.20185 |
| F4L | 2.40E-12 | 7.5318625 |
| H5R | 4.38E-12 | 7.193725 |
| A51R | 7.16E-12 | 6.5487875 |
| L5L | 1.47E-11 | 7.0731125 |
| D6L | 1.66E-11 | 5.81145 |
| E4R | 1.73E-11 | 5.5285875 |
| D10L | 1.81E-11 | 6.2325875 |
| M3L | 2.26E-11 | 4.7071125 |
| A49R | 3.22E-11 | 6.0554625 |
| M2R | 4.48E-11 | 5.7891125 |
| B13R | 4.76E-11 | 6.108275 |
| A45L | 5.31E-11 | 5.1138 |
| F3L | 7.29E-11 | 5.7665375 |
| L2R | 7.93E-11 | 5.4866 |
| J2R | 8.92E-11 | 5.3038 |
| B3R | 1.31E-10 | 5.73475 |
| B12R | 1.36E-10 | 6.5163875 |
| A10L | 1.43E-10 | 5.94785 |
| C5L | 1.43E-10 | 6.2635 |
| D3R | 1.68E-10 | 5.94375 |
| A33R | 1.76E-10 | 5.9220125 |
| I3L | 1.79E-10 | 7.1931 |
| A39R | 1.87E-10 | 4.779275 |
| D8L | 2.31E-10 | 5.7124 |
| B20R | 2.54E-10 | 7.60105 |
| P1L | 3.59E-10 | 5.7260875 |
| C21L | 3.74E-10 | 5.2732875 |
| B9R | 3.79E-10 | 7.011975 |
| D19L | 4.84E-10 | 6.094725 |
| P2L | 6.38E-10 | 5.2001625 |
| B10R | 7.32E-10 | 5.8504625 |
| C8L | 8.54E-10 | 6.7817375 |
| A9R | 8.94E-10 | 5.1324 |
| B4R | 9.63E-10 | 5.46335 |
| F7R | 1.24E-09 | 5.8357875 |
| J3R | 1.44E-09 | 6.0579875 |
| E9R | 1.44E-09 | 4.0801875 |
| C7L | 1.44E-09 | 4.71275 |
| D5R | 2.05E-09 | 4.76435 |
| A35R | 2.20E-09 | 5.7153125 |
| D12L | 2.70E-09 | 5.518825 |
| A31L | 2.85E-09 | 5.104525 |
| N1R | 4.07E-09 | 4.6755375 |
| B17R | 4.20E-09 | 4.075425 |
| G6R | 4.25E-09 | 4.5663 |
| J2L | 4.69E-09 | 5.11395 |
| A44R | 4.77E-09 | 4.57405 |
| F8L | 5.19E-09 | 5.485725 |
| I2L | 6.12E-09 | 5.4706125 |
| A7L | 6.64E-09 | 5.3607 |
| D18L | 6.77E-09 | 5.0961 |
| L3R | 1.02E-08 | 4.674525 |
| C2L | 1.17E-08 | 4.46585 |
| J1L | 1.22E-08 | 4.7468 |
| A48R | 1.27E-08 | 3.34895 |
| A47R | 1.38E-08 | 4.6858625 |
| G2L | 2.88E-08 | 3.671675 |
| D17L | 3.17E-08 | 4.6091625 |
| E8R_1_a | 3.31E-08 | 5.911575 |
| D13L | 3.35E-08 | 4.20415 |
| C20L | 3.62E-08 | 4.4281875 |
| C18L | 4.24E-08 | 4.08295 |
| N4R | 4.37E-08 | 4.8755875 |
| I4L | 4.88E-08 | 4.262675 |
| A6R | 5.62E-08 | 4.009975 |
| L6R | 5.88E-08 | 4.05165 |
| E12L | 7.32E-08 | 4.8545375 |
| A24R | 7.72E-08 | 3.8840875 |
| A37R | 8.08E-08 | 6.3844625 |
| A41L | 8.28E-08 | 4.6459375 |
| B19R | 1.22E-07 | 3.80875 |
| R1R | 1.36E-07 | 3.1382 |
| A49L_40_s | 2.20E-07 | 4.5405375 |
| B6R | 2.46E-07 | 3.6466375 |
| C14L | 2.72E-07 | 3.2676125 |
| Q2L | 2.77E-07 | 4.9487125 |
| A22R | 2.78E-07 | 3.74545 |
| G5R | 3.16E-07 | 3.2866 |
| A19R | 3.49E-07 | 3.9385875 |
| E1R | 4.69E-07 | 4.3311125 |
| E2L | 4.99E-07 | 2.77385 |
| A34L | 7.37E-07 | 2.895075 |
| D9L | 7.57E-07 | 2.776625 |
| D1L | 8.91E-07 | 4.4591125 |
| E5R | 1.03E-06 | 3.6059 |
| M5R | 1.04E-06 | 3.9398125 |
| C11L | 1.35E-06 | 3.4195 |
| B2R | 1.55E-06 | 3.0514375 |
| E8L | 1.72E-06 | 3.557325 |
| A28L | 1.81E-06 | 2.9353375 |
| D4L | 1.90E-06 | 2.68515 |
| A4L_567_s | 1.93E-06 | 3.449025 |
| B21R | 2.69E-06 | 3.237875 |
| B14R | 3.00E-06 | 2.9803875 |
| B8R | 3.07E-06 | 3.238475 |
| I1L | 3.63E-06 | 4.1360625 |
| F6R | 3.78E-06 | 4.396625 |
| B7R_657_a | 4.04E-06 | 3.2708875 |
| A1L | 4.57E-06 | 3.352075 |
| D16L | 4.68E-06 | 3.1665875 |
| D15L | 4.83E-06 | 2.9218125 |
| A50R | 5.82E-06 | 3.1161 |
| A5L | 6.60E-06 | 3.3064125 |
| M1R | 6.66E-06 | 3.0182875 |
| H7R | 7.20E-06 | 3.3352625 |
| A42R | 7.35E-06 | 2.5552875 |
| A16L | 8.89E-06 | 3.25635 |
| A3L | 9.13E-06 | 3.3826 |
| A4L | 9.93E-06 | 3.7387375 |
| A11L | 1.18E-05 | 3.084575 |
| H6R | 1.24E-05 | 3.2815 |
| A29L | 1.27E-05 | 3.6734 |
| A12R | 1.30E-05 | 3.2766875 |
| A40L | 2.12E-05 | 2.8934625 |
| A13L | 2.36E-05 | 3.852525 |
| C19L | 2.44E-05 | 3.4704125 |
| C6R | 2.53E-05 | 2.4654375 |
| E13L | 2.58E-05 | 2.684775 |
| E6R | 2.91E-05 | 3.1603375 |
| A15L | 3.21E-05 | 4.418825 |
| L1R | 3.49E-05 | 2.9873375 |
| A30L | 3.76E-05 | 2.7196875 |
| A17L | 4.27E-05 | 3.64595 |
| C9L | 4.74E-05 | 2.1499125 |
| H2R | 4.78E-05 | 2.7764125 |
| J1R | 4.89E-05 | 2.2533875 |
| L6R_3484_a | 4.95E-05 | 2.7616875 |
| H6R_295_a | 5.17E-05 | 2.581675 |
| A8R_0_a | 5.34E-05 | 2.5614625 |
| Q1L_1269_a | 5.49E-05 | 2.2811375 |
| A26L | 6.06E-05 | 2.289275 |
| E11L | 6.40E-05 | 2.2896125 |
| E10R | 6.50E-05 | 2.2520625 |
| C4L | 7.70E-05 | 2.384275 |
| B5R | 9.28E-05 | 2.7623125 |
| A21L | 1.26E-04 | 2.3558 |
| A20L | 1.39E-04 | 2.6131875 |
| G9R | 1.65E-04 | 2.945875 |
| A27L | 1.91E-04 | 2.24905 |
| J3L | 2.11E-04 | 2.0040625 |
| A46R | 2.32E-04 | 2.1135 |
| I6L | 2.46E-04 | 2.1808 |
| A43R | 2.49E-04 | 2.210875 |
| A32L | 2.83E-04 | 2.1476625 |
| E7R_92_a | 3.17E-04 | 2.716375 |
| A20L_29_a | 3.66E-04 | 2.128775 |
| F10R_106_a | 5.42E-04 | 2.402175 |
| M4R | 5.66E-04 | 2.53305 |
| A17L_370_s | 6.24E-04 | 3.0813875 |
| H3L | 7.17E-04 | 2.143425 |
| F9R_94_s | 9.01E-04 | 2.513975 |
| A28L_1230_s | 1.02E-03 | 2.0828375 |
| G4L | 1.07E-03 | 2.1991375 |
| E3R | 1.69E-03 | 2.164425 |
| A14L | 2.36E-03 | 2.5293375 |
| C17L | 1.68E-11 | 7.22 |
|  | 1.12E-10 | 6.03 |
| Q1L | 9.72E-10 | 5.1 |
| B11R | 1.27E-09 | 5.03 |
| D11L | 1.36E-09 | 5.8 |
| C10L | 4.95E-09 | 7.78 |
| B16R | 7.14E-09 | 7.04 |
| L4R | 2.46E-08 | 5.51 |
| A38R | 2.55E-08 | 6.05 |
| F2L | 5.50E-07 | 3 |
| C13L | 5.95E-07 | 4.1 |
| D2L | 1.06E-06 | 3.53 |
| D7L | 1.34E-06 | 3.52 |
| F10L | 4.42E-06 | 3.43 |
| E7R | 6.20E-06 | 4.69 |
| C22L | 7.70E-06 | 4.45 |
| O1L | 1.34E-05 | 3.89 |
| B7R | 2.40E-05 | 3.25 |
| N3R | 7.31E-05 | 3.82 |
| G7R | 7.51E-05 | 3.02 |
| G3R | 2.44E-04 | 2.63 |
| C12L | 6.24E-04 | 2.05 |
| A25R | 8.68E-04 | 3.45 |
| D14L | 1.15E-03 | 2.47 |
| F1L | 1.71E-03 | 2.31 |
| EMPTY | 2.39E-03 | 4.87 |
| C23R | 6.10E-03 | 2.22 |
| Differential viral proteins -Hela -128 | | |
| SPOT_ID | P.Value | logFC |
| F4L | 0.00000207 | 7.47 |
| C1L | 0.00000282 | 6.96 |
| B16R | 0.00000292 | 8.15 |
| H5R | 0.00000371 | 7.9 |
| D10L | 0.00000544 | 6.66 |
| A51R | 0.00000548 | 7.79 |
| C5L | 0.00000811 | 5.34 |
| L2R | 0.00000878 | 7.77 |
| B10R | 0.00000943 | 6.4 |
| A39R | 0.00001042 | 6.85 |
| B4R | 0.00001061 | 7.36 |
| N4R | 0.00001063 | 6.5 |
| I3L | 0.00001414 | 7.92 |
| A37R | 0.00001466 | 6.45 |
| A33R | 0.00001491 | 7.39 |
| C10L | 0.00001504 | 7.87 |
| I2L | 0.00001539 | 6.91 |
| A47R | 0.00001627 | 5.7 |
| D3R | 0.00001701 | 6.71 |
| C17L | 0.00001714 | 7.18 |
| D1L | 0.00002165 | 6.83 |
| A7L | 0.00002548 | 6.47 |
| A23R | 0.00002597 | 4.92 |
| O2L | 0.00002836 | 6.72 |
| D8L | 0.00003195 | 6.31 |
| I4L | 0.00003228 | 5.58 |
| C7L | 0.0000334 | 6.4 |
| Q1L | 0.00003371 | 5.73 |
| B9R | 0.00004383 | 6.91 |
| B21R | 0.00004693 | 5.32 |
| A38R | 0.00005341 | 6.11 |
| A45L | 0.00005556 | 5.51 |
| B12R | 0.00006549 | 6.05 |
| B13R | 0.0000659 | 6.46 |
| B20R | 0.00006623 | 6.42 |
| M2R | 0.00006745 | 4.96 |
| C21L | 0.00006874 | 6.37 |
| G6R | 0.00006894 | 5.89 |
| L5L | 0.00006997 | 6.14 |
| A25R | 0.0000705 | 5.48 |
| C13L | 0.00007218 | 4.55 |
| A44R | 0.00007504 | 5.95 |
| L4R | 0.00007685 | 6.59 |
| B11R | 0.00008405 | 5.51 |
| J3R | 0.00009066 | 7.05 |
| J1L | 0.00011883 | 6.3 |
| F3L | 0.00012101 | 5.37 |
| B3R | 0.00012375 | 5.74 |
| G7R | 0.00012514 | 5.76 |
| A35R | 0.00012521 | 4.59 |
| A41L | 0.00012563 | 5.92 |
| E1R | 0.00013197 | 6.18 |
| C20L | 0.0001325 | 4.02 |
| P2L | 0.00014041 | 6.18 |
| D19L | 0.00014046 | 6.5 |
| A10L | 0.00015574 | 5.85 |
| B6R | 0.00015978 | 4.98 |
| M3L | 0.00018064 | 5.43 |
| P1L | 0.00019239 | 6.04 |
| E5R | 0.00020246 | 6.04 |
| A48R | 0.00020605 | 4.57 |
| E12L | 0.00020782 | 6.51 |
| D7L | 0.00021428 | 5.14 |
| L6R | 0.00021959 | 5.37 |
| D5R | 0.00025013 | 5.36 |
| A9R | 0.00025088 | 5.89 |
| J2L | 0.00029118 | 5.23 |
| N3R | 0.00029875 | 5.21 |
| C18L | 0.00035436 | 4.69 |
| A34L | 0.00036191 | 4.84 |
| A31L | 0.00038348 | 6.67 |
| D4L | 0.00038953 | 2.54 |
| A20L | 0.00039199 | 5.88 |
| A49R | 0.00039289 | 5.35 |
| C22L | 0.00039651 | 5.14 |
| B19R | 0.00044686 | 4.03 |
| D2L | 0.00046248 | 3.86 |
| C4L | 0.00050146 | 4.16 |
| A6R | 0.00050623 | 5.34 |
| C2L | 0.00051437 | 5.91 |
| D12L | 0.00057646 | 5.17 |
| F1L | 0.00057789 | 5.01 |
| C6R | 0.00060176 | 5.2 |
| M1R | 0.00062573 | 4.35 |
| B2R | 0.00069779 | 4.87 |
| C19L | 0.0008081 | 5.26 |
| E10R | 0.0008568 | 3.71 |
| C8L | 0.00088577 | 6.23 |
| D6L | 0.00090485 | 5.5 |
| B7R | 0.00092029 | 4.54 |
| E7R | 0.00095875 | 4.57 |
| A1L | 0.00098055 | 5.66 |
| F10L | 0.0009841 | 5.02 |
| A22R | 0.00103301 | 3.35 |
| F6R | 0.00117968 | 6.35 |
| F7R | 0.00120568 | 5.77 |
| A19R | 0.00133645 | 4.47 |
| D15L | 0.00133717 | 4.27 |
| A40L | 0.00141007 | 4.54 |
| H7R | 0.00156457 | 4.6 |
| E8L | 0.00160311 | 4.04 |
| Q2L | 0.00164067 | 5.56 |
| A21L | 0.00175492 | 3.89 |
| D18L | 0.00202477 | 5.54 |
| I1L | 0.00221013 | 5.73 |
| C11L | 0.00246595 | 3.94 |
| E6R | 0.00257426 | 3.7 |
| J2R | 0.00285433 | 3.37 |
| A3L | 0.00341014 | 5.12 |
| J3L | 0.0034745 | 3.53 |
| F5R | 0.00555254 | 3.2 |
| H1L | 0.00585807 | 4.77 |
| C12L | 0.00598545 | 3.39 |
| A2L | 0.00632518 | 5.04 |
| L1R | 0.00665369 | 4.18 |
| B17R | 0.00683932 | 2.93 |
| A27L | 0.00766622 | 4.02 |
| D13L | 0.0078092 | 4.32 |
| F2L | 0.00804425 | 2.98 |
| A46R | 0.00809217 | 4.23 |
| E2L | 0.00813373 | 3.43 |
| B18R | 0.00822992 | 2.46 |
| A50R | 0.00850941 | 3.59 |
| A36R | 0.00851895 | 3.16 |
| A28L | 0.008561 | 3.93 |
| A13L | 0.00860639 | 5.16 |
| A42R | 0.00907098 | 3.77 |
| M5R | 0.00971551 | 2.93 |
| Differential viral proteins -Primary Human Fibroblast -313 | | |
| SPOT_ID | P.Value | logFC |
| LC:17996 | 9.61E-08 | 2.46 |
| LCP:63 | 3.80E-07 | 2.46 |
| C8L | 5.31E-06 | 8.07 |
| C17L | 1.53E-05 | 7.61 |
| A39R | 1.53E-05 | 7.11 |
| J6R_73_a | 1.56E-05 | 4.58 |
| D14L_464_s | 1.85E-05 | 7.1 |
| C7L | 1.97E-05 | 5.5 |
| B13R | 1.98E-05 | 6.81 |
| F3L | 2.53E-05 | 5.68 |
| LC:17990 | 2.61E-05 | 2.14 |
| D6L | 2.91E-05 | 5.07 |
| I4L | 2.98E-05 | 6.18 |
| P2L | 3.20E-05 | 5.82 |
| G6R | 3.22E-05 | 5.67 |
| G4R_819_a | 3.27E-05 | 6.49 |
| J5R_472_a | 3.47E-05 | 7.65 |
| Q1L | 3.66E-05 | 6.78 |
| L5L | 3.81E-05 | 6.77 |
| C8L_663_s | 3.81E-05 | 7.35 |
| D7L | 3.88E-05 | 5.53 |
| A48R | 3.88E-05 | 4.15 |
| A51R | 3.92E-05 | 7.16 |
| C19L_140_s | 3.99E-05 | 6.54 |
| C16L | 4.18E-05 | 2.11 |
| C21L | 4.29E-05 | 6.26 |
| I3L | 4.32E-05 | 7.73 |
| E2L | 4.38E-05 | 3.71 |
| J3R | 4.40E-05 | 6.92 |
| A9R | 4.57E-05 | 6.01 |
| A39R_288_a | 4.89E-05 | 6.71 |
| C9L | 5.02E-05 | 3.28 |
| B16R | 5.09E-05 | 7.87 |
| D13L_253_s | 5.17E-05 | 6 |
| C4R_142_a | 5.36E-05 | 3.56 |
| A34R_223_a | 5.88E-05 | 7.13 |
| C10L | 5.91E-05 | 7.5 |
| E5R | 6.01E-05 | 5.18 |
| A44R | 6.19E-05 | 5.15 |
| G5R_348_a | 6.28E-05 | 6.6 |
| A52R_541_a | 6.67E-05 | 7.84 |
| D11L | 6.69E-05 | 6.77 |
| H5_5R_11_a | 6.88E-05 | 6.2 |
| A38R | 6.91E-05 | 6.44 |
| A45L | 7.27E-05 | 5.71 |
| H5R | 8.10E-05 | 7.47 |
| E4L_404_s | 8.39E-05 | 7.32 |
| B10R | 8.52E-05 | 6.5 |
| G7R | 9.12E-05 | 4.86 |
| B9R | 9.65E-05 | 6.92 |
| A35L_163_a | 9.85E-05 | 5.56 |
| C1L | 1.03E-04 | 6.42 |
| N1R | 1.06E-04 | 5.67 |
| F4L | 1.07E-04 | 7.59 |
| J2L | 1.09E-04 | 5.66 |
| D15L | 1.16E-04 | 4.56 |
| E12L | 1.20E-04 | 5.82 |
| B12R | 1.20E-04 | 6.64 |
| B11R | 1.24E-04 | 6.53 |
| F9R_94_a | 1.26E-04 | 2.92 |
| N4R | 1.27E-04 | 6.21 |
| J2R | 1.29E-04 | 5.65 |
| C14L | 1.33E-04 | 3.82 |
| B17R | 1.33E-04 | 4.49 |
| C20L | 1.48E-04 | 5.51 |
| C12L_81_s | 1.50E-04 | 5.34 |
| O2L | 1.50E-04 | 7.19 |
| A33R | 1.56E-04 | 6.68 |
| D10L | 1.59E-04 | 7.07 |
| D2L | 1.64E-04 | 4.69 |
| C5L | 1.66E-04 | 6.42 |
| A24R | 1.75E-04 | 4.69 |
| A49R | 1.76E-04 | 6.33 |
| B4R | 1.81E-04 | 5.83 |
| D8L | 1.90E-04 | 5.8 |
| A46L_108_s | 1.92E-04 | 5.9 |
| C7L_172_s | 1.95E-04 | 5.26 |
| I2L | 1.95E-04 | 6.2 |
| L6R_3484_a | 1.96E-04 | 4.05 |
| O1L | 2.09E-04 | 6.34 |
| J10R_126_a | 2.21E-04 | 4.42 |
| A31L | 2.21E-04 | 5.7 |
| M2R_124_a | 2.33E-04 | 6.52 |
| E7R | 2.34E-04 | 6.18 |
| H5R_572_a | 2.37E-04 | 5.11 |
| A21L | 2.38E-04 | 4.22 |
| P1L | 2.49E-04 | 6.43 |
| J1L | 2.49E-04 | 5.6 |
| M2R | 2.53E-04 | 6.3 |
| D19L | 2.58E-04 | 6.83 |
| C12L | 2.63E-04 | 4.84 |
| D3R | 2.73E-04 | 6.41 |
| A5R_161_a | 2.83E-04 | 3.57 |
| A47R | 2.83E-04 | 5.91 |
| D13_5L_64_s | 2.84E-04 | 5.48 |
| A22R | 3.09E-04 | 4.66 |
| D9L | 3.09E-04 | 4.24 |
| E4R | 3.16E-04 | 5.3 |
| A41L | 3.18E-04 | 5.67 |
| E9R | 3.24E-04 | 4.49 |
| F5R_2046_a | 3.42E-04 | 6.09 |
| O2L_131_a | 3.53E-04 | 2.12 |
| F8L | 3.53E-04 | 5.66 |
| F7R | 3.57E-04 | 6.03 |
| B20R | 3.66E-04 | 7.33 |
| J3R_6_a | 3.70E-04 | 3.64 |
| D5L_20_s | 3.77E-04 | 6.9 |
| M3L | 3.81E-04 | 4.65 |
| A25R | 3.88E-04 | 5.22 |
| B21R | 3.96E-04 | 4.16 |
| D4L | 4.07E-04 | 3.14 |
| N3R | 4.08E-04 | 4.93 |
| D13L | 4.11E-04 | 5.37 |
| B18L_246_s | 4.16E-04 | 3.11 |
| D6L_757_s | 4.26E-04 | 5.34 |
| D17L | 4.43E-04 | 5.53 |
| C9L_977_s | 4.50E-04 | 4.03 |
| B3R | 4.55E-04 | 5.66 |
| C2L | 4.56E-04 | 5.7 |
| I5R_278_a | 4.86E-04 | 7.2 |
| B3L_88_a | 4.98E-04 | 2.79 |
| D16L | 5.19E-04 | 4.84 |
| A9L_98_s | 5.21E-04 | 4 |
| C22L | 5.25E-04 | 6.21 |
| C18L | 5.40E-04 | 5.67 |
| G8L | 5.43E-04 | 3.46 |
| A23R | 5.44E-04 | 3.67 |
| F10R_106_a | 5.46E-04 | 5.47 |
| I6R_182_a | 5.53E-04 | 7.17 |
| L3R | 5.64E-04 | 5.42 |
| D12L_169_s | 5.69E-04 | 4.91 |
| C6L_317_s | 5.69E-04 | 3 |
| A12R | 5.93E-04 | 3.92 |
| A10L | 5.94E-04 | 5.98 |
| J4R_1269_a | 5.99E-04 | 4.82 |
| D18L | 6.12E-04 | 5.63 |
| A43R | 6.24E-04 | 3.85 |
| B20R_776_a | 6.41E-04 | 6.36 |
| A5L | 6.44E-04 | 4.6 |
| A35R | 6.55E-04 | 5.49 |
| B9R_274_a | 6.68E-04 | 6.62 |
| C15L_648_s | 6.71E-04 | 6.92 |
| B5L_33_a | 6.74E-04 | 3.07 |
| I8R | 6.79E-04 | 3.23 |
| C11L_70_s | 7.17E-04 | 4.62 |
| B19R | 7.18E-04 | 4.69 |
| A7L | 7.31E-04 | 5.48 |
| I1L_156_s | 7.63E-04 | 4.7 |
| C9L_552_s | 7.72E-04 | 4.81 |
| B14R | 7.83E-04 | 4.02 |
| A28L | 7.89E-04 | 4.38 |
| J9R_368_a | 7.91E-04 | 4.98 |
| C18L_11_s | 8.00E-04 | 4.85 |
| A19L_33_a | 8.09E-04 | 2.34 |
| L2R | 8.10E-04 | 6.08 |
| A9L_98_a | 8.16E-04 | 5.1 |
| G3R | 8.31E-04 | 4.65 |
| C10L_61_s | 8.40E-04 | 3.51 |
| A50R | 8.79E-04 | 4.71 |
| A36R | 8.85E-04 | 4.57 |
| B7R | 9.04E-04 | 4.92 |
| J1R | 9.12E-04 | 3.29 |
| G2L | 9.66E-04 | 3.99 |
| R1R | 9.67E-04 | 3.82 |
| A49L_40_s | 9.71E-04 | 5.75 |
| C13L | 9.79E-04 | 3.82 |
| A34L | 9.97E-04 | 3.86 |
| D11L_196_s | 1.01E-03 | 3.71 |
| E4L_404_a | 1.04E-03 | 2.86 |
| G3R_1277_a | 1.04E-03 | 3.35 |
| I5L | 1.05E-03 | 3.27 |
| G1L | 1.05E-03 | 3.51 |
| D12L | 1.10E-03 | 5.61 |
| I1L_156_a | 1.11E-03 | 2.09 |
| L4R_202_a | 1.17E-03 | 6.56 |
| F2L | 1.17E-03 | 5.26 |
| D8L_266_s | 1.21E-03 | 6.07 |
| E1R | 1.26E-03 | 5.29 |
| H6R_295_s | 1.26E-03 | 3.33 |
| F6R_1279_a | 1.27E-03 | 2.34 |
| F2L_98_s | 1.27E-03 | 2.31 |
| C10L_4_s | 1.27E-03 | 5.51 |
| C21R_167_a | 1.30E-03 | 4.33 |
| A49L_40_a | 1.30E-03 | 2.59 |
| B4L_142_a | 1.34E-03 | 3.47 |
| A18R_1143_a | 1.34E-03 | 2.91 |
| G10R | 1.37E-03 | 3.26 |
| A38R_10_a | 1.41E-03 | 5.11 |
| A19R | 1.41E-03 | 4.98 |
| J3L | 1.43E-03 | 3.03 |
| Q2L | 1.44E-03 | 6.09 |
| L6R | 1.44E-03 | 4.95 |
| B2R | 1.45E-03 | 4.68 |
| D9L_280_s | 1.46E-03 | 5.76 |
| B15L | 1.47E-03 | 2.96 |
| E11L | 1.50E-03 | 3.15 |
| B14R_217_a | 1.55E-03 | 6 |
| B18R | 1.55E-03 | 3.28 |
| K7L_1004_a | 1.55E-03 | 2.07 |
| B21R_1508_a | 1.59E-03 | 4.77 |
| E1L_436_s | 1.62E-03 | 2.41 |
| K8R_1407_a | 1.67E-03 | 3.02 |
| C17L_1049_s | 1.69E-03 | 6.19 |
| B8R_17_a | 1.70E-03 | 5.8 |
| F10L | 1.73E-03 | 4.55 |
| G5R | 1.80E-03 | 4.53 |
| A1L | 1.80E-03 | 3.86 |
| A32L | 1.81E-03 | 3.39 |
| B1R | 1.83E-03 | 3.14 |
| C6R | 1.86E-03 | 3.93 |
| A29L | 1.88E-03 | 5.07 |
| A23R_1009_a | 1.90E-03 | 5.18 |
| H1L | 1.91E-03 | 3.59 |
| F1L | 1.97E-03 | 5.01 |
| H4L | 2.02E-03 | 2.93 |
| A20L | 2.05E-03 | 4.77 |
| B12R_277_a | 2.11E-03 | 4.29 |
| A3L_1405_s | 2.16E-03 | 4.93 |
| C13L_302_s | 2.23E-03 | 3.46 |
| C23R | 2.24E-03 | 4.27 |
| A37R_33_a | 2.27E-03 | 4.18 |
| E2L_1970_a | 2.33E-03 | 3.97 |
| F10R_106_s | 2.37E-03 | 3.52 |
| A50L_12_s | 2.39E-03 | 2.08 |
| G9R | 2.47E-03 | 4.11 |
| A12L_145_s | 2.48E-03 | 5.45 |
| A4L_567_s | 2.48E-03 | 4.71 |
| B2L_115_a | 2.50E-03 | 3.41 |
| F9R | 2.53E-03 | 3.91 |
| M4R | 2.54E-03 | 4.1 |
| F4R_195_a | 2.57E-03 | 4.61 |
| A36R_196_a | 2.57E-03 | 4.92 |
| A42R | 2.62E-03 | 3.67 |
| B13R_724_a | 2.63E-03 | 5.57 |
| I1L | 2.70E-03 | 5.57 |
| D1L | 2.72E-03 | 5.76 |
| A18L | 2.75E-03 | 2.73 |
| L4R | 2.78E-03 | 6.02 |
| H7R | 2.84E-03 | 5.21 |
| E8L | 2.91E-03 | 4.35 |
| E6R | 2.93E-03 | 4.51 |
| H6R | 2.95E-03 | 4.47 |
| B8R | 3.00E-03 | 3.92 |
| A6L_1049_a | 3.02E-03 | 3.2 |
| L1R | 3.06E-03 | 4.8 |
| A13L | 3.09E-03 | 5.52 |
| A41L_722_a | 3.15E-03 | 4.51 |
| I6L | 3.20E-03 | 3.34 |
| I2R_153_s | 3.21E-03 | 4.72 |
| D4R_184_a | 3.34E-03 | 4.11 |
| C15L | 3.40E-03 | 3.24 |
| D10L_70_a | 3.42E-03 | 2.85 |
| C11L | 3.49E-03 | 4.67 |
| A8L | 3.55E-03 | 3.57 |
| C3L | 3.62E-03 | 3.22 |
| B26R_5110_a | 3.63E-03 | 4.52 |
| E8R_1_a | 3.67E-03 | 5.51 |
| M5R_317_a | 3.75E-03 | 4.63 |
| B7R_657_a | 3.92E-03 | 4.34 |
| G4L | 4.01E-03 | 2.54 |
| A3L | 4.22E-03 | 4.4 |
| C20L_303_s | 4.24E-03 | 5.19 |
| E9L_2465_s | 4.25E-03 | 4.99 |
| D14L | 4.36E-03 | 4.16 |
| A2L | 4.37E-03 | 4.12 |
| B6R | 4.51E-03 | 3.93 |
| C21R_167_s | 4.59E-03 | 2.1 |
| A40_5R_80_a | 4.66E-03 | 4.08 |
| E7R_92_a | 4.90E-03 | 5.02 |
| A40L | 5.02E-03 | 4.23 |
| A11L | 5.05E-03 | 4.35 |
| A46R | 5.06E-03 | 3.78 |
| A30L | 5.06E-03 | 3.03 |
| F6R | 5.15E-03 | 4.96 |
| L5L_262_a | 5.18E-03 | 4.68 |
| H3L | 5.25E-03 | 3.47 |
| N2R | 5.30E-03 | 2.64 |
| L3R_162_a | 5.36E-03 | 5.2 |
| I3L_656_s | 5.48E-03 | 4.58 |
| E3R | 5.68E-03 | 3.26 |
| F9R_94_s | 5.70E-03 | 4.51 |
| M1R | 5.82E-03 | 4.07 |
| M5R | 5.84E-03 | 4.41 |
| E13L | 5.97E-03 | 4.14 |
| I7L | 6.06E-03 | 3.09 |
| B21R_1508_s | 6.25E-03 | 2.15 |
| F5R | 6.45E-03 | 3.5 |
| E1L_436_a | 6.52E-03 | 4.47 |
| H2R | 6.68E-03 | 4.23 |
| H2L_173_a | 6.72E-03 | 2.42 |
| K1R | 6.74E-03 | 2.7 |
| A31L_180_s | 6.74E-03 | 4.85 |
| A14L | 6.77E-03 | 4.46 |
| A4L_105_s | 6.90E-03 | 4.8 |
| C4L | 6.97E-03 | 3.21 |
| H9R_682_a | 7.21E-03 | 5.15 |
| B16L_126_a | 7.59E-03 | 3.35 |
| A16L | 7.72E-03 | 3.83 |
| A8R_0_a | 7.91E-03 | 2.86 |
| E10R | 8.03E-03 | 3.32 |
| B5R | 8.06E-03 | 3.27 |
| A17L | 8.32E-03 | 4.53 |
| J2R_52_a | 8.49E-03 | 4.21 |
| H7L_771_s | 8.55E-03 | 3.14 |
| E11L_18_s | 8.74E-03 | 3.42 |
| A1L_197_s | 8.92E-03 | 4.33 |
| C19L | 9.17E-03 | 4.45 |
| C4R_142_s | 9.34E-03 | 2.98 |
| D14L_464_a | 9.42E-03 | 2.12 |
| A26L | 9.45E-03 | 2.63 |
| I4L_1860_s | 9.47E-03 | 3.25 |
| I7R_207_a | 9.92E-03 | 4.9 |
| A19L_33_s | 1.00E-02 | 4.24 |
| Differential Viral proteins - Mock vs VACV (Primary Human Macrophage)-140 | | |
| SPOT_ID | P.Value | logFC |
| H5R | 1.53E-18 | 7.54919 |
| F4L | 2.99E-17 | 6.13471 |
| C1L | 2.25E-16 | 6.71867 |
| B9R | 2.92E-16 | 5.65289 |
| L5L | 4.05E-16 | 6.92591 |
| I3L | 5.27E-16 | 6.04752 |
| A51R | 5.78E-16 | 6.05127 |
| D3R | 6.88E-16 | 5.97529 |
| O2L | 1.24E-15 | 5.8008 |
| C8L | 4.13E-15 | 6.35243 |
| A37R | 4.37E-15 | 5.91548 |
| M2R | 5.03E-15 | 5.11125 |
| M3L | 6.36E-15 | 4.87806 |
| A10L | 8.00E-15 | 5.70085 |
| A49R | 9.46E-15 | 6.70226 |
| C21L | 9.46E-15 | 5.38337 |
| A33R | 1.03E-14 | 5.28398 |
| A39R | 1.45E-14 | 5.30338 |
| C5L | 1.85E-14 | 5.94463 |
| P1L | 2.31E-14 | 5.32292 |
| B4R | 2.32E-14 | 4.55746 |
| B13R | 4.11E-14 | 4.62404 |
| D19L | 5.85E-14 | 5.34954 |
| B3R | 6.12E-14 | 5.5937 |
| F3L | 9.99E-14 | 5.11584 |
| E4R | 1.20E-13 | 5.16157 |
| A45L | 1.21E-13 | 5.13623 |
| A9R | 1.49E-13 | 4.63432 |
| I2L | 2.13E-13 | 4.58665 |
| D10L | 3.17E-13 | 4.71387 |
| D12L | 6.35E-13 | 4.52917 |
| C18L | 6.97E-13 | 4.84252 |
| G6R | 9.22E-13 | 4.18726 |
| D8L | 1.13E-12 | 3.92351 |
| A44R | 1.18E-12 | 4.22285 |
| A49L_40_s | 1.30E-12 | 4.84614 |
| B12R | 2.01E-12 | 4.99431 |
| C20L | 2.92E-12 | 4.30482 |
| L2R | 3.06E-12 | 4.14064 |
| A35R | 3.47E-12 | 4.07627 |
| A47R | 3.61E-12 | 4.45177 |
| D5R | 5.31E-12 | 4.53319 |
| E12L | 1.07E-11 | 4.56197 |
| C7L | 1.36E-11 | 3.65711 |
| P2L | 1.47E-11 | 3.77898 |
| I4L | 2.33E-11 | 4.13509 |
| E1R | 2.38E-11 | 3.98065 |
| J3R | 3.44E-11 | 4.02856 |
| D13L | 3.57E-11 | 4.33414 |
| A7L | 4.69E-11 | 4.37445 |
| L6R | 5.89E-11 | 3.79427 |
| F7R | 6.32E-11 | 4.23613 |
| D4L | 7.75E-11 | 3.77967 |
| B19R | 1.00E-10 | 4.11159 |
| L3R | 2.02E-10 | 3.67956 |
| D9L | 2.40E-10 | 3.3217 |
| A6R | 2.59E-10 | 3.86486 |
| A31L | 3.45E-10 | 3.62442 |
| A24R | 8.61E-10 | 3.59024 |
| E9R | 9.34E-10 | 3.50992 |
| F8L | 1.06E-09 | 3.75045 |
| C14L | 1.35E-09 | 3.16539 |
| G2L | 1.51E-09 | 3.19628 |
| G5R | 1.53E-09 | 2.62593 |
| E5R | 1.76E-09 | 3.3088 |
| C11L | 2.04E-09 | 3.28027 |
| A22R | 2.07E-09 | 3.26887 |
| E2L | 3.36E-09 | 2.97416 |
| E8L | 3.53E-09 | 3.4096 |
| A41L | 3.55E-09 | 3.15698 |
| J1L | 3.58E-09 | 2.91851 |
| C6R | 7.76E-09 | 2.81137 |
| H2R | 1.11E-08 | 3.53051 |
| E8R_1_a | 1.24E-08 | 4.37907 |
| A40L | 1.47E-08 | 3.4418 |
| D18L | 1.82E-08 | 3.61569 |
| A5L | 2.20E-08 | 3.22016 |
| B20R | 2.46E-08 | 3.81953 |
| I1L | 4.27E-08 | 3.80203 |
| A4L | 5.12E-08 | 4.17465 |
| B7R_657_a | 6.27E-08 | 3.692 |
| C2L | 7.61E-08 | 3.1911 |
| A50R | 9.29E-08 | 2.913 |
| A34L | 1.06E-07 | 2.77248 |
| D17L | 1.06E-07 | 2.98272 |
| A20L | 1.07E-07 | 2.95845 |
| A4L_567_s | 1.12E-07 | 3.36911 |
| H7R | 1.21E-07 | 3.42354 |
| A11L | 1.46E-07 | 3.20645 |
| L6R_3484_a | 1.63E-07 | 3.03458 |
| I6L | 1.79E-07 | 2.4351 |
| M1R | 2.09E-07 | 3.53682 |
| B14R | 3.66E-07 | 2.56824 |
| B6R | 4.45E-07 | 2.86392 |
| A29L | 7.31E-07 | 3.47211 |
| A16L | 8.00E-07 | 3.0688 |
| C4L | 9.78E-07 | 2.53619 |
| Q2L | 1.02E-06 | 4.39564 |
| A1L | 1.14E-06 | 2.86345 |
| H6R | 1.15E-06 | 3.39786 |
| F1L | 1.19E-06 | 3.72546 |
| A19R | 1.19E-06 | 3.34398 |
| C19L | 1.21E-06 | 3.47188 |
| A15L | 1.76E-06 | 4.43367 |
| G9R | 1.78E-06 | 2.92448 |
| D6L | 1.80E-06 | 2.48855 |
| A17L | 1.83E-06 | 3.16147 |
| E13L | 2.49E-06 | 2.26272 |
| B8R | 3.09E-06 | 2.68122 |
| A42R | 3.33E-06 | 2.52017 |
| E6R | 3.70E-06 | 2.93581 |
| A46R | 4.02E-06 | 2.6405 |
| B1R | 4.33E-06 | 2.15566 |
| A28L | 4.77E-06 | 2.74229 |
| B2R | 5.01E-06 | 2.18568 |
| E11L | 5.41E-06 | 2.16766 |
| L1R | 5.54E-06 | 3.14877 |
| A13L | 5.90E-06 | 3.50202 |
| F6R | 6.39E-06 | 2.76473 |
| A12R | 7.75E-06 | 2.72658 |
| A8R_0_a | 8.33E-06 | 2.27784 |
| A21L | 1.19E-05 | 2.34963 |
| D15L | 1.33E-05 | 2.07758 |
| D16L | 1.94E-05 | 2.30012 |
| A27L | 2.13E-05 | 2.30545 |
| A20L_29_a | 2.49E-05 | 2.07813 |
| E3R | 5.19E-05 | 2.38958 |
| A17L_370_s | 5.39E-05 | 3.36532 |
| A26L | 5.86E-05 | 2.12344 |
| E10R | 6.47E-05 | 2.10102 |
| A32L | 6.90E-05 | 2.10726 |
| M4R | 7.67E-05 | 2.54817 |
| G10R | 9.75E-05 | 2.1322 |
| A28L_1230_s | 1.19E-04 | 2.35083 |
| A14L | 2.13E-04 | 2.33161 |
| F9R_94_s | 3.81E-04 | 2.24974 |
| E7R_92_a | 4.83E-04 | 2.00514 |
| A42R_102_a | 1.07E-03 | 2.22503 |
| N1R | 2.42E-03 | 2.0078 |
| Differential viral proteins -Mock vs VACV Hela - 111 | | |
| SPOT_ID | P.Value | logFC |
| D3R | 3.59E-06 | 6.82 |
| A33R | 4.72E-06 | 6.61 |
| A44R | 6.87E-06 | 6.1 |
| I3L | 1.04E-05 | 6.93 |
| H5R | 1.14E-05 | 7.6 |
| C1L | 1.28E-05 | 5.89 |
| A37R | 2.06E-05 | 7.05 |
| B16R | 2.17E-05 | 6.76 |
| A45L | 2.48E-05 | 5.74 |
| F4L | 2.49E-05 | 7.04 |
| C5L | 2.55E-05 | 4.81 |
| C21L | 3.47E-05 | 6.34 |
| A47R | 3.65E-05 | 5.89 |
| B3R | 3.96E-05 | 5.76 |
| A7L | 5.75E-05 | 5.29 |
| B4R | 6.32E-05 | 5.28 |
| A9R | 6.47E-05 | 5.36 |
| L2R | 6.77E-05 | 5.65 |
| C10L | 6.83E-05 | 6.13 |
| B9R | 6.87E-05 | 6.03 |
| B7R | 7.25E-05 | 4.61 |
| A51R | 8.23E-05 | 6.2 |
| A39R | 1.03E-04 | 5.15 |
| B11R | 1.05E-04 | 5.47 |
| D19L | 1.05E-04 | 5.61 |
| O2L | 1.12E-04 | 5.84 |
| C17L | 1.12E-04 | 6.63 |
| E1R | 1.15E-04 | 5.64 |
| P1L | 1.22E-04 | 5.91 |
| J1L | 1.26E-04 | 5.24 |
| J3R | 1.39E-04 | 5.66 |
| I2L | 1.44E-04 | 5.11 |
| E5R | 1.49E-04 | 5.62 |
| D10L | 1.70E-04 | 5 |
| D8L | 1.77E-04 | 6.33 |
| A25R | 1.82E-04 | 5.81 |
| E12L | 1.87E-04 | 5.7 |
| D5R | 2.09E-04 | 5.25 |
| A10L | 2.10E-04 | 5.85 |
| A49R | 2.11E-04 | 5.94 |
| C22L | 2.20E-04 | 4.64 |
| Q1L | 2.22E-04 | 5.31 |
| M3L | 2.39E-04 | 4.85 |
| C18L | 2.40E-04 | 6.05 |
| M2R | 2.48E-04 | 4.92 |
| F3L | 2.53E-04 | 4.46 |
| G6R | 2.60E-04 | 5.07 |
| B2R | 2.65E-04 | 4.13 |
| A40L | 2.68E-04 | 4.99 |
| L4R | 3.39E-04 | 5.63 |
| B13R | 3.41E-04 | 5.26 |
| L6R | 3.95E-04 | 5.17 |
| E7R | 4.05E-04 | 5.84 |
| G7R | 4.15E-04 | 3.91 |
| C13L | 4.58E-04 | 3.49 |
| A41L | 4.73E-04 | 4.9 |
| G2L | 5.48E-04 | 3.55 |
| C20L | 6.15E-04 | 4.04 |
| C6R | 8.13E-04 | 4.74 |
| A38R | 8.15E-04 | 4.32 |
| H7R | 8.67E-04 | 4.32 |
| C7L | 9.01E-04 | 4.6 |
| I4L | 9.14E-04 | 4.63 |
| C8L | 9.57E-04 | 5.39 |
| A31L | 1.07E-03 | 5.12 |
| C2L | 1.22E-03 | 4.86 |
| F10L | 1.27E-03 | 5 |
| A35R | 1.35E-03 | 5.1 |
| E8L | 1.35E-03 | 3.86 |
| A3L | 1.65E-03 | 4.82 |
| P2L | 1.85E-03 | 4.05 |
| J3L | 1.92E-03 | 3.05 |
| J1R | 1.95E-03 | 2.72 |
| G9R | 1.98E-03 | 4.4 |
| F6R | 2.06E-03 | 4.24 |
| A16L | 2.33E-03 | 4.47 |
| A2L | 2.44E-03 | 4.94 |
| D18L | 2.56E-03 | 4.6 |
| F1L | 2.73E-03 | 5 |
| B12R | 2.86E-03 | 3.92 |
| B5R | 2.91E-03 | 3.11 |
| C11L | 3.02E-03 | 4.42 |
| D12L | 3.02E-03 | 5.38 |
| A11L | 3.10E-03 | 3.67 |
| C12L | 3.21E-03 | 2.99 |
| L5L | 3.37E-03 | 4.87 |
| C15L | 3.43E-03 | 3.49 |
| A19R | 3.47E-03 | 3.78 |
| C19L | 3.50E-03 | 4.79 |
| H1L | 3.51E-03 | 3.73 |
| A34L | 3.64E-03 | 3.58 |
| A23R | 3.78E-03 | 3.28 |
| A36R | 4.39E-03 | 3.41 |
| Q2L | 4.41E-03 | 5.16 |
| D13L | 4.83E-03 | 4.52 |
| A20L | 4.87E-03 | 4.19 |
| D7L | 5.23E-03 | 3.05 |
| I1L | 5.51E-03 | 6.23 |
| I7L | 5.55E-03 | 3.15 |
| A48R | 5.59E-03 | 2.38 |
| B6R | 5.75E-03 | 3.45 |
| M5R | 5.98E-03 | 3.2 |
| A32L | 6.50E-03 | 2.92 |
| I5L | 6.57E-03 | 2.22 |
| A6R | 6.58E-03 | 4.77 |
| A21L | 7.10E-03 | 3.51 |
| A50R | 7.67E-03 | 3.51 |
| A24R | 7.83E-03 | 3.66 |
| A1L | 8.27E-03 | 4.11 |
| A4L | 8.53E-03 | 3.86 |
| B19R | 8.58E-03 | 2.95 |
| Differential Viral proteins - Mock vs VACV(Primary Human Fibroblast)-330 | | |
| SPOT_ID | P.Value | logFC |
| A48R | 7.75E-09 | 2.12 |
| H2L_173_a | 1.64E-07 | 2.41 |
| E3L_313_s | 3.41E-07 | 6.73 |
| E1R | 6.16E-07 | 4.4 |
| E4L_404_s | 6.37E-07 | 6.32 |
| M2R_124_a | 2.02E-06 | 5.49 |
| D11L_196_s | 2.49E-06 | 5.51 |
| F7R_251_a | 3.20E-06 | 3.8 |
| A9L_98_a | 3.22E-06 | 4 |
| C6L_317_s | 3.94E-06 | 5.2 |
| A52R_541_a | 5.09E-06 | 5.49 |
| B20R_776_a | 5.10E-06 | 5.5 |
| N4R | 6.61E-06 | 2.53 |
| H3L | 6.66E-06 | 2.78 |
| C5L_62_s | 1.02E-05 | 5.58 |
| EMPTY | 1.06E-05 | 3.53 |
| A37R | 1.15E-05 | 5.55 |
| J2R_52_a | 1.29E-05 | 4.48 |
| B3L_88_a | 1.36E-05 | 4.99 |
| A38R_10_a | 1.58E-05 | 4.45 |
| C7L | 1.67E-05 | 3.55 |
| J3R_6_a | 2.13E-05 | 4.03 |
| B9R | 2.23E-05 | 4.35 |
| B2L_115_a | 2.29E-05 | 5.09 |
| C4R_142_a | 2.77E-05 | 4.18 |
| D10L_70_s | 3.06E-05 | 5.34 |
| C14L_1022_s | 3.38E-05 | 4.94 |
| B13R_724_a | 3.59E-05 | 4.11 |
| A39R | 3.83E-05 | 4.55 |
| H5_5R_11_a | 3.92E-05 | 4.53 |
| D11L | 4.02E-05 | 3.21 |
| C15L_648_s | 4.17E-05 | 5.64 |
| D3R | 4.19E-05 | 4.96 |
| J6R_73_a | 4.21E-05 | 4.96 |
| C17L | 4.37E-05 | 5.31 |
| A36R_196_a | 4.48E-05 | 5.11 |
| I3L | 4.51E-05 | 5.18 |
| E9L_2465_s | 5.09E-05 | 4.87 |
| C21L | 5.20E-05 | 4.91 |
| A44R | 6.55E-05 | 3.82 |
| B4R | 6.73E-05 | 3.09 |
| O1L | 6.76E-05 | 4.24 |
| B13R | 6.89E-05 | 4.07 |
| I2R_153_s | 7.11E-05 | 4.89 |
| C8L_663_s | 7.26E-05 | 5.68 |
| B12R_277_a | 7.45E-05 | 4.54 |
| A33R | 7.49E-05 | 4.8 |
| A47R | 7.89E-05 | 4.14 |
| I2L | 8.35E-05 | 3.7 |
| B14R_217_a | 8.49E-05 | 5.08 |
| A34R_223_a | 8.91E-05 | 5.14 |
| D5L_20_s | 9.25E-05 | 4.45 |
| C3L | 9.54E-05 | 3.33 |
| B8R_17_a | 9.54E-05 | 3.82 |
| C1L_88_s | 9.62E-05 | 4.7 |
| J3R | 9.63E-05 | 3.85 |
| A21R_1046_s | 9.90E-05 | 2.12 |
| C22L | 9.93E-05 | 4.51 |
| LC:17996 | 1.01E-04 | 2.43 |
| F9R_94_a | 1.03E-04 | 2.59 |
| C18L_11_a | 1.09E-04 | 2.47 |
| C10L_61_s | 1.11E-04 | 4.82 |
| A40_5R_80_a | 1.14E-04 | 4.81 |
| B16R | 1.20E-04 | 4.67 |
| D8L | 1.25E-04 | 3.85 |
| J5R_472_a | 1.25E-04 | 4.6 |
| LCP:63 | 1.30E-04 | 2.08 |
| B9R_274_a | 1.35E-04 | 5.14 |
| A7L | 1.38E-04 | 3.47 |
| A41L_722_a | 1.49E-04 | 3.2 |
| H5R | 1.49E-04 | 5.44 |
| F4L | 1.50E-04 | 4.49 |
| A49L_40_s | 1.53E-04 | 3.2 |
| C10L | 1.73E-04 | 4.46 |
| C10L_4_s | 1.75E-04 | 3.63 |
| B11R | 1.86E-04 | 4.61 |
| E5R_723_a | 1.87E-04 | 4.96 |
| A35L_163_a | 1.90E-04 | 3.93 |
| A51R | 1.90E-04 | 4.48 |
| A9R | 1.94E-04 | 4.14 |
| J1L | 1.95E-04 | 3.27 |
| D14L_464_s | 1.96E-04 | 5.32 |
| D12L | 1.97E-04 | 3.55 |
| D19L | 1.98E-04 | 4.43 |
| G4R_819_a | 1.99E-04 | 2.48 |
| B7R | 2.04E-04 | 2.84 |
| C20L | 2.07E-04 | 3.79 |
| A40R_52_a | 2.11E-04 | 2.61 |
| D4L | 2.16E-04 | 3.03 |
| A31L | 2.21E-04 | 3.66 |
| E7R | 2.22E-04 | 4.46 |
| Q1L | 2.35E-04 | 4.52 |
| C20L_303_s | 2.36E-04 | 3.76 |
| L5L | 2.43E-04 | 4.28 |
| C8L | 2.54E-04 | 4.73 |
| G8L | 2.57E-04 | 2.25 |
| M2R | 2.75E-04 | 4.28 |
| D5R | 2.76E-04 | 4.2 |
| A5R_161_a | 2.78E-04 | 2.93 |
| G6R | 2.81E-04 | 3.92 |

**Supplementary Table S4:** Overlap of Viral proteins from different cell-type (Monkeypox and Vaccinia)

**Monkeypox viral proteins**

| B9R | A41L | E10R | A51R | C4L | A19R |
| --- | --- | --- | --- | --- | --- |
| A10L | B16R | A25R | D3R | D10L | D1L |
| A35R | L1R | D4L | N4R | F3L | H5R |
| D7L | J2R | A45L | A7L | E8L | C17L |
| A1L | A20L | A40L | B6R | A42R | C21L |
| F10L | B10R | B21R | A3L | B3R | A34L |
| M5R | C13L | C2L | D15L | M1R | C1L |
| A31L | F4L | G6R | P1L | C10L | M3L |
| D19L | A38R | C20L | A9R | L4R | I4L |
| B19R | A39R | A49R | B11R | D8L | C11L |
| C12L | G7R | A21L | E7R | B12R | J3R |
| C19L | B17R | A33R | H7R | E1R | B2R |
| F7R | O2L | I1L | B7R | C7L | E2L |
| J3L | D12L | C8L | A47R | A22R |  |
| F6R | A13L | A46R | E5R | L6R |  |
| F1L | C18L | B13R | A50R | A44R |  |
| C5L | E12L | P2L | L5L | C6R |  |
| E6R | B20R | B4R | A28L | Q2L |  |
| A48R | J2L | D13L | I2L | J1L |  |
| Q1L | M2R | D18L | I3L | N3R |  |
| D6L | L2R | D2L | F2L | C22L |  |

**Vaccinia viral proteins**

| A10L | M2R | A7L | B12R | J3R |
| --- | --- | --- | --- | --- |
| B9R | L2R | B6R | E1R | B2R |
| A35R | A37R | P1L | C7L |  |
| A1L | A45L | A9R | L6R |  |
| A11L | A40L | B11R | A44R |  |
| A31L | C2L | E7R | D5R |  |
| D19L | G6R | H7R | C6R |  |
| B19R | C20L | A47R | A32L |  |
| F1L | A49R | E5R | Q2L |  |
| C5L | A21L | A50R | J1L |  |
| A41L | A33R | L5L | A19R |  |
| B16R | G2L | I2L | H5R |  |
| A20L | I1L | I3L | C17L |  |
| F4L | C8L | D10L | A6R |  |
| G9R | B13R | F3L | C21L |  |
| A39R | P2L | E8L | A24R |  |
| O2L | B4R | B3R | A34L |  |
| A4L | D13L | C10L | C1L |  |
| D12L | D18L | L4R | M3L |  |
| C18L | A51R | D8L | I4L |  |
| E12L | D3R | A16L | C11L |  |

**Supplementary Table S5:** Detailed Description of Viral Protein function -Monkeypox

| **Viral_Protein** | **Category** | **GO_Biological_Process** | **GO_Molecular_Function** | **GO_Cellular_Component** | **Function_Description** |
| --- | --- | --- | --- | --- | --- |
| **B9R | Viromimicry | interferon-gamma-mediated signaling pathway | cytokine receptor activity |  |  |
| *A10L | Viral morphogenesis |  |  | cytoplasm,host cell cytoplasm,integral component of membrane,virion membrane | Envelope protein. Required for an early step in virion morphogenesis |
| *A35R | Viral morphogenesis |  |  | integral component of membrane | cell-cell spread |
| **D7L | Virokine |  |  |  | IL-18 |
| *A1L | Viral replication | DNA-templated viral transcription | identical protein binding,zinc ion binding(late gene transcription factor) |  | Acts with RNA polymerase to initiate transcription from late gene promoters |
| *F10L | Viral morphogenesis |  |  | virion component |  |
| *M5R | Viral entry | fusion of virus membrane with host plasma membrane |  | integral component of membrane,virion membrane |  |
| *A31L | Viral replication | viral transcription | DNA-directed 5'-3' RNA polymerase activity,DNA binding | DNA-directed RNA polymerase complex,virion component | Part of the DNA-dependent RNA polymerase which catalyzes the transcription of viral DNA into RNA using the four ribonucleoside triphosphates as substrates. Responsible for the transcription of early, intermediate and late genes |
| **D19L | Virotransducers |  |  |  | BCL2-like protein |
| **B19R | Virotransducers |  |  | host cell surface | May bind interleukin-1 and/or interleukin-6 and prevent these cytokines reaching their natural receptors. In consequence the inflammatory response would be diminished and virus replication enhanced. |
| **C12L | Viromimicry |  |  |  | SERP-1 |
| **C19L | Virion maturation |  | catalytic activity |  | Ankyrin like protein |
| *F7R | Viral morphogenesis |  |  | integral component of membrane |  |
| **J3L | Virotransducers |  |  |  |  |
| *F6R | Viral entry |  |  |  |  |
| **F1L | Virotransducers | negative regulation of interleukin-1 production,suppression by virus of host apoptotic process |  | host cell mitochondrion | Plays a role in evading host innate immune response by inhibiting host inflammasome activation. Interacts with and inhibits NLR-mediated interleukin-1 beta/IL1B production in infected cells. At the host mitochondria, interacts with the BH3 domain of host BAK and prevents BAK from binding active BAX. In turn, host apoptosis is inhibited. |
| **C5L | Viromimicry |  |  |  |  |
| *E6R | Viral morphogenesis |  |  | virion component | Late protein which may play a role in the virion morphogenesis and have therefore an indirect role on viral transcription ability. |
| *A48R | Viral replication | dTDP biosynthetic process,dTTP biosynthetic process,phosphorylation | ATP binding,thymidylate kinase activity |  | Poxvirus TMP kinase is able to phosphorylate dTMP, dUMP and also dGMP from any purine and pyrimidine nucleoside triphosphate. The large substrate specificity is explained by the presence of a canal connecting the edge of the dimer interface to the TMP base binding pocket, canal not found in the human homolog (By similarity) |
| Q1L | unknown |  |  |  | membrane protein |
| **A41L | Virostealth | mitigation of host antiviral defense response,suppression by virus of host chemokine activity |  | extracellular region | May interact with several cellular chemokines to interfere with chemokine-glycosaminoglycan (GAG) interactions at the cell surface to alter chemotaxis of nearby responsive cells. |
| **B16R | Virotransducers |  |  |  |  |
| *L1R | Viral entry | viral entry into host cell, virion attachment to host cell |  | integral component of membrane,viral envelope,virion membrane | Envelope protein which probably plays a role in virus entry into the host cell. Is probably involved in the virus attachment to the host cell surface and associates with the entry/fusion complex (EFC). Needed for fusion and penetration of the virus core into host cell |
| *J2R | Viral replication | DNA biosynthetic process,phosphorylation | ATP binding,metal ion binding,thymidine kinase activity |  | Phosphorylates thymidine and thymidine analogs, such as azidothymidine (AZT). Part of the salvage pathway for pyrimidine deoxyribonucleotide synthesis. |
| *A20L | unknown |  |  |  |  |
| *B10R | Virotransducers |  |  |  | POXVIRUS VIRULENCE |
| *C13L | unknown |  |  |  |  |
| *F4L | Viral replication | DNA-templated transcription | DNA binding,DNA-directed 5'-3' RNA polymerase activity,zinc ion binding | DNA-directed RNA polymerase complex |  |
| *A38R | Viral morphogenesis |  |  | integral component of membrane |  |
| **A39R | virokine |  | semaphorin receptor binding | extracellular region | Acts as a semaphorin-like protein and binds to host plexin C1 receptor. May alter the movement of host plexin C1-expressing cells including dendritic cells, monocytes, or granulocytes in the proximity of infected cells. May also regulate host cell cytoskeleton of neighboring cells to improve viral infection (By similarity). |
| **G7R | Viral transcription |  |  |  |  |
| **B17R | Virotransducers |  |  |  |  |
| *O2L | Viral replication |  | glutathione oxidoreductase activity,protein-disulfide reductase (glutathione) activity | virion component | Displays thioltransferase and dehydroascorbate reductase activities. |
| **D12L | Viral transcription | DNA-templated transcription termination | mRNA (guanine-N7-)-methyltransferase activity | virion component | Regulatory subunit of the mRNA cap enzyme which stabilizes the catalytic subunit and enhances its methyltransferase activity through an allosteric mechanism. Heterodimeric mRNA capping enzyme catalyzes the linkage of a N7-methyl-guanosine moiety to the first transcribed nucleotide (cap 0 structure), whereas the polymerase associated VP39 is responsible for a second methylation at the 2'-O position of the ribose (cap 1 structure). The heterodimeric enzyme is also involved in early viral gene transcription termination and intermediate viral gene transcription initiation. Early gene transcription termination requires the termination factor VTF, the DNA-dependent ATPase NPH-I and the Rap94 subunit of the viral RNA polymerase, as well as the presence of a specific termination motif. Binds, together with RAP94, to the termination motif 5'-UUUUUNU-3' in the nascent early mRNA. |
| *A13L | Viral morphogenesis |  |  | integral component of membrane,virion membrane | Essential for the encapsidation of DNA into immature virions (IV) and the subsequent maturation of IV into mature virions (MV). |
| **C18L | Virotransducers |  |  |  | Kelch-like protein |
| *E12L | Viral transcription | DNA-templated transcription termination | mRNA (guanine-N7-)-methyltransferase activity |  | Regulatory subunit of the mRNA cap enzyme which stabilizes the catalytic subunit and enhances its methyltransferase activity through an allosteric mechanism. Heterodimeric mRNA capping enzyme catalyzes the linkage of a N7-methyl-guanosine moiety to the first transcribed nucleotide (cap 0 structure), whereas the polymerase associated VP39 is responsible for a second methylation at the 2'-O position of the ribose (cap 1 structure).  The heterodimeric enzyme is also involved in early viral gene transcription termination and intermediate viral gene transcription initiation. Early gene transcription termination requires the termination factor VTF, the DNA-dependent ATPase NPH-I and the Rap94 subunit of the viral RNA polymerase, as well as the presence of a specific termination motif. Binds, together with RAP94, to the termination motif 5'-UUUUUNU-3' in the nascent early mRNA. |
| **B20R | Virotransducers |  |  |  |  |
| **J2L | Virotransducers | mitigation of host antiviral defense response | tumor necrosis factor receptor activity | extracellular region |  |
| *M2R | Viral morphogenesis |  |  | integral component of membrane,virion component | Early protein involved in early virion morphogenesis. Participates in the formation and elongation of crescent-shaped membrane precursors of immature virions in cytoplasmic factories |
| *L2R | Viral morphogenesis |  |  | integral component of membrane,virion component,host cell cytoplasm | Early protein involved in early virion morphogenesis. Participates in the formation and elongation of crescent-shaped membrane precursors of immature virions in cytoplasmic factories (By similarity). |
| **D6L | Virokine |  |  |  |  |
| *E10R | Viral morphogenesis |  | thiol oxidase activity | integral component of membrane,virion component,viral envelope | FAD-dependent sulfhydryl oxidase that catalyzes disulfide bond formation. The complete pathway for formation of disulfide bonds in intracellular virion membrane proteins sequentially involves oxidation of E10, A2.5 and G4 (By similarity) |
| *A25R | Viral transcription | DNA-templated transcription | DNA binding,DNA-directed 5'-3' RNA polymerase activity,metal ion binding,ribonucleoside binding | DNA-directed RNA polymerase complex,virion component | Part of the DNA-dependent RNA polymerase which catalyzes the transcription of viral DNA into RNA using the four ribonucleoside triphosphates as substrates. Responsible for the transcription of early, intermediate and late genes. DNA-dependent RNA polymerase associates with the early transcription factor (ETF), itself composed of D6 and A7, thereby allowing the early genes transcription. Late transcription, and probably also intermediate transcription, require newly synthesized RNA polymerase (By similarity). |
| **D4L | Virokine |  |  |  |  |
| *A45L | Viral morphogenesis | steroid biosynthetic process | 3-beta-hydroxy-delta5-steroid dehydrogenase activity |  |  |
| *A40L | Cell-cell spread | positive regulation of cell-cell adhesion,positive regulation of inflammatory response,positive regulation of phagocytosis | thrombospondin receptor activity | integral component of plasma membrane |  |
| **B21R | Virostealth |  |  | integral component of membrane |  |
| **C2L | Virotransducers |  | serine-type endopeptidase inhibitor activity | extracellular space |  |
| *G6R | Viral transcription | DNA-templated transcription | DNA binding,DNA-directed 5'-3' RNA polymerase activity | DNA-directed RNA polymerase complex |  |
| **C20L | Virotransducers |  |  |  |  |
| **A49R | Virotransducers | suppression by virus of host NF-kappaB cascade |  | host cell cytoplasm,host cell nucleus | Plays a role in the inhibition of host NF-kappa-B activation. Interacts with host BTRC and thereby diminishes ubiquitination of NF-kappa-B inhibitor alpha/NFKBIA. This stabilizes NFKBIA and its interaction with NF-kappaB, so retaining p65/RELA in the cytoplasm and preventing NF-kappa-B-dependent gene expression. |
| *A21L | Viral entry | membrane fusion involved in viral entry into host cell |  | Membrane,virion membrane | Envelope protein part of the entry-fusion complex responsible for the virus membrane fusion with host cell membrane during virus entry. |
| *A33R | cell-cell spread |  |  | host cell membrane,integral component of membrane,viral envelope,virion membrane | Coordinates the incorporation of A36 into wrapped enveloped virion (EV) membranes and, subsequently, the production of actin tails. Therefore plays an essential role in efficient cell-to-cell spread of viral particles. |
| **I1L | Virotransducers | protein dephosphorylation,suppression by virus of host JAK-STAT cascade via inhibition of STAT1 activity,suppression by virus of host type I interferon-mediated signaling pathway | protein serine/threonine phosphatase activity,protein tyrosine phosphatase activity,protein tyrosine/serine/threonine phosphatase activity | host cell cytoplasm,virion component |  |
| **C8L | Virotransducers | dUMP biosynthetic process,dUTP catabolic process | dUTP diphosphatase activity,magnesium ion binding |  | This enzyme is involved in nucleotide metabolism: it produces dUMP, the immediate precursor of thymidine nucleotides and it decreases the intracellular concentration of dUTP so that uracil cannot be incorporated into DNA. |
| **A46R | Virotransducers | suppression by virus of host NF-kappaB cascade,suppression by virus of host viral-induced cytoplasmic pattern recognition receptor signaling pathway via inhibition of IRF3 activity |  |  | BCL2-like protein which disrupts the host immune response by inhibiting the TLR4 signaling pathway leading to NF-kappa-B activation. Acts close to the plasma membrane and targets several host TIR-domain containing adaptor proteins including MYD88, TIRAP, TRIF and TICAM2. In turn, blocks the host NF-kappa-B and TRIF-mediated IRF3 activation. |
| **B13R | Virotransducers | suppression by virus of host cysteine-type endopeptidase activity involved in apoptotic process | serine-type endopeptidase inhibitor activity | extracellular space,host cell cytoplasm | Inhibits the proteolytic activity of interleukin 1-beta converting enzyme (ICE) and ICE-like enzymes. Can also block apoptosis through host tumor necrosis factor (TNF) receptor. |
| *P2L | Viral transcription |  |  |  |  |
| **B4R | Virotransducers | suppression by virus of host innate immune response | 2',3'-cyclic GMP-AMP binding,nuclease activity |  | Nuclease that is responsible for viral evasion of host cGAS-STING innate immunity (PubMed:30728498). Cleaves 2',3'-cGAMP which is produced by host cGAS following recognition of intracellular foreign DNA and blocks the subsequent 2',3'-cGAMP-mediated activation of TMEM173/STING which normally spreads to adjacent cells and activates the interferon and NF-kappa-B immune responses |
| *D13L | Viral morphogenesis | response to antibiotic | identical protein binding | membrane | Scaffold protein which forms a transitory spherical honeycomb lattice providing curvature and rigidity to the convex membrane of crescent and immature virions (IV). This association occurs concomitantly with viral membrane formation. Targeted by the drug rifampicin, which prevents the formation of this lattice, and hence virus morphogenesis. In the presence of rifampicin, irregularly shaped membranes that lack the honeycomb layer accumulate around areas of electron-dense viroplasm. This layer is lost from virions during maturation from IV to mature virion (MV), through the proteolysis of A17 N-terminus. |
| **D18L | Virokine |  |  |  | Kelch-like protein |
| *D2L | Viral morphogenesis |  |  | virion component | Late protein which is part of a large complex required for early virion morphogenesis. This complex participates in the formation of virosomes and the incorporation of virosomal contents into nascent immature virions (By similarity). |
| *A51R | Viral transcription |  |  |  |  |
| **D3R | Virokine | regulation of epidermal growth factor-activated receptor activity | epidermal growth factor receptor binding,growth factor activity | extracellular region,integral component of membrane |  |
| **N4R | Virotransducers |  |  |  |  |
| *A7L | Viral transcription | positive regulation of DNA-templated transcription | DNA binding | virion component | Acts with RNA polymerase to initiate transcription from early gene promoters. Is recruited by the RPO-associated protein of 94 kDa (RAP94) to form the early transcription complex, which also contains the core RNA polymerase. ETF heterodimer binds to early gene promote |
| **B6R | virokine | negative regulation of complement activation | complement binding | integral component of membrane |  |
| *A3L | Virion maturation |  |  | virion component | Major component of the virion core that undergoes proteolytic processing during the immature virion (IV) to mature virion (MV) transition. Essential for the formation of a structurally normal core (By similarity). |
| **D15L | Virotransducers |  |  |  |  |
| **P1L | Virotransducers | mitigation of host antiviral defense response,modulation by virus of host apoptotic process |  |  |  |
| *A9R | Viral transcription |  |  |  | Acts with RNA polymerase to initiate transcription from intermediate gene promoters. |
| *B11R | Biochemical protein | protein phosphorylation | ATP binding,protein kinase activity |  |  |
| *E7R | Viral transcription | viral transcription | DNA binding,DNA-directed 5'-3' RNA polymerase activity | DNA-directed RNA polymerase complex,virion component |  |
| *H7R | Viral morphogenesis |  |  | integral component of membrane | Contributes to the formation of crescents and immature virions (IV). |
| **B7R | Virokine |  |  |  | Virulent,ER resident |
| *A47R | intracellular spread |  |  |  |  |
| *E5R | Viral replication |  | DNA binding | host cell cytoplasm | Major early protein present in virus factories. |
| *A50R | Viral replication-cell cycle | cell cycle,cell division,DNA biosynthetic process,DNA recombination,DNA repair,DNA replication | ATP binding,DNA binding,DNA ligase (ATP) activity,metal ion binding | host cell cytoplasm | DNA ligase that seals nicks in double-stranded DNA during DNA replication, DNA recombination and DNA repair. Recruits cellular topoisomerase II to sites of viral replication and assembly. |
| *L5L | Viral entry | fusion of virus membrane with host plasma membrane,viral entry into host cell |  | integral component of membrane,viral envelope,virion membrane | Envelope protein part of the entry-fusion complex responsible for the virus membrane fusion with host cell membrane during virus entry. Also plays a role in cell-cell fusion (syncytium formation) (By similarity) |
| *A28L | Viral entry | membrane fusion involved in viral entry into host cell,viral entry into host cell |  | integral component of membrane,viral envelope,virion membrane | Envelope protein required for virus entry into host cell and for cell-cell fusion (syncytium formation). |
| *I2L | Viral entry | viral entry into host cell |  | integral component of membrane,virion membrane | Late protein which probably plays a role in virus entry into the host cell |
| *I3L | Viral replication | chromosome condensation,viral DNA genome replication | single-stranded DNA binding | host cell cytoplasm | Plays an essential role in viral DNA replication. Binds to ssDNA with high affinity and localizes to cytoplasmic factories where nascent viral genomes accumulate. May disrupt loops, hairpins and other secondary structures present on ssDNA to reduce and eliminate pausing of viral DNA polymerase at specific sites during elongation. |
| *F2L | Viral replication | dUMP biosynthetic process,dUTP catabolic process | dUTP diphosphatase activity,magnesium ion binding |  | This enzyme is involved in nucleotide metabolism: it produces dUMP, the immediate precursor of thymidine nucleotides and it decreases the intracellular concentration of dUTP so that uracil cannot be incorporated into DNA. |
| **C4L | Virotransducers | suppression by virus of host NF-kappaB cascade |  | host cell cytoplasm,host cell nucleus | Plays a role in the inhibition of host NF-kappa-B activation. Mechanistically, blocks the subunit p65/RELA translocation into the host nucleus. |
| *D10L | Virotransducers | viral process |  |  | Plays a role for multiplication of the virus in different cell types(Host-range genes) |
| **F3L | Virotransducers |  |  |  | Might have a role in the suppression of host immune response |
| *E8L | Viral entry | viral entry into host cell,virion attachment to host cell | carbonate dehydratase activity,zinc ion binding | Cytoplasm,integral component of membrane,viral envelope,virion membrane | Binds to chondroitin sulfate on the cell surface to provide virion attachment to target cell. |
| *A42R | intracellular spread |  | actin binding |  | More likely to influence phosphoinositide metabolism than actin assembly(Profilin homolog) |
| **B3R | virotransducers | protein phosphorylation | ATP binding,protein kinase activity |  |  |
| *M1R | Viral morphogenesis |  |  | integral component of membrane,viral envelope,virion membrane |  |
| **C10L | Virotransducers |  |  |  | IL-1 beta antagonist |
| *L4R | Viral morphogenesis |  | structural molecule activity | viral capsid | Major core structural protein. |
| *D8L | Viral entry | viral entry into host cell,virion attachment to host cell | carbonate dehydratase activity,zinc ion binding | integral component of membrane,viral envelope,virion membrane | Binds to chondroitin sulfate on the cell surface to provide virion attachment to target cell. |
| **B12R | Virotransducers | protein phosphorylation | ATP binding,protein serine/threonine kinase activity | host cell nucleus | Pseudokinase that plays a role in viral DNA replication repression by activating the antiviral protein BANF1 and inhibiting the activity of host VRK1, a cellular modulator of BANF1. Both catalytically active kinases B1/VPK1 and host VRK2 repress B12 inhibitory activity in a B1/VPK1 deletion mutant strain.(ser/thr kinase) |
| *E1R | Viral replication |  | inorganic triphosphate phosphatase activity,metal ion binding,mRNA (guanine-N7-)-methyltransferase activity,mRNA guanylyltransferase activity,polynucleotide 5'-phosphatase activity,RNA binding |  | Catalytic subunit of the mRNA capping enzyme which catalyzes three enzymatic reactions: the 5' triphosphate end of the pre-mRNA is hydrolyzed to a diphosphate by RNA 5' triphosphatase; the diphosphate RNA end is capped with GMP by RNA guanylyltransferase and the GpppN cap is methylated by RNA (guanine-N7) methyltransferase. Heterodimeric mRNA capping enzyme catalyzes the linkage of a N7-methyl-guanosine moiety to the first transcribed nucleotide (cap 0 structure), whereas the polymerase associated VP39 is responsible for a second methylation at the 2'-O position of the ribose (cap 1 structure). The heterodimeric enzyme is also involved in early viral gene transcription termination and intermediate viral gene transcription initiation. Early gene transcription termination requires the termination factor VTF, the DNA-dependent ATPase NPH-I and the Rap94 subunit of the viral RNA polymerase, as well as the presence of a specific termination motif. Binds, together with RAP94, to the termination motif 5'-UUUUUNU-3' in the nascent early mRNA |
| **C7L | Virotransducers | suppression by virus of host innate immune response |  |  | Inhibits antiviral activity induced by type I interferons. Does not block signal transduction of IFN, but is important to counteract the host antiviral state induced by a pre-treatment with IFN.(Virulence factor) |
| *A22R | Viral replication | DNA recombination,DNA repair | four-way junction DNA binding,magnesium ion binding,nuclease activity |  | Plays a role in DNA replication by cleaving viral DNA concatamers to yield unit-length viral genomes. The concatamer junctions contain inverted repeat sequences that can be extruded as cruciforms, yielding Holliday junctions that A22 protein cleaves (By similarity) |
| *L6R | Viral replication | DNA-templated transcription | DNA binding,DNA-directed 5'-3' RNA polymerase activity | DNA-directed RNA polymerase complex |  |
| A44R | unknown |  |  |  |  |
| **C6R | Virotransducers | mitigation of host antiviral defense response |  |  |  |
| *Q2L | Viral |  | glutathione oxidoreductase activity,protein-disulfide reductase (glutathione) activity |  |  |
| **J1L | Virokine |  |  |  |  |
| **N3R | Virotransducers |  |  |  |  |
| **C22L | Viromimicry |  |  |  | The protein is truncated in this strain and presumably inactive. It has similarities with variola virus CrmB, but the product is truncated due to several premature stop codon. |
| *A19R | Viral replication |  | ATP binding,DNA binding,hydrolase activity | virion component |  |
| **D1L | Virokine |  |  |  | Ankyrin like protein |
| *H5R | Viral transcription | viral DNA genome replication,viral transcription | translation elongation factor activity | host cell cytoplasm,viral envelope | Involved in the co-transcriptional or post-transcriptional endoribonucleolytic cleavage that generates sequence-homogeneous 3' ends during late transcription. Involved in postreplicative transcription elongation on intermediate and late genes (Probable). Also involved in DNA replication and in multiple steps of virion morphogenesis. Required both for inclusion of virosoplasm into crescents as well as for maturation of immature virions (IV) into mature virions (MV) |
| **C17L | Virokine |  |  |  |  |
| **C21L | Virotransducers |  |  |  |  |
| *A34L | Viral replication |  |  | integral component of membrane |  |
| *C1L | Virotransducers |  |  |  | Ankyrin like protein |
| **M3L | Virotransducers |  |  |  |  |
| *I4L | Viral replication | DNA replication | ATP binding,ribonucleoside-diphosphate reductase activity, thioredoxin disulfide as acceptor |  | Provides the precursors necessary for DNA synthesis. Catalyzes the biosynthesis of deoxyribonucleotides from the corresponding ribonucleotides. |
| **C11L | Virotransducers | viral process |  | integral component of membrane |  |
| **J3R | Virokine | 7-methylguanosine mRNA capping,regulation of mRNA 3'-end processing | mRNA (nucleoside-2'-O-)-methyltransferase activity,RNA binding,translation elongation factor activity | virion component | Displays methyltransferase, positive regulation of the poly(A) polymerase and transcription elongation activities. Involved in the modification of both mRNA ends and in intermediate and late gene positive transcription elongation. At the mRNAs 5' end, methylates the ribose 2' OH group of the first transcribed nucleotide, thereby producing a 2'-O-methylpurine cap. At the 3' end, functions as a processivity factor which stimulates the activity of the viral poly(A) polymerase VP55 that creates mRNA's poly(A) tail. In the presence of VP39, VP55 does not dissociate from the RNA allowing tail elongation to around 250 adenylates. |
| **B2R | Virotransducers | suppression by virus of host innate immune response | 2',3'-cyclic GMP-AMP binding,nuclease activity |  | Nuclease that is responsible for viral evasion of host cGAS-STING innate immunity (PubMed:30728498). Cleaves 2',3'-cGAMP which is produced by host cGAS following recognition of cytosolic DNA and blocks the subsequent 2',3'-cGAMP-mediated activation of TMEM173/STING, which normally spreads to adjacent cells and activates the interferon and NF-kappa-B immune responses (PubMed:30728498). |

**^** - This symbol represents the immune-evasion function * - This symbol represents the Viral Transcriptional or other related functions^**

# Supplementary Figures:

**Figure S1: Statistical plots of the dataset GSE219036 in human colon organoids from several Monkeypox clades**. A) The boxplot mentioned the total read counts for the 12 samples. B) The heatmap of Mock vs. various strains was displayed; red colors indicated up-regulation, and green colors indicated down-regulation. C) The boxplot mentioned the transformed data distribution and the expression density of the 12 samples. D) Principal Component Analysis was carried out, and various strains were represented in various ways.


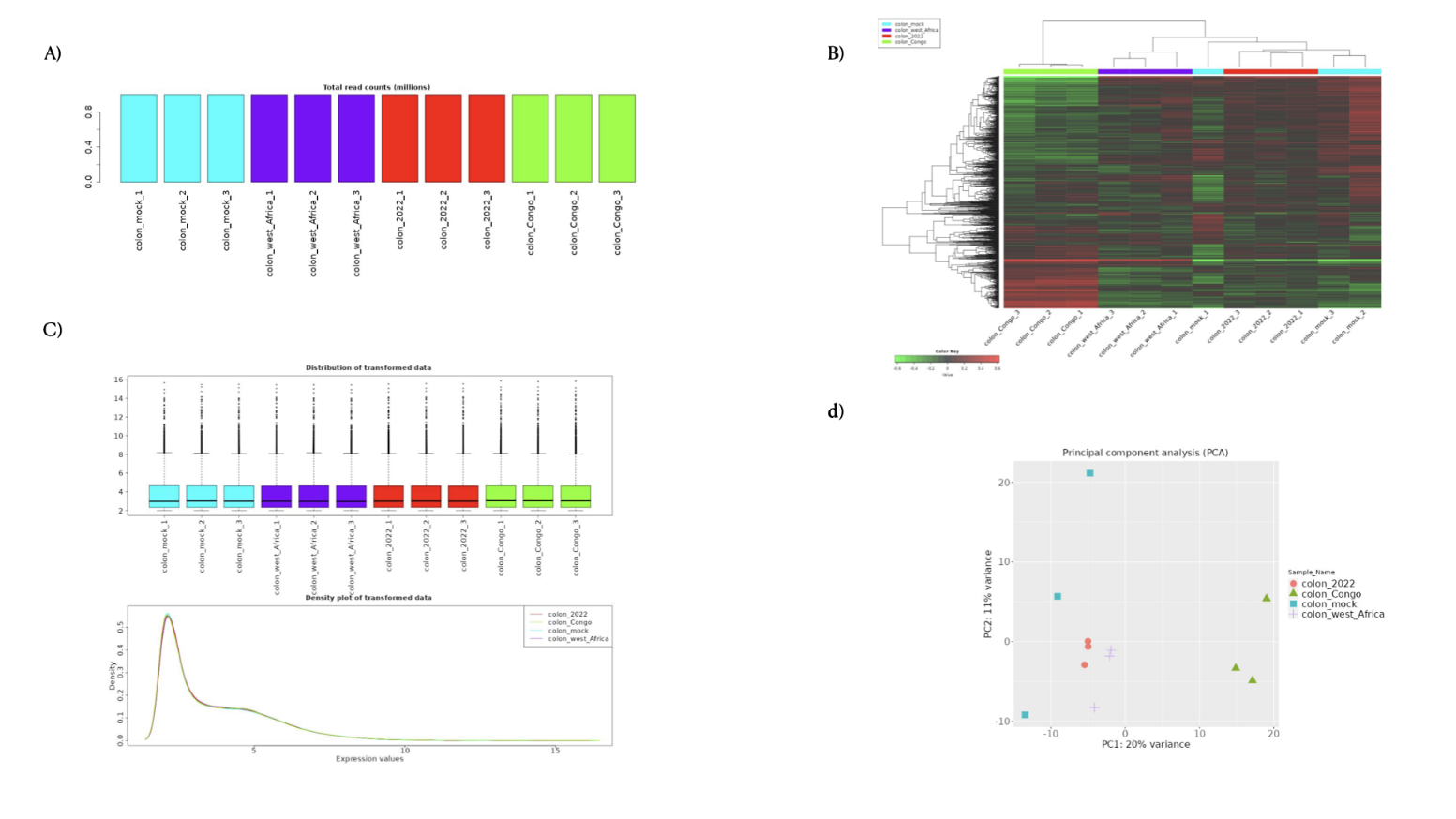


**Figure S2: Statistical plots of the dataset GSE219036 in human keratinocytes from distinct Monkeypox clades**. A) The boxplot mentioned the total read counts for the 12 samples. B) The heatmap of Mock vs. different strains was displayed; red colors indicated up-regulation, and green colors indicated down-regulation. C) The boxplot included information on the distribution of the transformed data and the expression density of the 12 samples. D) Principal Component Analysis was used, and the various strains were represented in various ways


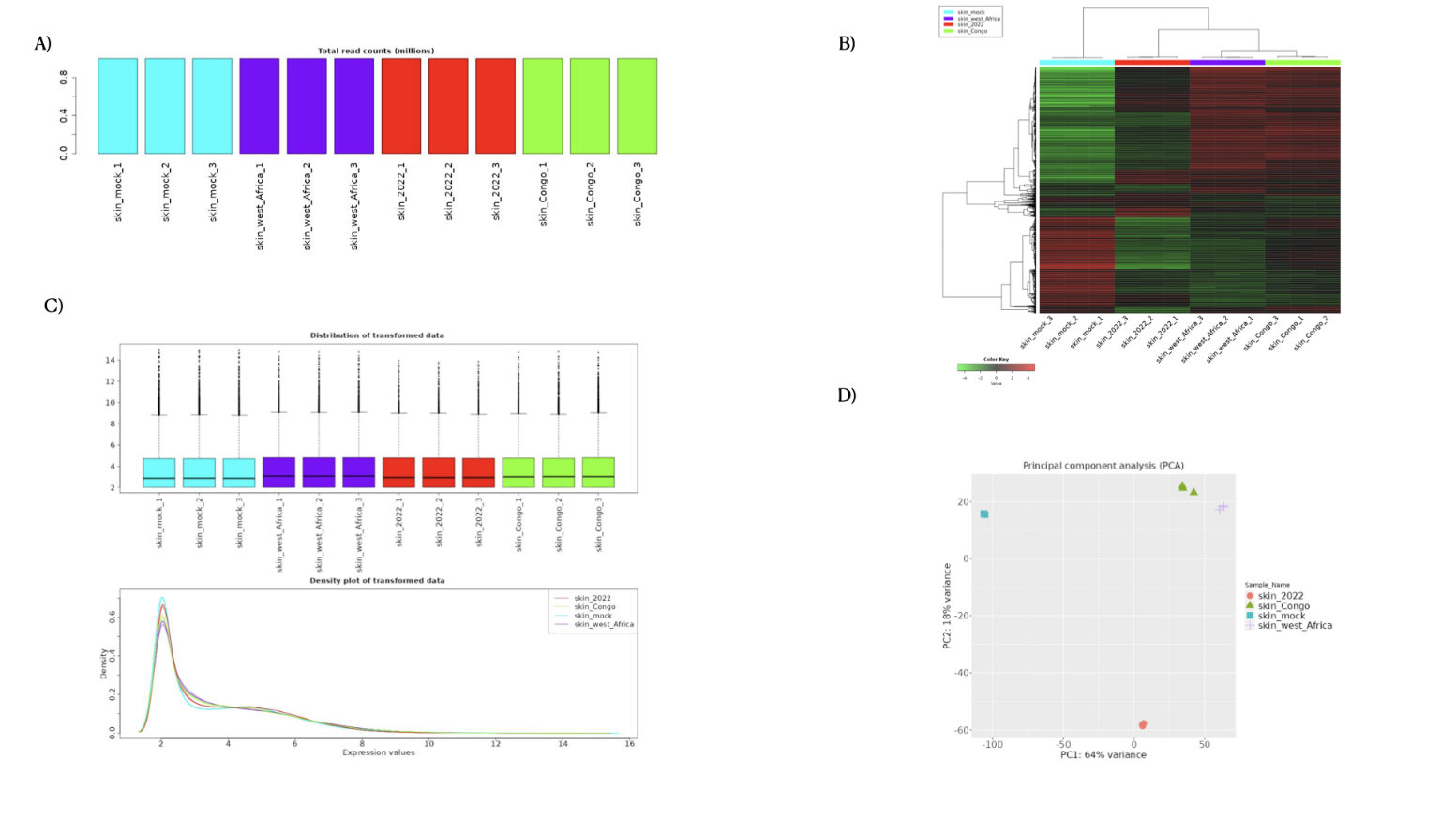


**Figure S3: Protein-protein interaction in different Hosts (GSE36854 and GSE21001).** A) Protein-Protein interaction network of Monkeypox differential expressed genes from Host *Homo sapiens* (111). B) Protein-Protein interaction network of Monkeypox differential expressed genes from Host *Macaca mulatta* (50). C) Protein-protein interaction network of Cowpox differentially expressed genes from Host Human (217). D) Protein-Protein interaction network of Vaccinia differential expressed genes from Host *Homo sapiens* (162). There were two important clusters from all the PPI: Histones and Immune-related genes. The important connection genes between histones and immune genes were highlighted in green in different PPI interactions. All these figures were generated using the Cytoscape tool.


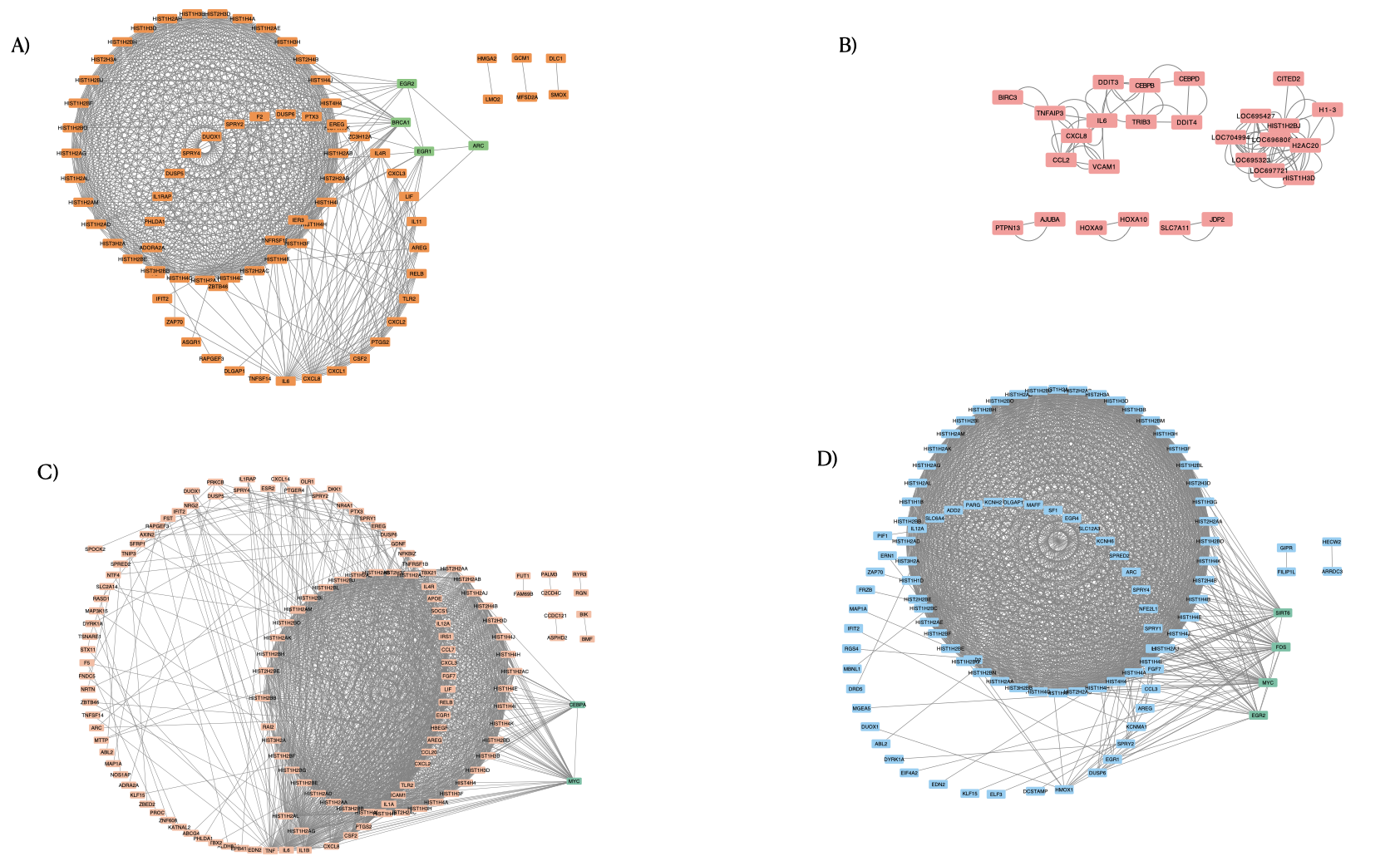


**Figure S4**: **Merged network of pox infections from Host (*Homo sapiens* ).** This is the merged network of all Pox infections in the host. All Pox infections have different clusters, but most predominantly, histones and immune-related genes were enriched. These figures were generated using the Metascape tool.


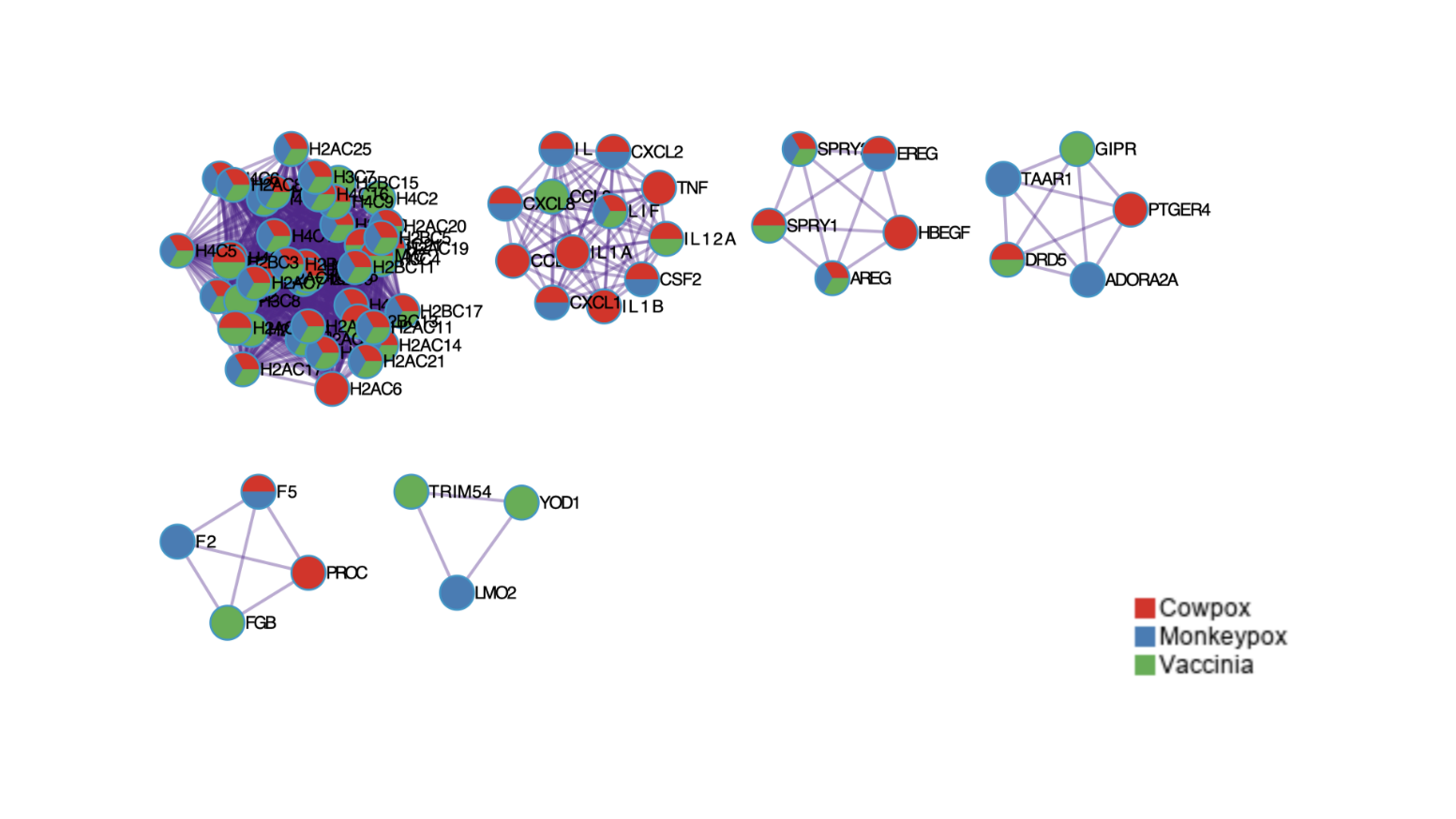


**Figure S5: Structural Similarities between K7R (Vaccinia) and C6R derived protein K7 (MPXV) proteins**. These results depicted the structural similarity between two homologous proteins, K7R (Vaccinia) and C6R derived protein K7 (MPXV), that have major important connections between epigenetic and immune mechanisms. A) Superimpose of K7R 3D structure and C6R derived protein K7 3D structure B) Pair-wise sequence alignment between these viral proteins. The results were generated using an online tool (<https://www.ebi.ac.uk/Tools/psa/>).


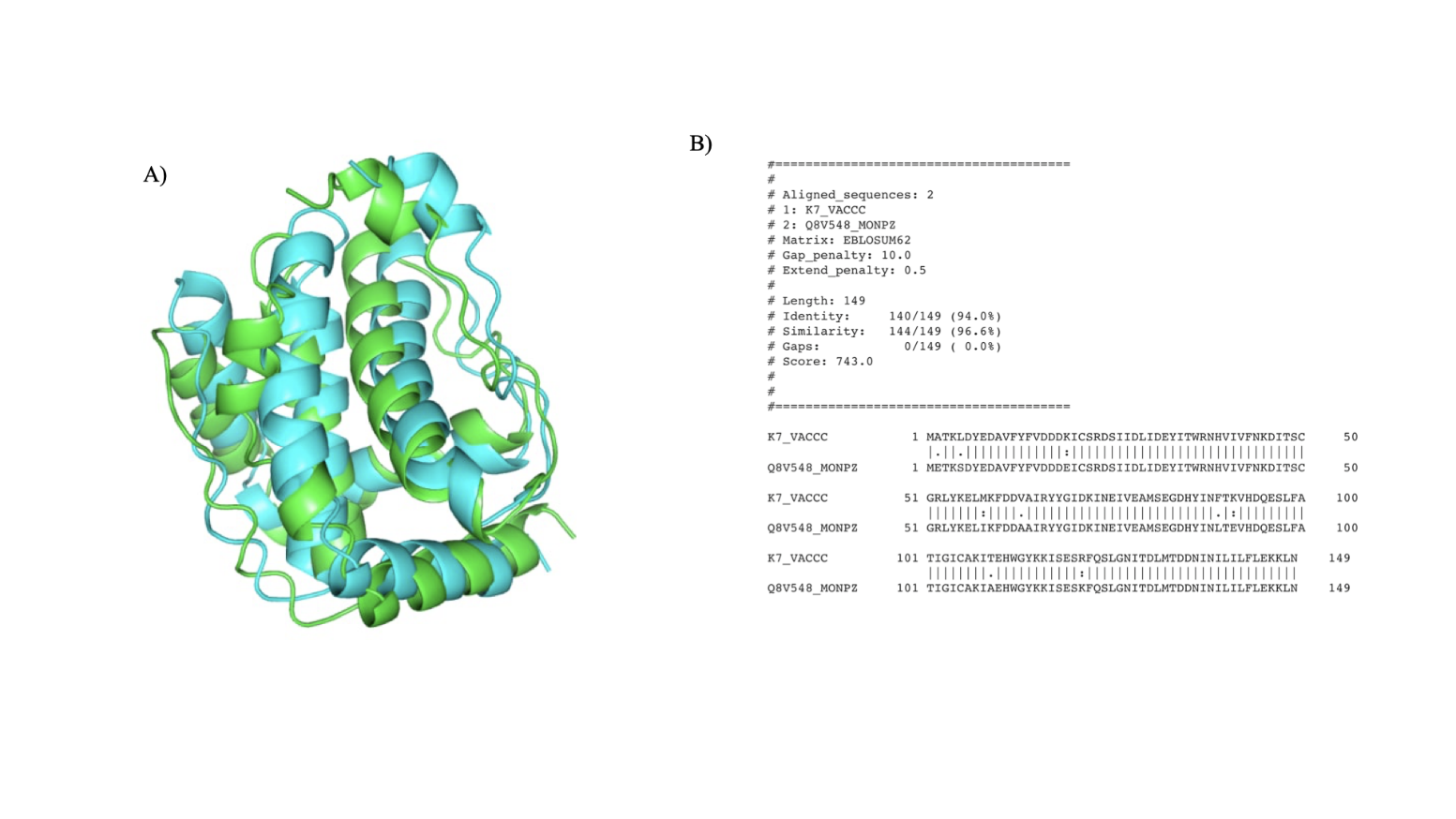


**Figure S6: Virtual screening workflow**. This workflow depicted the screening of different algorithms and screened the potential lead molecule against putative targets.


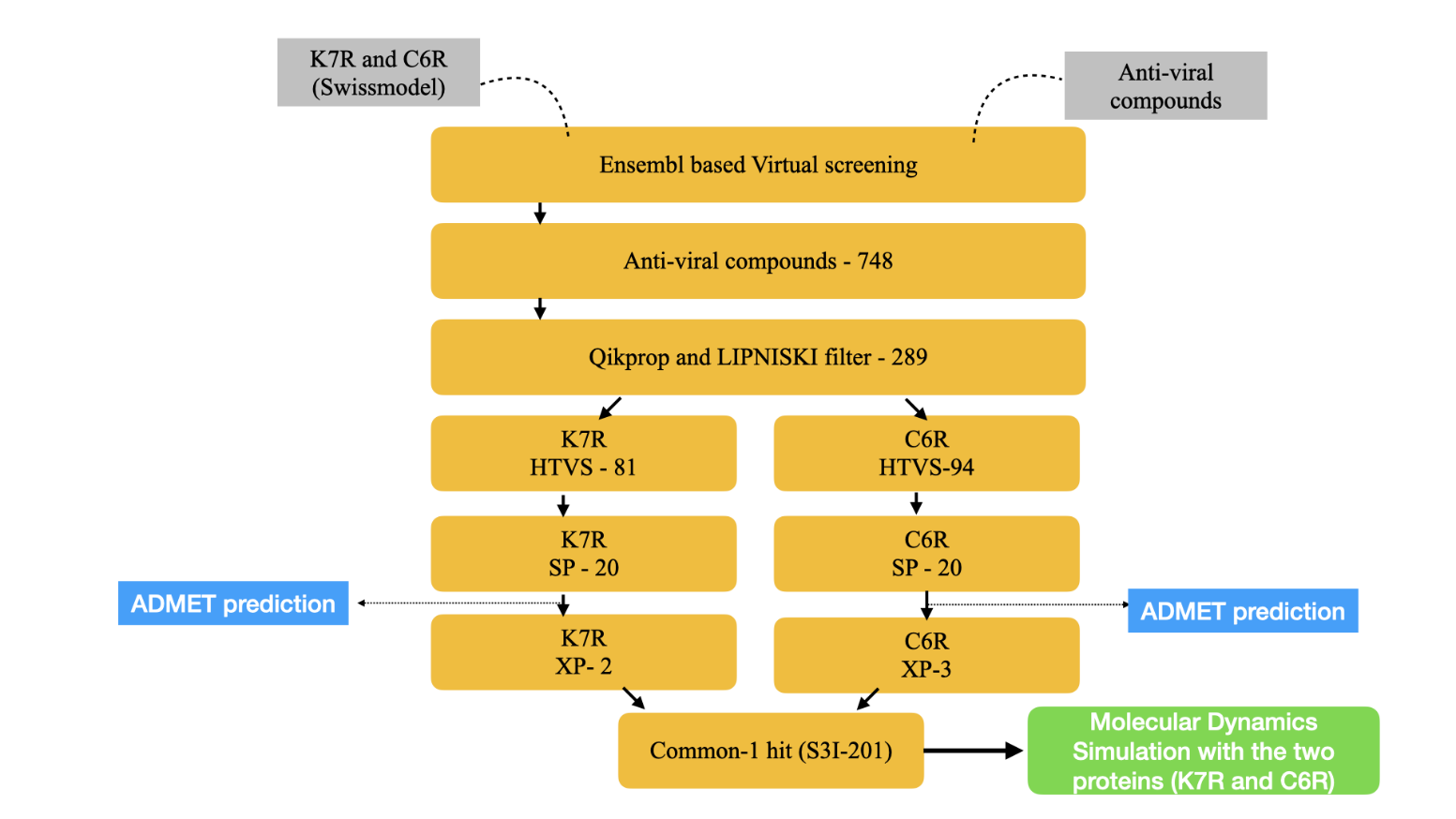

Supplement: Supplementary file 1 — Supplementary Material 1. [file 12879_2024_9332_MOESM1_ESM.docx]
